# Impact of honey on post‐tonsillectomy pain in children (BEE PAIN FREE Trial): a multicentre, double‐blind, randomised controlled trial*

**DOI:** 10.1111/anae.16619

**Published:** 2025-05-05

**Authors:** David Sommerfield, Aine Sommerfield, Daisy Evans, Neil Hauser, Shyan Vijayasekaran, Paul Bumbak, Hayley Herbert, Cornelia Locher, Lee Yong Lim, R. Nazim Khan, Britta S. von Ungern‐Sternberg

**Affiliations:** ^1^ Department of Anaesthesia and Pain Medicine Perth Children's Hospital Nedlands WA Australia; ^2^ Division of Emergency Medicine, Anaesthesia and Pain Medicine Medical School, The University of Western Australia Perth WA Australia; ^3^ Peri‐operative Medicine Team, Peri‐operative Care Program Telethon Kids Institute Nedlands WA Australia; ^4^ Institute for Paediatric Peri‐operative Excellence, The University of Western Australia Perth WA Australia; ^5^ School of Physics, Mathematics and Computing, The University of Western Australia Crawley WA Australia; ^6^ Department of Otolaryngology/Head and Neck Surgery Perth Children's Hospital Nedlands WA Australia; ^7^ Division of Pharmacy School of Allied Health, The University of Western Australia Crawley WA Australia; ^8^ Department of Mathematics and Statistics The University of Western Australia Crawley WA Australia

**Keywords:** anaesthesia, outcome, paediatric, pain, tonsillectomy

## Abstract

**Introduction:**

Tonsillectomy, a common childhood surgery, is associated with difficult postoperative recovery. Previous reviews provided low‐grade evidence that honey may improve recovery. The BEE PAIN FREE study investigated whether honey alongside multimodal analgesia improved the recovery trajectory in children following tonsillectomy.

**Methods:**

A prospective randomised controlled trial was conducted across three centres in Western Australia. Children undergoing extracapsular tonsillectomy by coblation were allocated randomly to one of four postoperative treatment groups: standard treatment alone; Marri honey (from Western Australia); Manuka honey (from Western Australia); or placebo. The intervention groups took 5 ml of honey or placebo, six times a day, for at least 7 days, in addition to usual discharge analgesia (standard treatment). Data for daily pain scores, Parents' Postoperative Pain Measure scores, medications and unplanned re‐presentations were collected.

**Results:**

A total of 400 children were recruited; 20% were lost to follow‐up or withdrew. The mean number of honey doses taken varied between 2 and 3 doses per day over 7 days. Treatment with honey at this frequency did not impact postoperative pain scores significantly, with all groups showing similar trajectories. These findings did not alter with as‐treated analysis or using imputed models for missing data. Most children experienced significant pain until around postoperative day 8. Children allocated to the honey and placebo groups showed some improved oral tolerance around day 6 but had increased vomiting during earlier days. There were no clinically significant differences in medical re‐presentations, simple analgesia or oxycodone usage between groups.

**Discussion:**

Two to three doses daily of oral honey/placebo in children post‐extracapsular tonsillectomy for 7 days, in addition to regular paracetamol, ibuprofen and as required oxycodone did not result in a clinical improvement in pain or recovery over a 14‐day follow‐up period.

## Introduction

Tonsillectomy remains one of the most common childhood surgical procedures [1]. Recovery is often difficult with pain (particularly on swallowing), poor oral intake and risk of haemorrhage and infection [2]. For many children, pain after tonsillectomy represents the worst part of the experience [3]. Studies report medical representations as high as 50–70% for pain [4, 5]. Factors including the risk of haemorrhage [6] or an increased risk of respiratory events [7] complicate post‐tonsillectomy pain management. Medication compliance is a contributing factor to poor pain management, due to misperceptions of parents and reluctance of children to take medications, often exacerbated by poor palatability or adverse effects [8].

Honey is a natural product with antimicrobial and anti‐inflammatory bioactivities. Its medical use dates to the ancient Egyptians [9, 10]. Antimicrobial properties of honeys are associated with their high osmolarity (up to 21% water content and 60–85% sugar content) and low pH. Honeys derived from certain floral sources exceed these baseline effects [11], for example in peroxidase honeys due to hydrogen peroxide and in non‐peroxide honeys (e.g. Manuka) due to methyl glyoxal [12].

A recent review reported that six of eight randomised controlled trials showed improved pain scores in children using honey post‐tonsillectomy [13]. A systematic review of 64 studies found that honey reduced pain and analgesic use, but the grade of the evidence was ‘low’ to ‘very low’ [14]. Furthermore, in many of these studies, paracetamol was the only analgesic. Many centres include anti‐inflammatories and/or opioids as part of their discharge medications [15]. Therefore, there is a need to study honey in the setting of multimodal analgesia post‐discharge. Another question, unaddressed in previous studies, is whether the observed beneficial effect of honey stems from its inherent bioactivity or is simply due to its demulcent or coating effect.

We designed a multicentre, double‐blinded, randomised controlled trial investigating the impact of two different honeys administered for 7 days on postoperative pain scores and the recovery trajectory in children following tonsillectomy compared with placebo (a sugar syrup with similar sugar content and consistency to honey) and standard care.

## Methods

This prospective randomised controlled trial was conducted across three centres in Western Australia: Perth Children's Hospital; Fiona Stanley Hospital; and St John of God Hospital Subiaco. Each centre had institutional ethics committee approval.

Children (aged 1–16 y) undergoing elective extracapsular tonsillectomy (+/‐ adenoidectomy; cautery of inferior turbinates; myringotomy; examination of the ear; insertion of grommets) were eligible for inclusion. Written informed consent was obtained from parents/guardians and also child agreement (where appropriate). Children were not included if they had an allergy to honey or the placebo ingredients, or were diabetic. Our study used two honeys, a Marri (*Corymbia callophylla*) and a Western Australian Manuka (*Leptospermum scoparium*), as well as a placebo ‘honey’ (composed of glucose syrup, rice malt syrup and PharmAust™ syrup BP (PharmAust Manufacturing, Malaga, WA, Australia) with no additives). They were characterised using standardised testing [16, 17, 18]. Patients were assigned randomly by an independent statistician using a computer‐generated block randomisation scheme, with random block sizes of 4 or 8, to receive standard treatment alone, or one of Marri honey, Western Australian Manuka honey or placebo alongside standard treatment. Identical containers of each honey and placebo were labelled with randomisation numbers by an independent researcher and the patient allocated to receive honey/placebo was given the container with the relevant randomisation number. Researchers, staff and families of the three intervention groups were blinded to group allocation.

Standard demographic, surgical and peri‐operative data, including the Snoring, Trouble Breathing Un‐Refreshed (STBUR) questionnaire were collected for all patients. The STBUR five‐question tool helps identify children at risk of sleep disordered breathing and their risk of peri‐operative adverse respiratory events [19].

The surgical technique used was coblation for extracapsular tonsillectomy. Local anaesthetic infiltration was usually given by surgeons. Anaesthesia was conducted in accordance with institutional guidelines. Induction of general anaesthesia was either incremental inhalation of sevoflurane (up to 8%, with nitrous oxide) or intravenous propofol (> 3 mg.kg^‐1^), with sevoflurane used for anaesthesia maintenance. Peri‐operative analgesia was standardised using paracetamol 15–20 mg.kg^‐1^ orally pre‐operatively or parecoxib intravenously intra‐operatively as per weight protocol. Intra‐operative opioids were used at the anaesthetist's discretion, accounting for patient comorbidities. The most commonly used intra‐operative opioids were fentanyl 1–2 μg.kg^‐1^ and/or morphine 0.05–0.1 mg.kg^‐1^; however, pethidine, hydromorphone and alfentanil were used occasionally. The intra‐operative opioid dose was converted to the oral morphine equivalent dose [20]. All children received dual peri‐operative anti‐emetics and Hartman's solution 10–20 ml.kg^‐1^ was given intra‐operatively.

All children stayed at least one night in hospital and were monitored with pulse oximetry, reflecting the normal course in the institutions. The treating team had the final discretion on discharge medications with the prescribed regimen: oral paracetamol 15 mg.kg^‐1^ four times daily for 7 days, then as required; oral ibuprofen 10 mg.kg^‐1^ three times daily for 7 days, then as required; and oral oxycodone between 0.05 and 0.1 mg.kg^‐1^ 6‐hourly as required. At least one dose of oral oxycodone had to be tolerated safely before discharge. Parents recorded medications in a medication diary. Compliance with simple analgesia was defined as taking three of the four available doses of paracetamol and two of the three available doses of ibuprofen on a given day. Once on the ward and tolerating food, each child received a test dose of either honey or placebo as per their assigned group. Children assigned to the honey or placebo groups were asked to take 5 ml of honey or placebo six times daily, preferably before eating. Children could continue with honey/placebo beyond the 7‐day intervention period.

Parents/guardians documented pain scores using a paper diary for 14 days postoperatively (online Supporting Information Appendix S1). Self‐reported pain scores were determined four times daily using the Face Pain Scale Revised (FPS‐R), twice to assess the background or ‘at rest’ pain intensity and twice with swallowing. Pain scores were collected in the morning around breakfast (AM rest and AM swallow), then repeated around the evening meal (PM rest and PM swallow). Parental proxies were used for children aged < 6 y [21]. Parents were also asked to complete a slightly modified Parents' Postoperative Pain Measure daily [22], with question 11, ‘*Try not to bump the sore area of his/her body*’ excluded as it was not relevant to the postoperative course following tonsillectomy. The 15‐item observational Parents' Postoperative Pain Measure tool has been validated following tonsillectomy, with a score of 6 signifying important pain (equivalent to FPS‐R of 2.8/10) and a score of 9 signifying significant pain (FPS‐R score of 6/10) [23]. Details of analgesic use and unplanned medical presentations were recorded for 14 days or until the child was pain‐free without analgesia. Parents were sent a REDCap link [24] each evening to transfer data from their diary. If 2 days in succession were missed, research staff would collect responses by telephone. One week following the daily questionnaires, carers were provided with a survey that recorded a global satisfaction score for treatment (using a numerical rating scale NRS‐11) and asked to evaluate which honey group they thought they were in and if honey had helped.

In this longitudinal study, data were analysed using a repeated measures model with pain as the response. As there are no analytic methods appropriate for power calculations in such complex models, simulations need to be conducted (or an online calculator could be used). The data for the treatment groups were simulated based on a mean difference in pain scores of 1 (deemed to be clinically significant). It was also expected that in all treatment groups the average pain scores would decrease over time, with the standard deviation assumed as constant over time. More specifically, over the seven time‐points the mean pain scores were taken as honey (10, 8, 6, 4, 2, 1, 1) and standard treatment (control) (10, 9, 8, 6, 5, 3, 3), with a standard deviation of 3 for all time‐points. Based on 500 simulations, the power was calculated as the proportion of times the null hypothesis was rejected, based on a repeated measures model for the pain scores. The simulations were repeated with several different parameter values, and a sample size of 85 per group was found to be sufficient. Simulations gave a power of 0.9 for a sample size of 85 per group. Fifteen children were added to each group to account for potential loss to follow‐up, cancellation of surgery, change of surgical plan and other protocol violations.

Analyses were performed in R (R Foundation for Statistical Computing, Vienna, Austria) [25]. Statistical significance was taken at 5% (p ≤ 0.05). Data were explored using numeric and graphical summaries. Outcomes were based on intention‐to‐treat. Multiple imputation by chained equations (MICE) was performed [26], which improves accuracy and statistical power relative to complete‐case analysis [27]. For details see online Supporting Information Appendix S2. To assess whether honey reduced FPS‐R pain scores, linear mixed models were fitted, with fixed‐effects covariates including: days since surgery (1–14, treated as a factor variable to allow for the non‐linear relationship); measurement condition (AM rest, AM swallow, PM rest, PM swallow); baseline and immediate postoperative pain measurements; surgical and patient‐specific characteristics; daily Parents' Postoperative Pain Measure scores; and analgesic drugs taken. Random intercepts for patients nested within sites were included to reflect the repeated measures design. Models for the complete‐case analysis and MICE datasets were compared. Models were reduced using backward stepwise selection based on the Akaike Information Criterion. For the multiply imputed datasets, variables significant in > 50% of models following variable reduction were retained (see online Supporting Information Appendix S2) [28].

## Results

**Figure 1:**
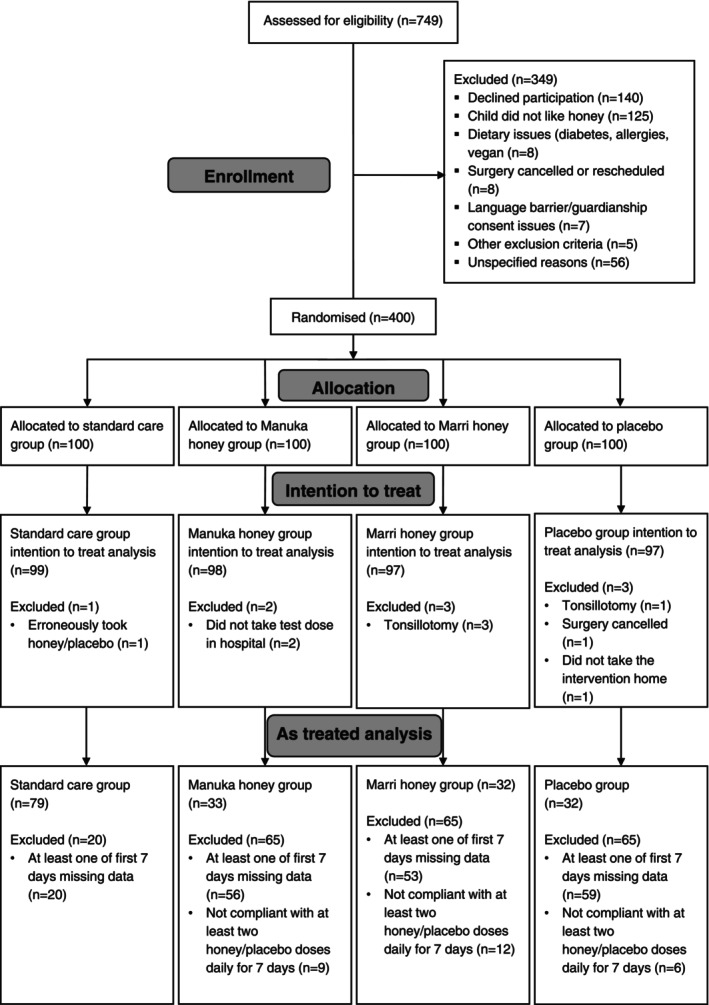
Study flow diagram.

Between November 2019 and July 2021, 749 children were screened for inclusion, of which 400 were recruited and allocated randomly to a treatment group (Fig. 1); nine children were subsequently removed from the intention‐to‐treat analysis. Of the 391 children included, 97% (n = 381) received a flexible supraglottic airway and 3% (n = 10) had a tracheal tube. Inhalational induction was used for 86% (n = 338). Patient characteristics and intra‐operative factors were reasonably uniform across groups (Table 1). The characterisation of the honeys was as expected (online Supporting Information Appendix S3), with the two natural honeys showing additional antibacterial and antioxidant activity to placebo.

**Table 1:** Patient characteristics and intra‐operative details children allocated to different treatment groups after tonsillectomy. Values are median (IQR[range]), number (proportion) or mean (SD).

	Standard care	Manuka honey	Marri honey	Placebo
	n = 99	n = 98	n = 97	n = 97
Age; y	5.3 (3.8–8.0 [1.7–16.3])	5.6 (3.6–8.3 [1.9–16.1])	5.9 (4.1–8.2 [1.3–15.9])	6.5 (4.5–8.5 [1.6–15.2])
Age				
≤ 4.99 y	46 (46%)	44 (45%)	36 (37%)	30 (31%)
5–6.99 y	14 (14%)	22 (22%)	23 (24%)	26 (27%)
7–8.99 y	20 (20%)	13 (13%)	20 (21%)	24 (25%)
≥ 9 y	19 (19%)	19 (19%)	18 (19%)	17 (18%)
Sex; female	42 (42%)	42 (43%)	43 (44%)	43 (44%)
BMI z‐score	0.81 (3.38)	0.77 (1.37)	0.88 (3.44)	0.60 (1.35)
STBUR score	5 (3–5 [0–5])	4 (2–5 [0–5])	4 (2–5 [0–5])	3 (2–5 [0–5])
ASA				
1	36 (37%)	46 (51%)	38 (41%)	44 (46%)
2	62 (63%)	45 (49%)	53 (57%)	51 (53%)
3	0	0	2 (2%)	1 (1%)
Missing	1	7	4	1
Intra‐operative OME.kg^‐1^	0.41 (0.12)	0.40 (0.12)	0.41 (0.14)	0.42 (0.26)

STBUR tool, (Snoring, Trouble Breathing, Un‐Refreshed); OME, oral morphine equivalent.

Twenty percent of children withdrew or were lost to follow‐up, with the highest loss (n = 25, 26%) in the placebo group and the lowest (n = 14, 14%) in the Manuka group. The mean number of daily doses of honey or placebo taken in each intervention group dropped from three to two doses per day, over the 7 days (online Supporting Information Appendix S4, Figure S1). Most children taking honey or placebo did not take it on at least one of the 7 days (see online Supporting Information Appendix S4, Figure S2 for the proportion of patients who reported taking honey or placebo each day). Over half of each group continued taking honey or placebo after day 7 (n = 49, 51% in the Marri honey group; n = 51, 53% in the placebo group; and n = 58, 59% in the Manuka honey group). When conducting the as‐treated analysis, we defined compliance as at least two documented doses of honey/placebo each day in the first 7 postoperative days. This reduced the sample size to n = 97 (n = 32 in the Marri honey group, n = 32 in the placebo group and n = 33 in the Manuka honey group). Younger children were less compliant with honey.

Variables measured longitudinally over the 14 days showed high rates of missingness, with the FPS‐R score at AM swallowing being highest (27%). Overall, 115 children (29%) had complete records across all variables. For detailed exploration of missing data see online Supporting Information Appendix S2.

**Figure 2:**
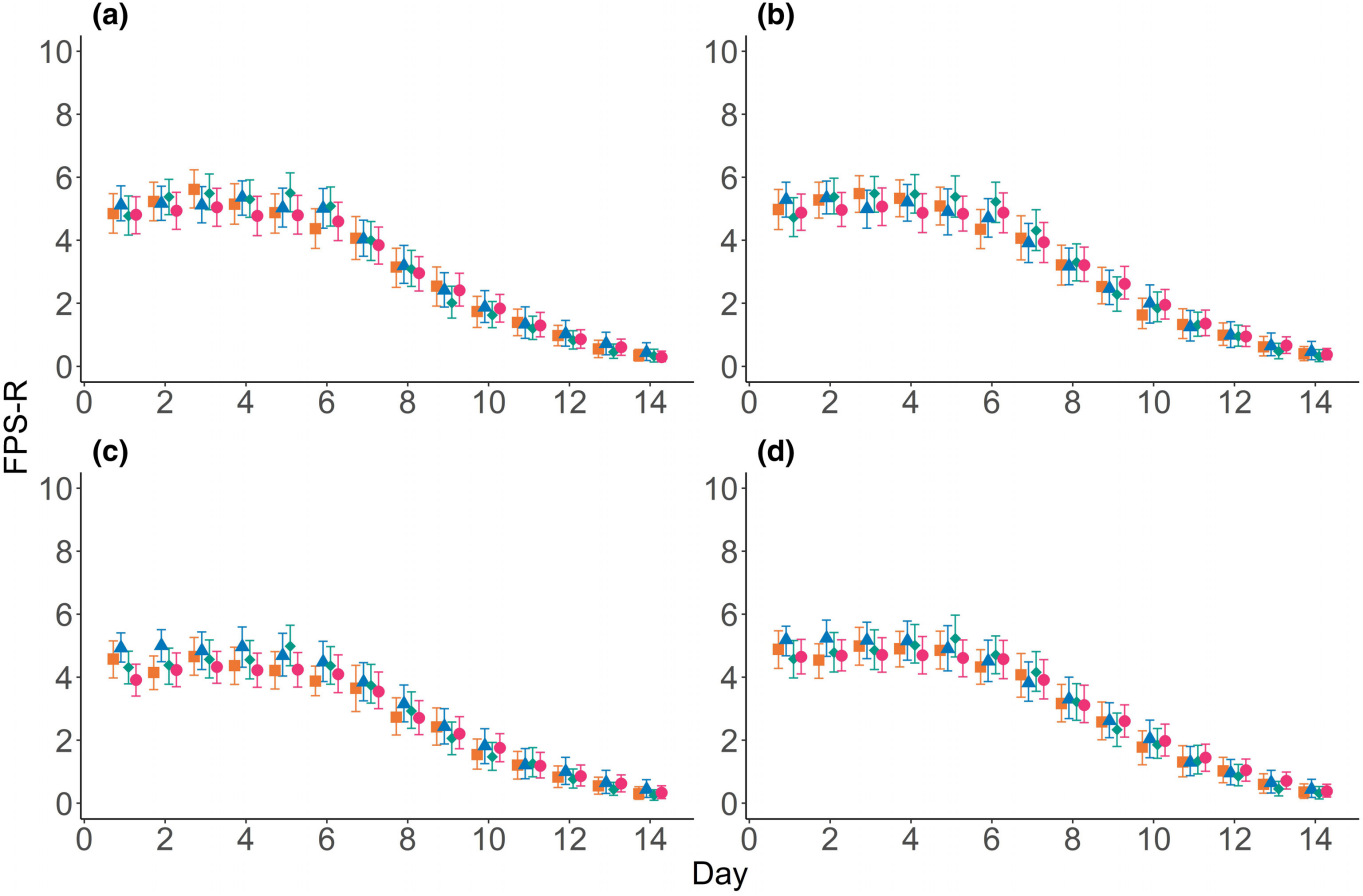
Mean pain scores for each treatment group at different time‐points during the 14 postoperative days. Data are mean with bootstrapped 95%CI. (a) AM rest scores; (b) AM swallow scores; (c) PM rest scores; (d) PM swallow scores. Orange squares, Manuka honey group; blue triangles, Marri honey group; green diamonds, placebo group; pink circles, standard care. Data are shown in online Supporting Information Appendix S4, Table S3. FPS‐R, Face Pain Scale Revised.

Mean FPS‐R scores (with and without swallowing) showed similar trajectories that overlap, all within 1 point of the scale, with no group consistently higher or lower than others (Fig. 2). For days 1–8 following surgery, across all groups, the mean FPS‐R scores were generally a little better in the evening compared with the morning and worse with evening swallowing (online Supporting Information Appendix S4, Figure S5).

**Figure 3:**
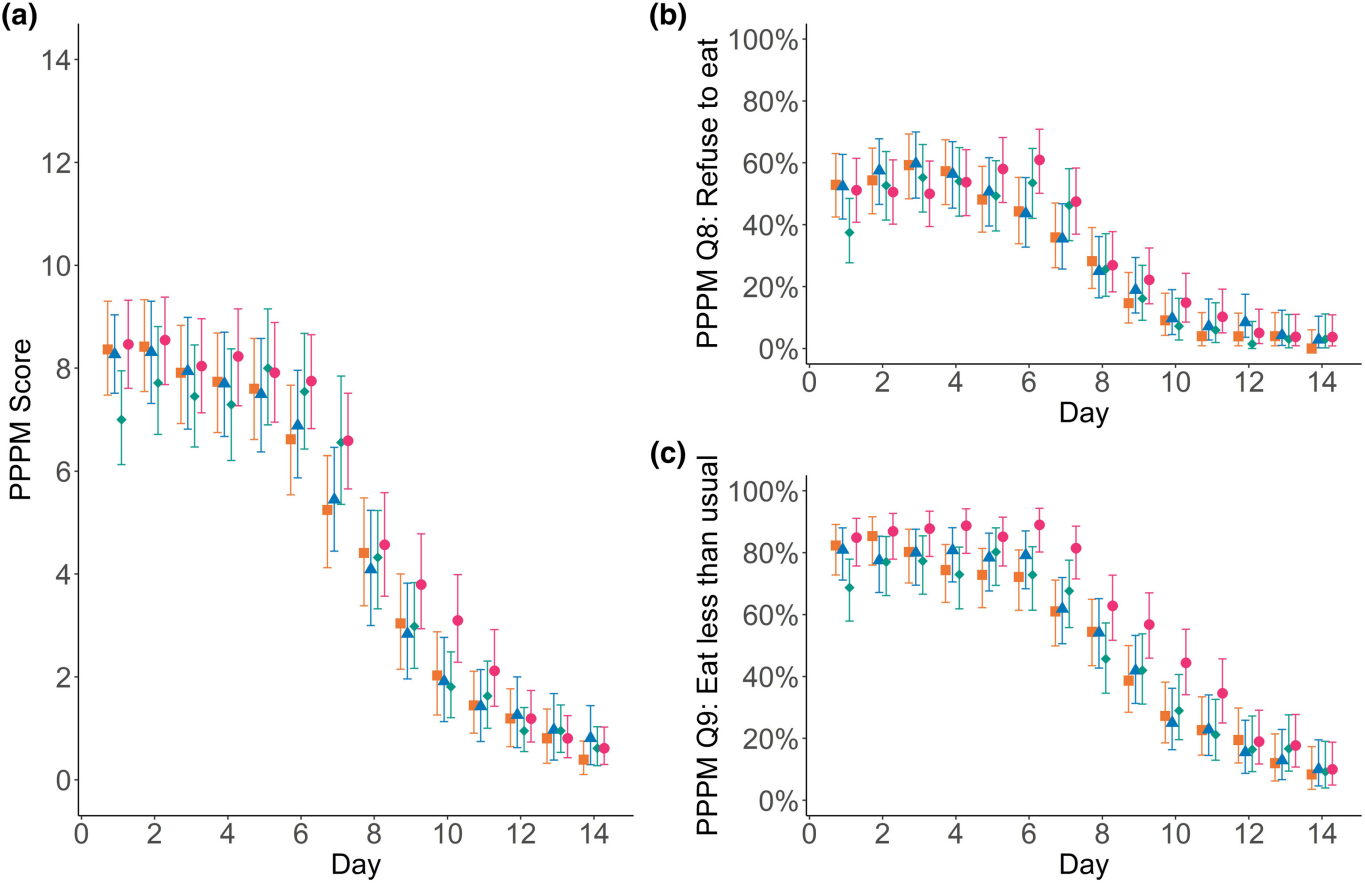
Parents' Postoperative Pain Measure (PPPM) scores for each treatment group. (a) mean scores; (b) proportion of patients selecting ‘Yes’ to PPPM question 8 each day; (c) proportion of patients selecting ‘Yes’ to PPPM question 9 each day. Orange squares, Manuka honey group; blue triangles, Marri honey group; green diamonds, placebo group; pink circles, standard care. Data are mean and bootstrapped 95%CI for (a) and mean with Agresti‐Coull 95%CI for (b) and (c). Data are shown in online Supporting Information Appendix S4, Table S4.

Mean daily Parents' Postoperative Pain Measure scores (Fig. 3a) reflected important (moderate) pain (≥ 6/14) in each group until around day 8 and scores > 9, representing severe pain, still present in around 25% of children on that day (Table 2). The standard care group tended to have a slightly higher score, but the mean separation between groups was usually < 1 point. The Parents' Postoperative Pain Measure scores and items of interest on day 7 are shown in Table 2. The small improvement seen in the Parents' Postoperative Pain Measure was accounted for mainly by an improvement in the questions directed at eating behaviours, suggesting better oral tolerance observed by parents in the honey/placebo groups, especially on days 5–7 (Fig. 3b and c). However, less nausea and vomiting were reported post‐discharge in the standard care group (online Supporting Information Appendix S4, Table S1 and Appendix S4, Figure S6). Return to normal play showed a similar trajectory across groups (see online Supporting Information Appendix S4, Figure S7).

**Table 2:** Key outcomes over the 14‐day postoperative follow‐up period for each treatment group. Values are number (proportion).

	Standard care	Manuka honey	Marri honey	Placebo
	n = 99	n = 98	n = 97	n = 97
PPPM on day 7				
Total ≥ 6	46 (46%)	33 (34%)	32 (33%)	36 (37%)
Total ≥ 9	27 (27%)	24 (24%)	20 (21%)	26 (27%)
Whines and complains more than usual	47 (58%)	35 (45%)	36 (47%)	38 (57%)
Plays less than usual	43 (53%)	34 (44%)	37 (49%)	38 (57%)
Eats less than usual	66 (81%)	47 (61%)	47 (62%)	46 (68%)
Missing	18	20	21	30
Nausea days 1–3	27 (33%)	34 (43%)	36 (44%)	26 (33%)
Missing	16	18	15	18
Vomiting days 1–3	14 (17%)	21 (26%)	22 (27%)	19 (25%)
Missing	17	18	16	20
Any vomiting days 1–14	20 (26%)	25 (35%)	30 (39%)	26 (39%)
Missing	23	27	21	30
Simple analgesia dosing compliance*				
Days 1–3	47 (47%)	52 (53%)	46 (47%)	43 (44%)
Days 4–6	46 (46%)	41 (42%)	42 (43%)	37 (38%)
Days 7–9	15 (15%)	14 (14%)	19 (20%)	12 (12%)
Honey dosing compliance^†^				
Days 1–3	N/A	47 (48%)	42 (43%)	51 (53%)
Days 4–6	N/A	42 (43%)	42 (43%)	39 (40%)
Days 7–9	N/A	39 (40%)	33 (34%)	35 (36%)
Medical re‐presentation days 1–14	26 (26%)	27 (28%)	27 (28%)	25 (26%)
To primary care	13 (13%)	12 (12%)	11 (11%)	10 (10%)
To hospital	17 (17%)	20 (20%)	18 (19%)	16 (16%)
Re‐presentation for pain	10 (10%)	14 (14%)	12 (12%)	7 (7%)
For bleeding	13 (13%)	10 (10%)	8 (8%)	9 (9%)
For vomiting	0	5 (5%)	5 (5%)	3 (3%)
Other reasons	15 (15%)	15 (15%)	14 (14%)	13 (13%)
Re‐presentation to hospital > 1 day	5 (5%)	10 (10%)	7 (7%)	7 (7%)
Readmission to hospital days 1–14	9 (9%)	8 (8%)	6 (6%)	5 (5%)
For pain	0	1 (1%)	1 (1%)	0 (0%)
For bleeding	8 (8%)	5 (5%)	4 (4%)	4 (4%)
For vomiting	0	0	2 (2%)	0
Other reasons	1 (1%)	2 (2%)	0	1 (1%)

PPPM, Parents' Postoperative Pain Measure; a score of 6/14 was taken to equate to moderate pain and 9/14 to severe pain.

* Analgesia dosing compliance was defined as at least three paracetamol doses and at least two ibuprofen doses each day for the stated days.

^†^ Honey dosing compliance was defined as at least two honey doses each day for the stated days.

Parameter estimates for the final models for FPS‐R are available in online Supporting Information Appendix S2, Table S7. Mean pain improved significantly over time (by day 10 in the complete‐case analysis estimated ‐0.42 relative to day 1, p = 0.002; by day 9 in MICE ‐0.72, p < 0.001) with a small significant increase on day 3 (complete‐case analysis 0.62, p < 0.001; MICE 0.33, p = 0.074). The main effect estimates for pain in each treatment group, relative to standard care, were not statistically significant in the complete‐case analysis model (Marri 0.21, p = 0.417; placebo 0.41, p = 0.110; Manuka 0.02, p = 0.945). MICE modelling found the main effects of treatment group were statistically significant, but the estimated effect size did not reach clinical significance (Marri 0.52, p = 0.033; placebo 0.34, p = 0.165; Manuka 0.11, p = 0.648;).

Taking interaction with postoperative day into account, graphs of predicted pain show that on days 1–6, the placebo group had the highest mean pain score, but differences were within 1 point and did not represent clinical significance (online Supporting Information Appendix S4, Figure S3). From day 7 onwards, average pain between groups was not different. There was a statistically significant interaction between treatment group and condition of measurement in the complete‐case analysis model, however all differences were within 1 point, and this interaction was not statistically significant in the MICE model (online Supporting Information Appendix S4, Figure S3b) There were no significant differences in FPS‐R between honey and placebo groups at this frequency of ingestion (online Supporting Information Appendix S4, Figure S4). Overall, we found no evidence of an improved pain trajectory in the intervention groups over standard care.

**Figure 4:**
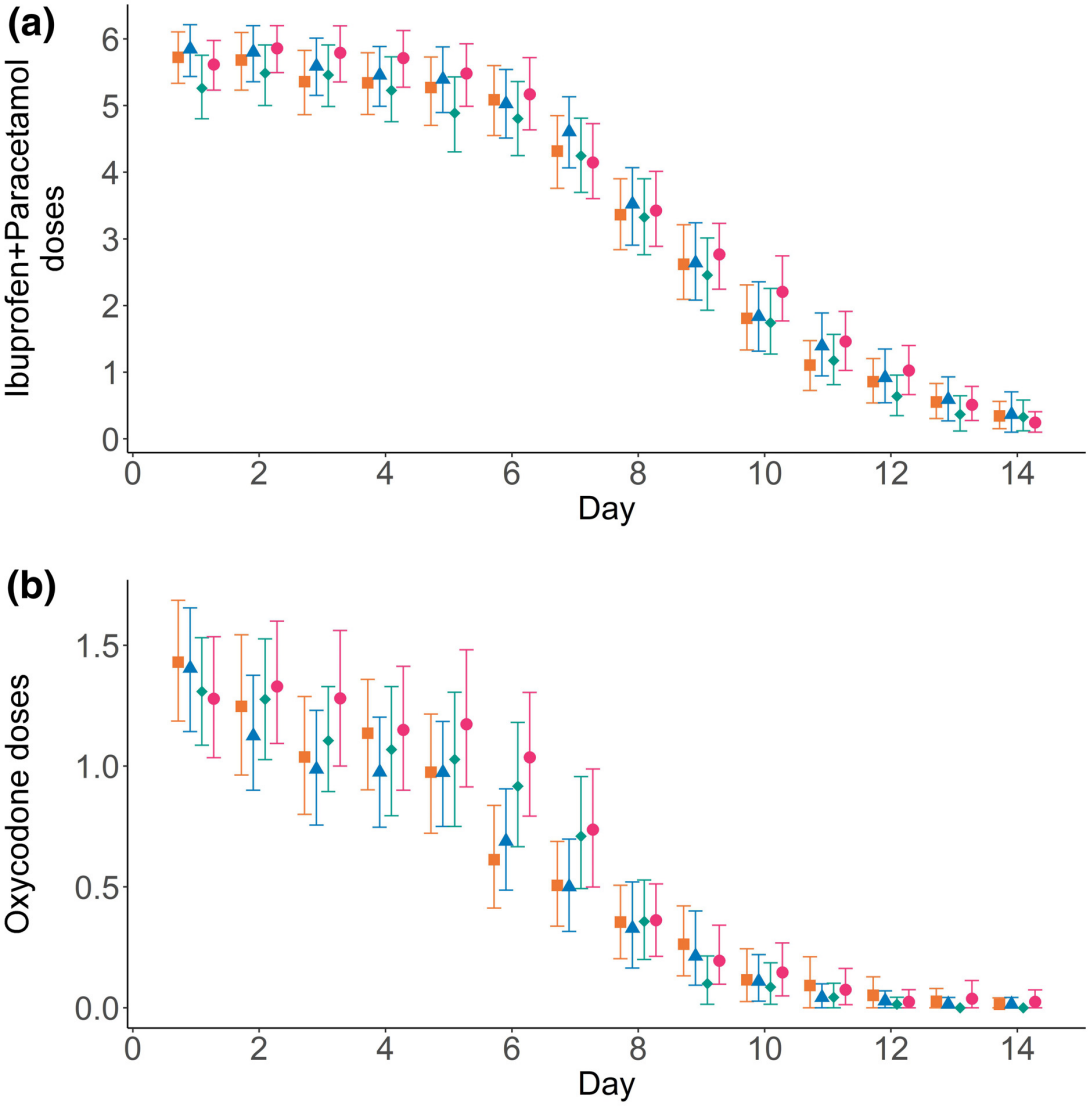
Number of analgesic doses per day for each treatment group. Data are mean and bootstrapped 95%CI. (a) Simple analgesia (ibuprofen and paracetamol) doses; (b) oxycodone doses. Orange squares, Manuka honey group; blue triangles, Marri honey group; green diamonds, placebo group; pink circles, standard care. Data are shown in online Supporting Information Appendix S4, Table S5.

Across groups, just under half of the children documented taking the recommended analgesic doses on a given day (Table 2). The number of doses of paracetamol and ibuprofen used were within half a dose across groups, across all days (Fig. 4a). As required oxycodone use decreased over the first week (Fig. 4b), with the standard care group taking about one more dose by day 7 and for 1 day more overall (online Supporting Information Appendix S4, Table S2). However, there was wide individual variation, and about 25–30% of children had dose data missing for some days. We deemed this unlikely to be a clinically significant reduction. On any given day, taking any analgesic medication was associated with higher pain scores across models: oxycodone (complete‐case analysis 0.45, p < 0.001; MICE 0.55, p < 0.001); ibuprofen (complete‐case analysis 0.46, p < 0.001; MICE 0.38, p < 0.001); and paracetamol (complete‐case analysis 0.33, p < 0.001; MICE 0.30, p < 0.001) (online Supporting Information Appendix S2, Table S7).

Across groups, 105 (27%) children re‐presented to hospital, primary care or both (Table 2), and this was similar between groups. Days 5 and 7 had the highest number of hospital re‐presentations (16 children each), followed by day 6 (14 children). Day 6 had the highest number re‐present to a primary care physician (13 children). The main reason for re‐presentation was pain (43 of 105 presentations, 41%), on its own or in combination with other symptoms, such as bleeding or feeding issues (Table 2). Hospital re‐presentation occurred with 72 children (18%) and 29 children (7%) were readmitted, with no clinically significant difference between groups. Honey groups had slightly more children presenting more than once to hospital and more with early vomiting, but less feeding issues on day 7. Across all groups, three children required further surgery for bleeding: two for primary haemorrhages on day 0 before any intervention and the other a secondary haemorrhage (day 11).

Table 3 summarises parent opinion and satisfaction surveys. The response rate was 168 (58%) for honey and placebo groups and 23 (23%) for standard care. Global satisfaction with study treatment was high (9/10) and similar across groups. Blinding was maintained. Of those who had honey, 54 (50%) believed it was helpful compared with 24 (38%) in the placebo group.

**Table 3:** Parent responses to follow‐up questionnaire. Values are mean (SD) or number (proportion).

	Standard care	Manuka honey	Marri honey	Placebo
	n = 99	n = 98	n = 97	n = 97
**Parent satisfaction score (0–10) with study treatment**				
Global satisfaction score	9.1 (1.7)	9.3 (1.8)	8.9 (2.0)	8.9 (1.7)
Missing	76	44	47	33
**The honey/placebo helped my child with their pain relief after surgery**				
Strongly disagree‐disagree	‐	7 (13%)	7 (14%)	14 (22%)
Neither agree nor disagree	‐	17 (32%)	18 (36%)	26 (41%)
Agree‐strongly agree	‐	29 (55%)	25 (50%)	24 (38%)
Missing	‐	45	47	33
**Do you think your child had**				
Honey	‐	25 (46%)	33 (65%)	22 (34%)
Placebo	‐	22 (41%)	11 (22%)	30 (47%)
Can't remember/unsure	‐	7 (13%)	7 (14%)	12 (19%)
Missing	‐	44	46	33
**If you think your child had honey, do you think they had**				
Manuka honey	‐	11 (44%)	17 (52%)	14 (64%)
Marri honey	‐	4 (16%)	4 (12%)	3 (14%)
Unsure	‐	10 (40%)	12 (36%)	5 (23%)
Missing	‐	73	64	75

## Discussion

This randomised controlled trial showed that honey, when added to our standardised analgesic regime for at least 7 days following extracapsular tonsillectomy in children, did not have a clinically significant impact on pain, recovery trajectory or medication use. This result was not altered by using MICE to allow for missing data. Parents rated highly all the analgesic regimes. In those taking honey, parents felt honey helped with pain relief and around 60% continued to use honey beyond the 7‐day study period.

Neither self‐reported nor parental reports of pain severity differed clinically between groups. Observed pain trajectories were in line with other studies, with a rise in pain scores around day 4 linked to re‐presentations [2, 5]. Post‐tonsillectomy pain is thought to be largely due to inflammation caused by injury to pharyngeal constrictor muscle fibres with peripheral sensitisation of exposed vagal and glossopharyngeal nerve fibres. This results in localised pain, painful swallow and, often, otalgia. The initial haemostatic phase injury response is compounded by additional immune response to oral commensal bacteria, resulting in a dense white ‘eschar’ [29]. The frequent second pain rise on days 4–7 correlates with peak inflammation, degree of neutrophil infiltration and the eschar sloughing off. Pain improves as the advancing epithelialisation re‐covers the tonsillar fossae [30].

Coblation and electrocautery tonsillectomy lead to thermal injury. In burn models, honey improves wound healing through a variety of mechanisms [31]. Different honeys promote healing in different ways. It is possible honeys with more antibacterial or antioxidant activity might have been more effective.

Honey‐treated burn dressings can promote healing [32]; however, applying honey regularly to the tonsillar fossa for prolonged periods is considerably harder. It may be that our honeys did not make sufficient prolonged contact with the wound. In previous paediatric tonsillectomy honey studies [13], simple by‐mouth instructions were used predominantly for application and the dosing frequency varied from hourly to eight hourly while awake; we chose up to six times per day. The two studies recommending hourly dosing did not report compliance, were short and showed no benefit [33, 34].

Surgical technique in tonsillectomy studies can also be a confounder. Previous honey studies used cold steel dissection predominantly; most did not state if electrocautery was used for haemostasis [13]. The reduced tissue damage of a cold technique allows quicker healing [29] and perhaps allowed honey to have a bigger impact in these studies.

Honey could also have a coating effect. Placebo had a comparable ingestion rate to the honeys and was similar to standard care for swallow discomfort, medication compliance and pain behaviours. However, the Parents' Postoperative Pain Measure item ‘*eating less than usual*’ was better in honey and placebo groups on several days. This may be evidence supporting a small demulcent effect. Any coating effect might have been diminished by low compliance, limited contact time due to swallowing, as well as the dilutional effects of saliva. Parents rated placebo less helpful for pain relief than honey on days 6 and 7 and the Parents' Postoperative Pain Measure item ‘*refusal to eat*’ was less in the honey groups. This may have come at the cost of increased post‐discharge nausea and vomiting and re‐presentations (Table 2 and online Supporting Information Appendix S4, Table S1). Though it did not lead to increased re‐presentations overall, increased early vomiting is a potential concern, as it may lead to dehydration and may increase bleeding risk [35].

Compliance with round‐the‐clock medications has long been an issue for children post‐tonsillectomy [36] and, despite its sweet taste, parents struggled to reach the requested daily doses of honey or placebo. Most children in the honey/placebo groups took at least two doses per day, and only 15–20% complied with the suggested dosing of six times a day over the 7 days (online Supporting Information Appendix S4, Figure S2). However, even in those with better compliance (at least two recorded doses every day), no difference was seen in the intention‐to‐treat analysis. The amount of honey required to show an effect is unknown and dosing frequency was highly variable in previous studies [13]. Two doses per day of honey may be insufficient to produce sufficient biological effect during the critical peak inflammatory days, when swallowing is most painful and children were less compliant. A higher number of doses taken may have produced a positive result, but in general these were motivated parents keen to use honey and children who had tolerated doses before discharge.

Honey has been used to improve the palatability of medicines for millennia [37] but did not improve medicine compliance. Across the groups, compliance with recommended regular simple analgesia was 48% in the initial days, falling to 42% by day 7 (Table 2). As part of routine care, all parents received pain management information from anaesthetists and counselling by the ward pharmacist and nursing staff. This was supported by written instructions and a medication diary. Nonetheless, underdosing/poor compliance with regular analgesia was common and was similar across groups and to previous audits [2]. Painful swallowing, bad‐tasting medicine and difficulties complying with a round‐the‐clock regime are common barriers reported [38]. Unsurprisingly, better‐tasting medicines are among the top 10 consumer peri‐operative research priorities [39]. Any potential sweet taste or demulcent effects of the honey did not translate into clinically significant differences in simple analgesic compliance (Table 2, Figure 4a). Statistical modelling suggested that children with higher pain scores received more medication with no evidence that being compliant with simple analgesia led to lower scores. However, we included each medication in the model as yes/no variables on a given day, so any possible non‐linearity was not considered; with a larger data set the association between number of doses and pain could be explored. Future studies may need to consider interventions that do not rely on compliance with regular swallowing.

Medical re‐presentations were common (26%) and peaked around day 6, an improvement on our previous cohort but more frequent than in some centres in the USA [40, 41]. In line with previous work, re‐presentations with pain (11%) remained the most common, suggesting the need for ongoing high‐quality research to tackle post‐discharge pain at home [42]. Only two of the 29 hospital readmissions were for pain only. Though the numbers are too small to be definitive, there was no signal of any increased infection or bleeding with re‐presentations (Table 2, online Supporting Information Appendix S4, Table S1).

There was significant dropout and missing data. Diaries were given in paper form and electronic questionnaires sent daily, with reminders if needed. Surveys were completed at home, and our research staff were unable to ensure consistent daily completion or validate medication use. Missing data are common in longitudinal studies, and restricting analysis to only complete cases is generally inappropriate [27]. Due to the daily questionnaires and longitudinal design of the study, only 29% of patients had complete data; this is comparable with a study of Parents' Postoperative Pain Measure in a similar cohort [42].

Previous studies of honey on post‐tonsillectomy pain do not discuss missing data [14], apart from some excluding incomplete cases. Our analysis reduced biases associated with survey non‐response, therefore contributing higher quality evidence. Measuring pain scores at rest and with eating twice daily over 14 days, while more onerous for parents, gave us the best examination of recovery trajectory. Post‐tonsillectomy pain is worst in the mornings, worse with swallowing and lasts about 10–14 days [8], and honey could have potentially altered any of these. Of the eight previous studies with children using honey and examining pain, only two used both resting and swallowing discomfort; four monitored for 5 days or less; only one had 14 days of data; and two used scores only every 3–4 days [14]. For example, reviewing pain in our cohort at day 7, without considering the days immediately before and after, one might erroneously conclude that honeys were significantly better according to parents (Fig. 3a).

A modified version of the Parents' Postoperative Pain Measure score was used, by omitting the 11th item which made little sense for tonsillectomies. Due to this amendment, the reference cut‐off values used (6/14 and 9/14 rather than 15) may be less valid and potentially under‐reported numbers with moderate and severe pain. In one study validating the Parents' Postoperative Pain Measure in children following tonsillectomy, items 11 and 15 were missing most frequently [42] and authors suggested dropping them in a reduced item score. In that study, for every point increase in the Parents' Postoperative Pain Measure total score the FPS‐R increased by about 0.4. In addition, due to high rates of missing data and high number of items, only total Parents' Postoperative Pain Measure scores were imputed and used. It is generally recommended that individual item scores be imputed [43]. Daily re‐presentations were not imputed and were likely under‐reported due to dropouts.

Sample size calculations assumed a mean clinically significant difference of 1 point in the FPS‐R [44]; thus, this study was underpowered to detect the actual differences in FPS‐R scores. This indicates a broader problem with assessing postoperative pain; the variation, even within treatment groups, is too large to detect small differences, unless the sample size is increased substantially. Previous studies in acute pain have suggested a minimum clinically significant difference of 2/10 for the FPS‐R scale [44]; thus, we concluded that there was no clinically important difference on any day. Parents and children were instructed on the FPS‐R scale as part of discharge education, but comprehension was not assessed. Additionally, for children aged < 6 y, parent reported pain scores were used [21].

The study was performed using a standardised surgical technique to achieve haemostasis, standardised post‐surgical analgesia and the honeys used were from Western Australia. These may limit the generalisability to those using different surgical techniques, honey or analgesia regimes but the study is larger than previous studies [13], was performed across three centres and the analgesic regime chosen is in line with international recommendations [15, 45]. We had a low response rate to our follow‐up questionnaire; these were sent out, in error, only at the end of the study, which may have affected recollection and compliance. All parents were asked about honey, which in the control group added to the low response rate.

In conclusion, this multicentre randomised controlled paediatric trial indicated that regular oral honey for 7 days post tonsillectomy, in addition to regular paracetamol, ibuprofen and as required oxycodone, did not significantly impact pain or recovery trajectory.

## Supporting information


**Appendix S1.** Sample of follow‐up diary.
**Appendix S2.** MICE and statistical modelling performed.
**Appendix S3.** Honey characterisation data.
**Appendix S4.** Additional data.
