# Supplementary material for: Impact of honey on post‐tonsillectomy pain in children (BEE PAIN FREE Trial): a multicentre, double‐blind, randomised controlled trial*
Source: Anaesthesia. 2025 May 5;80(8):946–58. doi: 10.1111/anae.16619 (PMC12256161; doi:10.1111/anae.16619)
Supplement: Supplementary file 1 — Appendix S1. Sample of follow‐up diary. Appendix S2. MICE and statistical modelling performed. Appendix S3. Honey characterisation data. Appendix S4. Additional data. [file ANAE-80-946-s001.docx]

# Appendix S1: Sample of Follow-up Diary

**Diary Booklet**

| 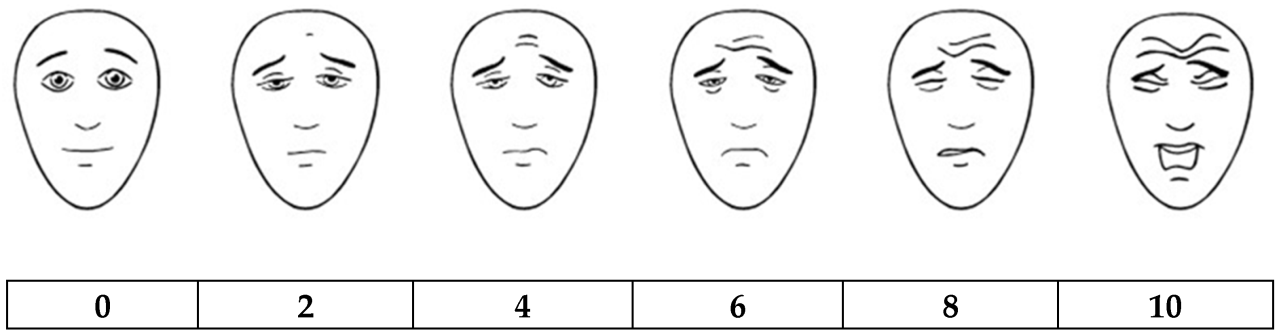Please show the face scale on this page to your child when asking him/her to rate their pain |
| --- |
|  |

For each day refer to the face scale which shows pain score numbers and enter the corresponding number in the table on each page

**Day 0 Day of Surgery (__/__/____)**

| **Honey administration -tick for each dose given** | | | | | | | | | | | | |
| --- | --- | --- | --- | --- | --- | --- | --- | --- | --- | --- | --- | --- |
| Breakfast | AM Snack | Lunch | | PM Snack | Dinner | | Night | | | Extra | | |
|  |  |  | |  |  | |  | | |  | | |
| 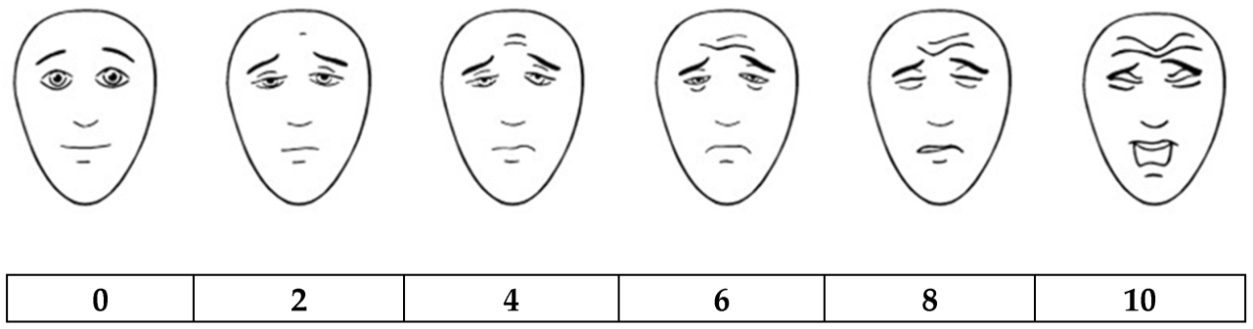 | | | | | | | | | | | | |
| **Check Point** | | | **Type of Pain** | | | **Pain Score** | | | **Comments** | | | |
| **Morning- Just before breakfast** | | | **Pain at rest** | | |  | | |  | | | |
| **Morning- During Breakfast** | | | **Pain on swallowing** | | |  | | |  | | | |
| **Evening- Just before evening meal** | | | **Pain at rest** | | |  | | |  | | | |
| **Evening - During evening meal** | | | **Pain on swallowing** | | |  | | |  | | | |
| **Did your child feel nauseous today?** | | | | | | | | | | | **Yes** | **No** |
| **If yes, how many episodes of nausea did they have?** | | | | | | | | | | | | |
| **Did your child vomit today?** | | | | | | | | | | | **Yes** | **No** |
| **If yes, how many vomiting episodes did they have** | | | | | | | | | | | | |
| **Did your child have non-study honey today?** | | | | | | | | | | | **Yes** | **No** |
| **If yes, please provide details on the non-study honey given and how many times they consumed the non-study honey today:** | | | | | | | | | | | | |
| **Did your child have any unplanned medical representations to the GP or hospital today?** | | | | | | | | **Yes** | **No** | | | |
| **If yes, please provide details:** | | | | | | | | | | | | |
| **Did your child have any other treatments/medications (prescriptions/over the counter) e.g. antibiotics, vitamins, lozenges, probiotics?** | | | | | | | | | | | | |

Parents were asked to complete this page on each day post-surgery from day 1 to day 14.

| **Honey administration -tick for each dose given** | | | | | | | | | | | | |
| --- | --- | --- | --- | --- | --- | --- | --- | --- | --- | --- | --- | --- |
| Breakfast | AM Snack | Lunch | | PM Snack | Dinner | | Night | | | Extra | | |
|  |  |  | |  |  | |  | | |  | | |
| 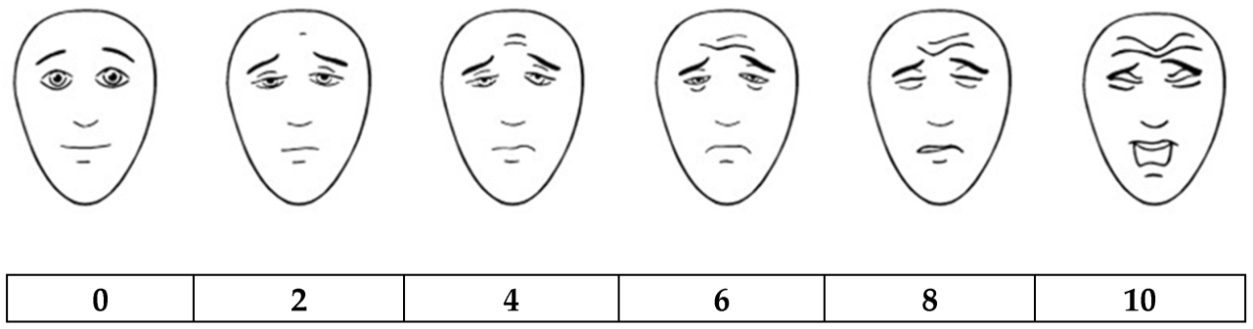 | | | | | | | | | | | | |
| **Check Point** | | | **Type of Pain** | | | **Pain Score** | | | **Comments** | | | |
| **Morning- Just before breakfast** | | | **Pain at rest** | | |  | | |  | | | |
| **Morning- During Breakfast** | | | **Pain on swallowing** | | |  | | |  | | | |
| **Evening- Just before evening meal** | | | **Pain at rest** | | |  | | |  | | | |
| **Evening - During evening meal** | | | **Pain on swallowing** | | |  | | |  | | | |
| **Did your child feel nauseous today?** | | | | | | | | | | | **Yes** | **No** |
| **If yes, how many episodes of nausea did they have?** | | | | | | | | | | | | |
| **Did your child vomit today?** | | | | | | | | | | | **Yes** | **No** |
| **If yes, how many vomiting episodes did they have** | | | | | | | | | | | | |
| **Did your child have non-study honey today?** | | | | | | | | | | | **Yes** | **No** |
| **If yes, please provide details on the non-study honey given and how many times they consumed the non-study honey today:** | | | | | | | | | | | | |
| **Did your child have any unplanned medical representations to the GP or hospital today?** | | | | | | | | **Yes** | **No** | | | |
| **If yes, please provide details:** | | | | | | | | | | | | |
| **Did your child have any other treatments/medications (prescriptions/over the counter) e.g. antibiotics, vitamins, lozenges, probiotics?** | | | | | | | | | | | | |

Parents were asked to complete this page for each day post-surgery from day 1 to day 14.

**Parents’ Postoperative Pain Measure (PPPM)**

**Please ✓ if answer is yes, or X if answer is no**

| From yesterday evening until tonight did your child… | Day 1 | Day 2 | … | Day 14 |
| --- | --- | --- | --- | --- |
| Whine or complain more than usual? |  |  | … |  |
| Cry more easily than usual? |  |  | … |  |
| Play less than usual? |  |  | … |  |
| Not do the things s/he normally does? |  |  | … |  |
| Act more worried than usual? |  |  | … |  |
| Act more quiet than usual? |  |  | … |  |
| Have less energy than usual? |  |  | … |  |
| Refuse to eat? |  |  | … |  |
| Eat less than usual? |  |  | … |  |
| Hold the sore part of his/her body? |  |  | … |  |
| Groan or moan more than usual? |  |  | … |  |
| Look more flushed than usual? |  |  | … |  |
| Want to be close to you more than usual? |  |  | … |  |
| Take medication when s/he normally refuses? |  |  | … |  |

# Appendix S2: MICE and Statistical Modelling Performed


## Statistical Methodology

All analysis was performed in the R statistical environment[1]. Statistical significance was taken at 5% (p=0.05) unless otherwise stated. Outcomes were based on intention-to-treat i.e., which group the child was randomised to, regardless of whether they took the honey/placebo on any given day.

### Characterising Missingness

To determine the suitability of this dataset for multiple imputation, missingness in variables of interest was explored using summary statistics and graphical methods. The proportion of missing data in both long (one row per patient per day) and wide (one row per patient over all days) formats was considered, and the missing data patterns and proportion of missing data over days were plotted. Patient demographic, pre-surgery, surgical characteristics, and pain measurements over days were compared between groups of patients with no missing data and at least one missing value in any of the relevant variables.

### Multiple Imputation by Chained Equations

Multiple imputation by chained equations (MICE) was performed using the mice package in R[2]. The data is longitudinal, with the primary outcome (Faces Pain Scale Revised, FPS-R) and some covariates (medications given, PPPM scores) collected repeatedly over 14 days post-surgery. The data was imputed in the wide format (one row per participant, with each daily measurement as a separate column), which has been shown to provide reliable estimates of regression coefficients[3]. This involves a large number of variables, which can result in failure of the imputation modelling process; for this reason the number of predictors for each variable is reduced by using a window of ±2 days for non-alike variables, and measurements from all days for alike variables. For example, for AM Swallow pain on day 7, all other AM swallow pain measures and only other measurements from days 5, 6, 7, 8, and 9 are used in the prediction model. For the same practical reasons, the PPPM score was imputed as a total score, rather than component-wise. All variables were imputed using the predictive mean matching (pmm) method in mice, the only exception being BMI, which was imputed using passive imputation within the imputation algorithm to preserve its relationship with weight and height while minimizing bias. To allow for interactions between the treatment group and other covariates in the analysis modelling, the data was split into its four treatment groups prior to imputation. After each data subset was multiply imputed, the data was recombined to run analysis models. This allows associations between other covariates and the outcome variable to differ according to the treatment group[4].

Multiple imputations were performed for m=5, 50, 100, 150, and 200 imputed datasets, with 20 iterations. Imputations were assessed by considering plots of the “real” data alongside imputed values for the m=5 datasets, and by considering distributions of the “real” data compared with distributions of imputed values.

### Analysis Models

To assess the primary outcome of FPS-R pain scores over the 14 days post-surgery, multivariable linear mixed models were fit, with FPS-R as the outcome and days since surgery, treatment group, time and condition (at rest or swallowing AM or PM) of measurement, patient characteristics, and daily PPPM scores and medications given as covariates. Interactions between treatment group with day, day with measurement condition, and treatment group with measurement condition were also considered. Random intercepts for subjects nested within sites were included to reflect the repeated-measures design. A complete-case analysis (CCA) model was fitted, along with models for each dataset within each set of imputations. For each model, stepwise variable selection was used based on the Akaike Information Criterion (AIC). To assess any changes in variables with increasing m, the proportion of models in which each variable was retained following stepwise selection was reported. Any variables that appeared in less than 50% of models for a given m were removed from the final model. The pooled model estimates for these models are reported. Any terms which were not statistically significantly related to the FPS-R score in these pooled models were tested using pooled Wald tests [4,5] and pooled estimates for these further reduced models are also reported.

## Results

### Characterizing Missingness

The measurements of pain in the morning upon swallowing (pain_am_swallow) have the highest proportion of missing data at 26.7% (Figure 1), followed by other variables measured daily including medications given, PPPM score, and other daily pain measurements. Most patient characteristic and surgical variables have no to low missing data, with exceptions being child-reported baseline pain before surgery (7.9% missing) and height (7.4% missing).


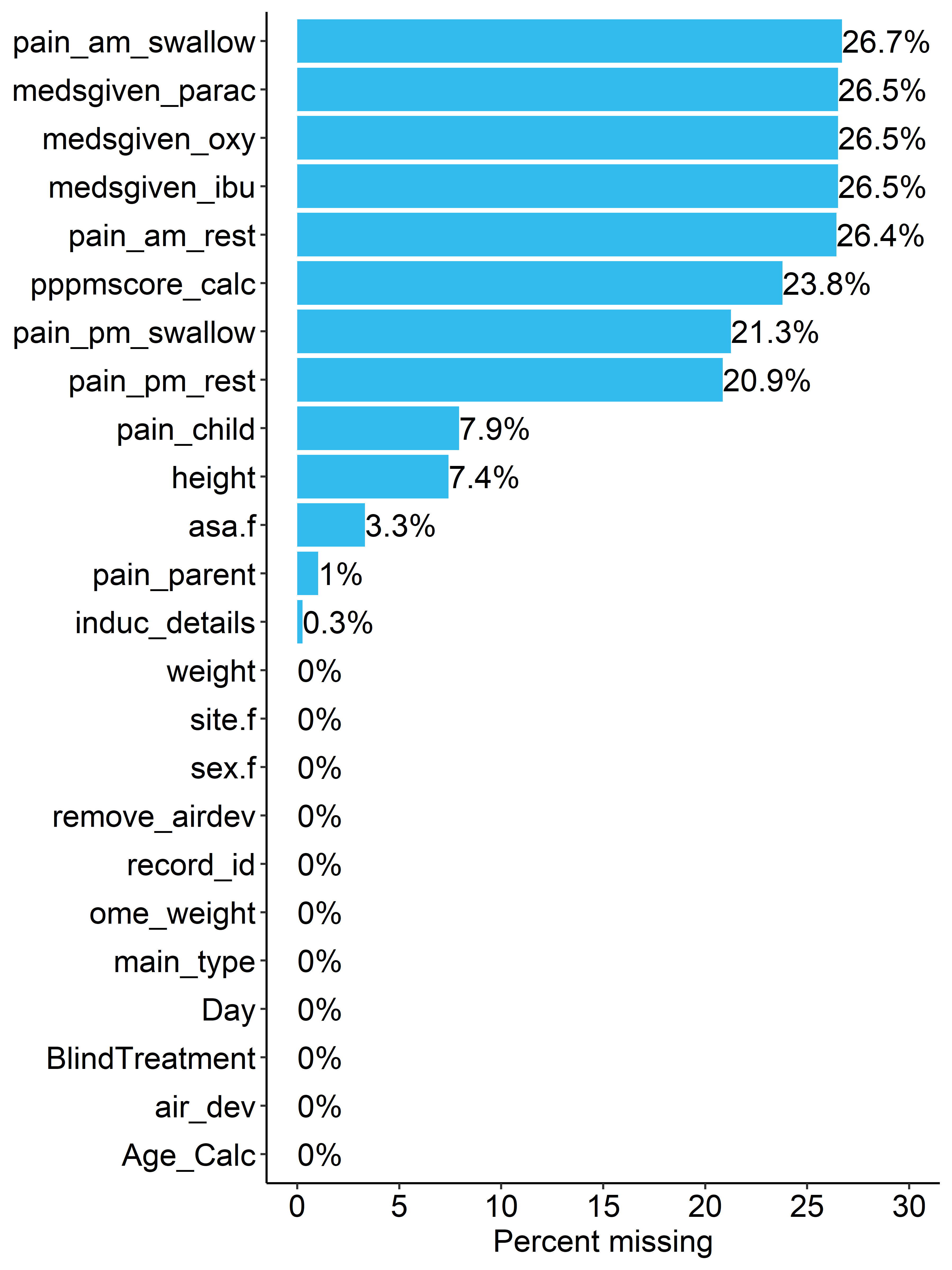


Figure S1:Percentage of missing data (in the long format in days and wide in circumstance of measurement) for each variable of interest).

The proportion of missing data increased with increasing days since surgery (Figure 2), from around 15% on Day 1 to around 25% on Day 14. On Day 0 (the day of surgery) there was a high volume of missing data in the pain measurements in the morning at rest and swallowing due to patients not having surgery until the afternoon. For a given day, the PPPM score consistently had the highest proportion of missingness.


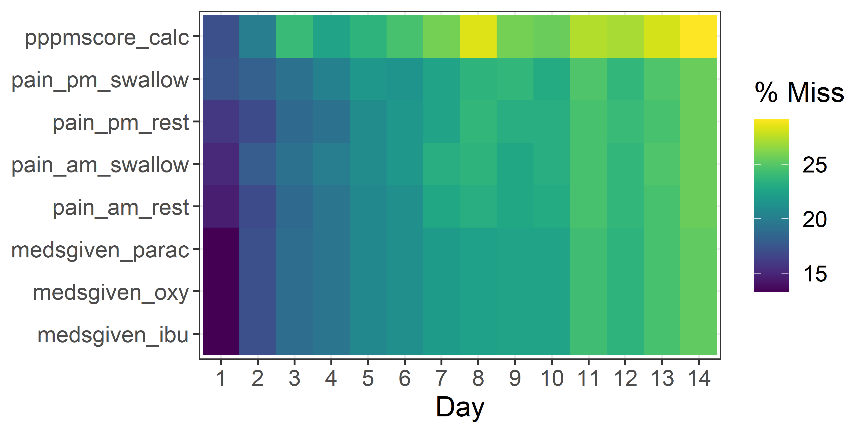


Figure S2: The proportion of missing data over days for variables measured repeatedly, where darkest blue indicates the lowest proportion of missing data and brightest yellow indicates the highest proportion of missing data. Day 0 is omitted.

Of the 391 participants, only 115 had no missing values in any of the variables considered in this analysis. From Table 1, there were differences in the age distribution of the patients with no missing and with some missing, with younger patients more represented in the group with some missing data. There were also slight differences in the distribution of patients between treatment groups, with a lower proportion of the Standard Care group patients having some missing values, and a higher proportion of the Marri Honey and Placebo group patients.

**Table S1 Summary statistics for some baseline and demographic variables between participants with no missing data and those with at least one missing data point in any variable at any timepoint.**

| Characteristic | Overall, N=391 | No missing, N=115 | Some missing, N=276 |
| --- | --- | --- | --- |
| Site | | | |
| FSH | 12 (3%) | 2 (2%) | 10 (4%) |
| PCH | 280 (72%) | 85 (74%) | 195 (71%) |
| SJOG | 99 (25%) | 28 (24%) | 71 (26%) |
| Sex | | | |
| Male | 221 (57%) | 67 (58%) | 154 (56%) |
| Female | 170 (43%) | 48 (42%) | 122 (44%) |
| Age | | | |
| 4 and under | 156 (40%) | 38 (33%) | 118 (43%) |
| 5-6.99 | 85 (22%) | 24 (21%) | 61 (22%) |
| 7-8.99 | 77 (20%) | 27 (23%) | 50 (18%) |
| 9 and over | 73 (19%) | 26 (23%) | 47 (17%) |
| Weight | 21.5 (17.4, 31.0) | 22.2 (18.1, 33.9) | 21.4 (17.2, 30.2) |
| Height | 119.8 ± 20.9 | 121.2 ± 21.3 | 119.1 ± 20.8 |
| Missing | 29 | 0 | 29 |
| BMI | 16.8 (15.4, 19.5) | 17.0 (15.4, 19.2) | 16.7 (15.4, 19.6) |
| Missing | 29 | 0 | 29 |
| Treatment | | | |
| Marri Honey | 97 (25%) | 25 (22%) | 72 (26%) |
| Placebo | 97 (25%) | 26 (23%) | 71 (26%) |
| Manuka Honey | 98 (25%) | 28 (24%) | 70 (25%) |
| Standard Care | 99 (25%) | 36 (31%) | 63 (23%) |
| ASA | | | |
| 1 | 164 (43%) | 50 (43%) | 114 (43%) |
| 2 | 211 (56%) | 65 (57%) | 146 (56%) |
| 3 | 3 (1%) | 0 (0%) | 3 (1%) |
| Missing | 13 | 0 | 13 |
| Induction details | | | |
| Inhalation | 338 (87%) | 102 (89%) | 236 (86%) |
| IV | 52 (13%) | 13 (11%) | 39 (14%) |
| Missing | 1 | 0 | 1 |
| Airway device | | | |
| LMA | 381 (97%) | 113 (98%) | 268 (97%) |
| ETT | 10 (3%) | 2 (2%) | 8 (3%) |
| Maintenance type | | | |
| Gas | 373 (95%) | 110 (96%) | 263 (95%) |
| TIVA | 18 (5%) | 5 (4%) | 13 (5%) |
| OME per kg | 0.4 (0.3, 0.5) | 0.4 (0.3, 0.5) | 0.4 (0.3, 0.5) |
| Removal of airway device | | | |
| Deep | 56 (14%) | 11 (10%) | 45 (16%) |
| Awake | 335 (86%) | 104 (90%) | 231 (84%) |
| Baseline pain (parent report) | | | |
| 0 | 369 (95%) | 111 (97%) | 258 (95%) |
| 1 | 3 (1%) | 2 (2%) | 1 (0%) |
| 2 | 4 (1%) | 0 (0%) | 4 (1%) |
| 3 | 3 (1%) | 0 (0%) | 3 (1%) |
| 4 | 1 (0%) | 1 (1%) | 0 (0%) |
| 5 | 2 (1%) | 0 (0%) | 2 (1%) |
| 6 | 4 (1%) | 1 (1%) | 3 (1%) |
| 8 | 1 (0%) | 0 (0%) | 1 (0%) |
| Missing | 4 | 0 | 4 |
| Baseline pain (child report) | | | |
| 0 | 341 (95%) | 109 (95%) | 232 (95%) |
| 1 | 2 (1%) | 1 (1%) | 1 (0%) |
| 2 | 4 (1%) | 1 (1%) | 3 (1%) |
| 3 | 4 (1%) | 1 (1%) | 3 (1%) |
| 5 | 2 (1%) | 1 (1%) | 1 (0%) |
| 6 | 3 (1%) | 1 (1%) | 2 (1%) |
| 8 | 2 (1%) | 1 (1%) | 1 (0%) |
| 10 | 2 (1%) | 0 (0%) | 2 (1%) |
| Missing | 31 | 0 | 31 |


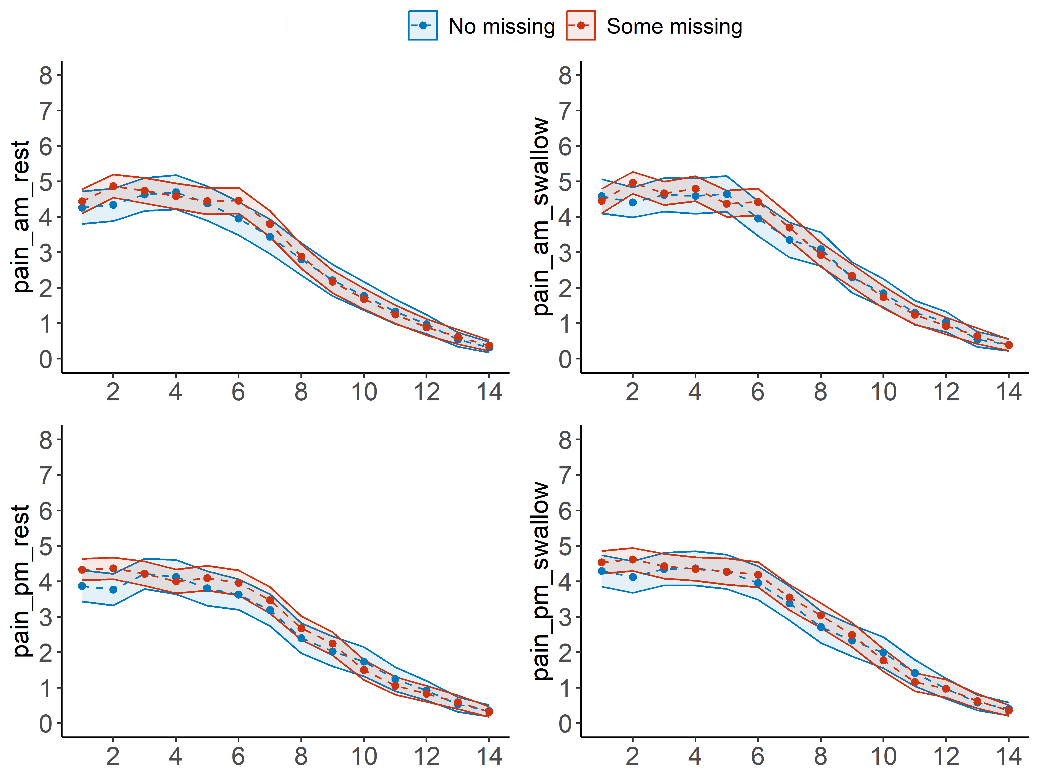


Figure S3 Mean and normal-approximated 95% CI FPS-R pain scores over postoperative days at each condition of measurement (panels) between the groups of participants with no missing data (blue) and some missing data (red).


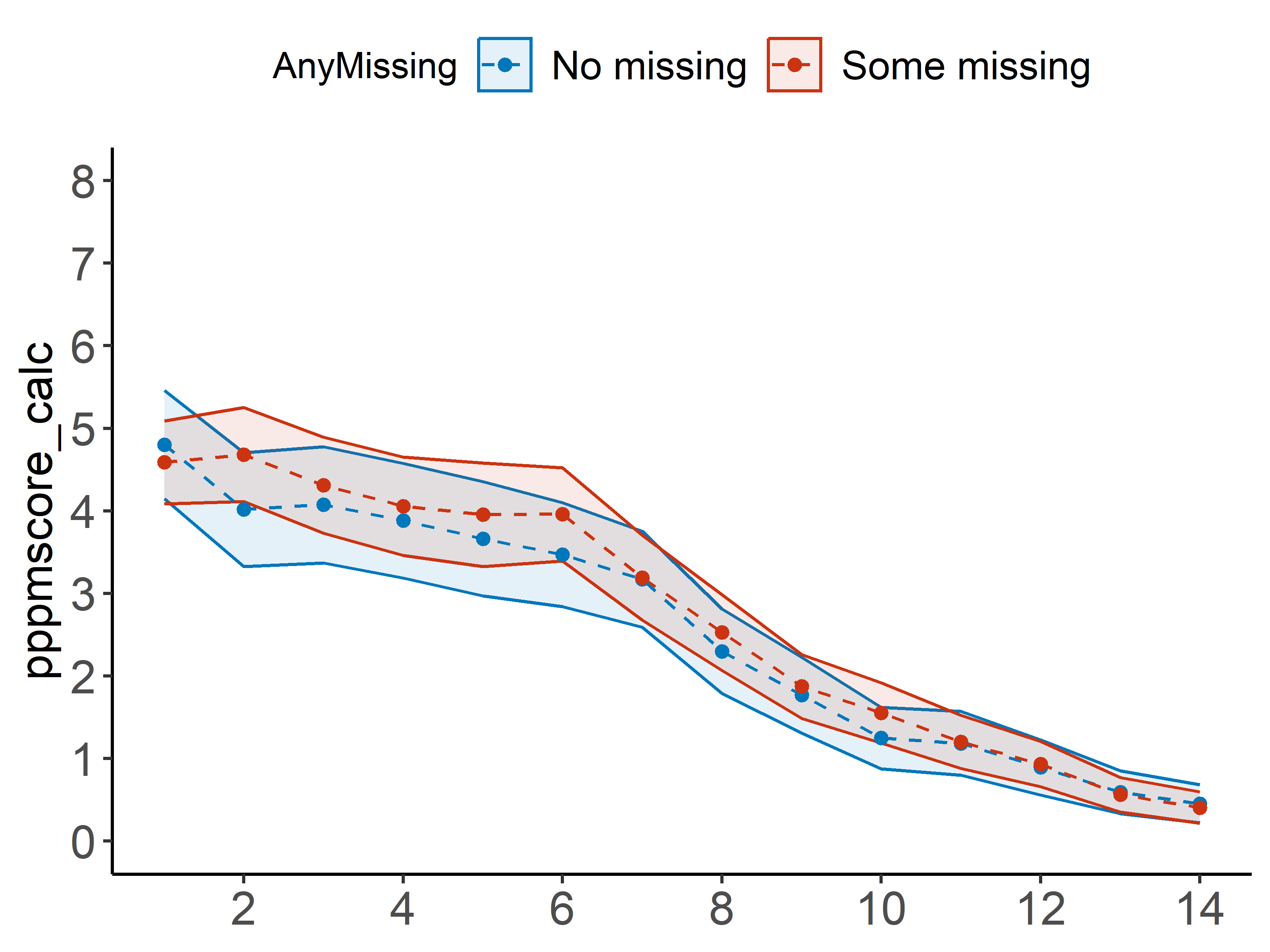


Figure S4 Mean and normal-approximated 95% CI PPPM pain scores over postoperative days at each condition of measurement (panels) between the groups of participants with no missing data (blue) and some missing data (red).

### Multiple Imputation by Chained Equations

A plot of the observed FPS-R pain scores at swallowing in the morning alongside the imputed values from the m=5 datasets for 25 randomly chosen participants (Figure 5) shows that imputed values generated were reasonable and could have been observed as real measurements. There were some differences in the distribution of observed and imputed data, particularly in the daily FPS-R measurements (Figure 6), with imputed data skewing more towards higher pain scores than observed data.


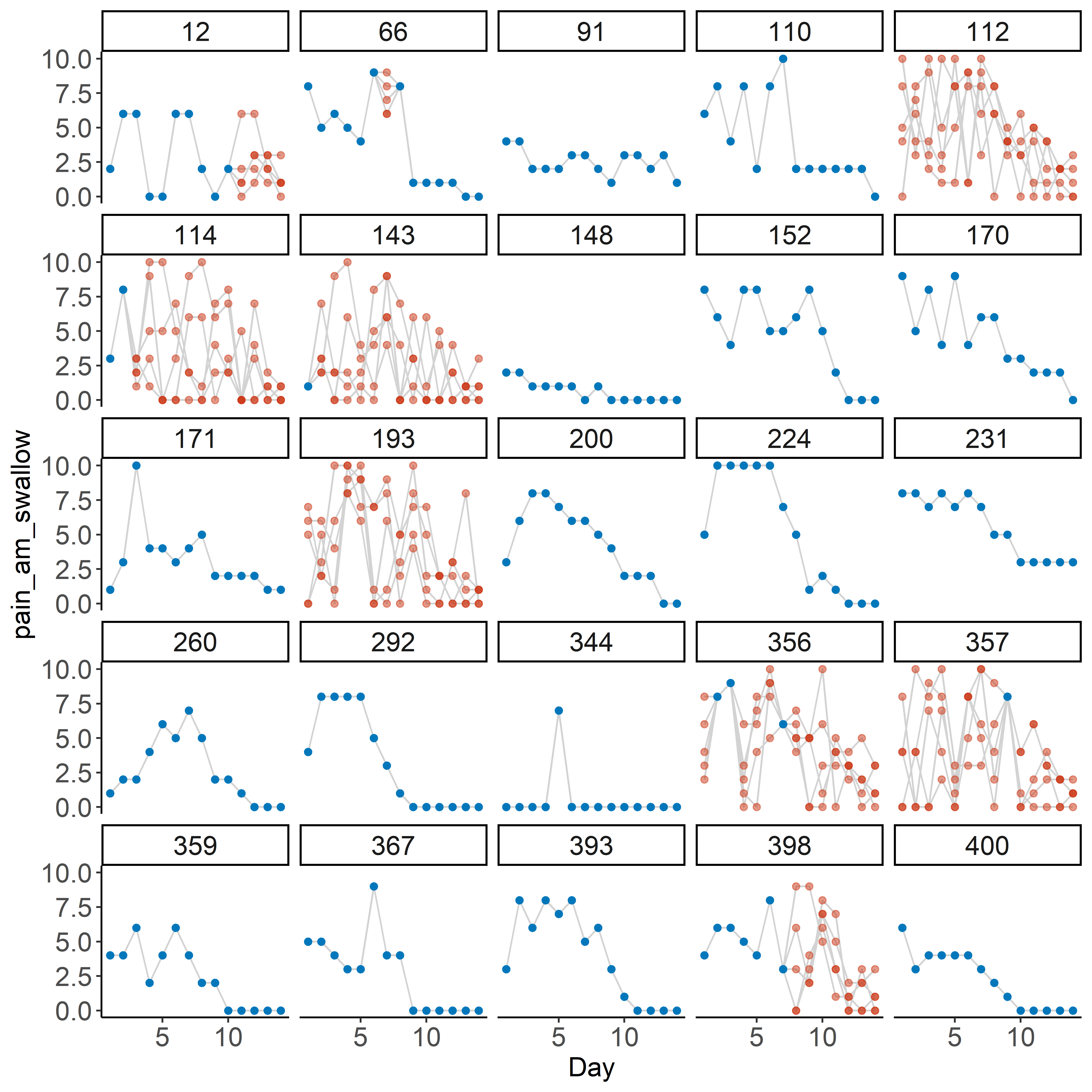


Figure S5: Real non-missing data (blue) and values from the m=5 imputed datasets (red) for FPS-R measurements at swallowing in the morning for 25 randomly chosen participants.


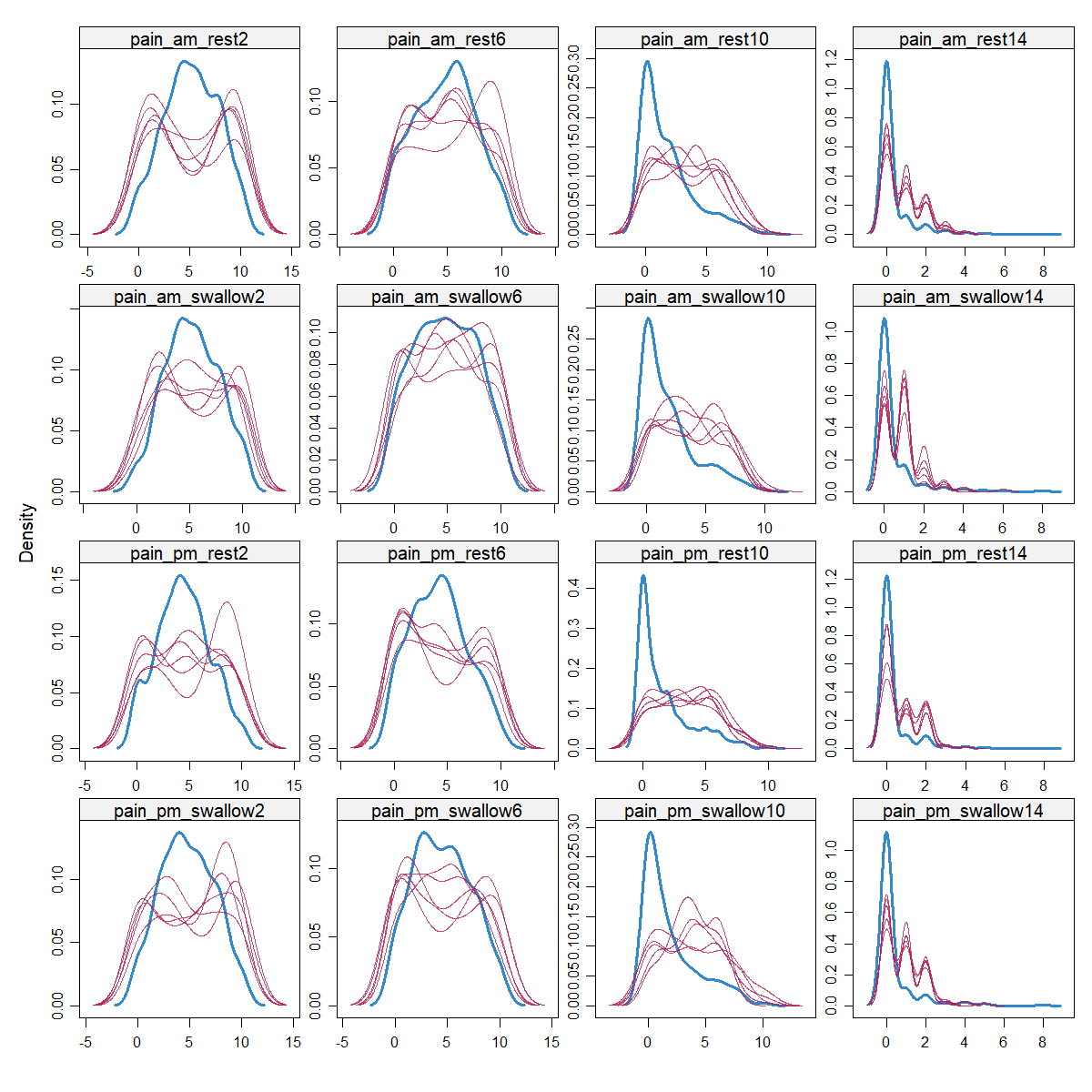


Figure S6: Distribution of observed data (blue) and imputed data (red) for the m=5 imputations for FPS-R measurements on selected days.

### Statistical Models

From Table S2, there are immediately clear differences in the variables retained for the multiply imputed datasets compared with the complete case analysis (CCA). From m=100 and higher, the proportion of models in which given variables are retained steadies, with little change between m=100, 150, and 200.

**Table S2: The proportion of models for each set of imputations which retain each candidate covariate following model selection based on AIC. CCA = complete-case analysis.**

| **Variables** | **CCA** | **m=5** | **m=50** | **m=100** | **m=150** | **m=200** |
| --- | --- | --- | --- | --- | --- | --- |
| Day | 1.00 | 1.00 | 1.00 | 1.00 | 1.00 | 1.00 |
| AMPMSwallowRest | 1.00 | 1.00 | 1.00 | 1.00 | 1.00 | 1.00 |
| Age.f | 1.00 | 1.00 | 1.00 | 1.00 | 1.00 | 1.00 |
| height | 1.00 | 1.00 | 1.00 | 1.00 | 1.00 | 1.00 |
| BLpain_pm_swallowBL | 1.00 | 1.00 | 1.00 | 1.00 | 1.00 | 1.00 |
| pppmscore_calc | 1.00 | 1.00 | 1.00 | 1.00 | 1.00 | 1.00 |
| medsgiven_oxy | 1.00 | 1.00 | 1.00 | 1.00 | 1.00 | 1.00 |
| medsgiven_ibu | 1.00 | 1.00 | 1.00 | 1.00 | 1.00 | 1.00 |
| medsgiven_parac | 1.00 | 1.00 | 1.00 | 1.00 | 1.00 | 1.00 |
| BlindTreatment | 1.00 | 1.00 | 0.96 | 0.99 | 0.99 | 0.99 |
| Day: BlindTreatment | 1.00 | 1.00 | 0.94 | 0.97 | 0.97 | 0.98 |
| pain_parent | 1.00 | 1.00 | 0.78 | 0.81 | 0.81 | 0.81 |
| Day: AMPMSwallowRest | 0.00 | 0.80 | 0.80 | 0.78 | 0.79 | 0.79 |
| ome_weight | 1.00 | 0.40 | 0.74 | 0.61 | 0.63 | 0.66 |
| AMPMSwallowRest: BlindTreatment | 1.00 | 0.40 | 0.58 | 0.60 | 0.57 | 0.61 |
| weight | 1.00 | 0.40 | 0.46 | 0.42 | 0.44 | 0.43 |
| BMI | 1.00 | 0.40 | 0.42 | 0.39 | 0.41 | 0.41 |
| BLpppmscore_calcBL | 1.00 | 0.80 | 0.50 | 0.43 | 0.40 | 0.37 |
| remove_airdev | 0.00 | 0.00 | 0.06 | 0.06 | 0.07 | 0.09 |
| pain_child | 1.00 | 0.00 | 0.04 | 0.02 | 0.02 | 0.02 |
| BLpain_pm_restBL | 0.00 | 0.00 | 0.00 | 0.00 | 0.00 | 0.00 |
| sex.f | 0.00 | 0.00 | 0.00 | 0.00 | 0.00 | 0.00 |
| asa.f | 0.00 | 0.00 | 0.00 | 0.00 | 0.00 | 0.00 |

After removing variables which appear in less than 50% of models for a given m following stepwise selection by AIC, the pooled model parameter estimates are shown in Table 3. Pooled parameter estimates change little from m=100 to m=200. Day since surgery, measurement time, age group, height, immediate post-surgery swallowing pain, and daily PPPM score, and medications given are all significantly associated with pain in the pooled models from m=100 and higher. Interaction terms are less clear-cut. The interaction between measurement time and treatment group is not significantly associated with daily pain scores in the pooled models, while only a few terms in the interaction between day and treatment group (specifically the Marri Honey and Placebo treatment groups) are statistically significant. Similarly, only a few terms in the interaction between day and measurement time are significantly different to zero.

We consider removing some of the interaction terms based on the results of pooled Wald tests (Table 4). The results indicate that none of the interaction terms should be retained; however, this includes the interaction between time and treatment, which appears in 99% of models for m>=100 following stepwise selection. Nevertheless, parameter estimates for m=100, 150, and 200 with these interaction terms omitted are shown in Table 5. The treatment group is no longer statistically significant in these models without interaction terms.

An alternative reduced model, where terms are removed by sequential Wald test with order based on the proportion of models in which they are retained (Table 6), is given in Table 7.

**Table S3 Model estimates for the complete case analysis (CCA) and for the pooled reduced models for the sets of imputed datasets with m=5,50,100,150 and 200. Variables which appear in 50% or more of the models for each given m are retained in the reduced models. Significance based on p-value is marked as . p<0.100, *p<0.050, **p<0.010, ***p<0.001. Reference levels are omitted for brevity.**

| **Variable** | **CCA** | | | **m=5** | | | **m=50** | | | **m=100** | | | **m=150** | | | **m=200** | | |
| --- | --- | --- | --- | --- | --- | --- | --- | --- | --- | --- | --- | --- | --- | --- | --- | --- | --- | --- |
|  | **coef** | **p** | **sig** | **coef** | **p** | **sig** | **coef** | **p** | **sig** | **coef** | **p** | **sig** | **coef** | **p** | **sig** | **coef** | **p** | **sig** |
| (Intercept) | -5.7 | 0.007 | ** | -0.18 | 0.798 |  | -0.53 | 0.44 |  | -0.64 | 0.344 |  | -0.64 | 0.342 |  | -0.64 | 0.345 |  |
| Day2 | 0.3 | 0.053 | . | 0.29 | 0.309 |  | 0.27 | 0.243 |  | 0.25 | 0.271 |  | 0.26 | 0.246 |  | 0.27 | 0.245 |  |
| Day3 | 0.6 | 0 | *** | 0.53 | 0.072 | . | 0.53 | 0.037 | * | 0.5 | 0.037 | * | 0.49 | 0.038 | * | 0.49 | 0.043 | * |
| Day4 | 0.4 | 0.002 | ** | 0.23 | 0.436 |  | 0.25 | 0.284 |  | 0.21 | 0.384 |  | 0.22 | 0.359 |  | 0.22 | 0.363 |  |
| Day5 | 0.5 | 0 | *** | 0.18 | 0.417 |  | 0.36 | 0.143 |  | 0.3 | 0.212 |  | 0.31 | 0.195 |  | 0.31 | 0.187 |  |
| Day6 | 0.4 | 0.007 | ** | 0.22 | 0.299 |  | 0.34 | 0.157 |  | 0.28 | 0.238 |  | 0.27 | 0.259 |  | 0.27 | 0.254 |  |
| Day7 | 0.1 | 0.614 |  | -0.17 | 0.467 |  | -0.18 | 0.471 |  | -0.19 | 0.422 |  | -0.2 | 0.393 |  | -0.2 | 0.402 |  |
| Day8 | 0.1 | 0.493 |  | -0.63 | 0.055 | . | -0.44 | 0.074 | . | -0.48 | 0.054 | . | -0.49 | 0.051 | . | -0.48 | 0.053 | . |
| Day9 | -0.2 | 0.235 |  | -0.89 | 0.007 | ** | -0.87 | 0.001 | ** | -0.86 | 0 | *** | -0.87 | 0 | *** | -0.87 | 0 | *** |
| Day10 | -0.4 | 0.002 | ** | -1.16 | 0.026 | * | -1.17 | 0 | *** | -1.21 | 0 | *** | -1.2 | 0 | *** | -1.19 | 0 | *** |
| Day11 | -0.6 | 0 | *** | -1.43 | 0 | *** | -1.47 | 0 | *** | -1.47 | 0 | *** | -1.47 | 0 | *** | -1.47 | 0 | *** |
| Day12 | -0.8 | 0 | *** | -1.61 | 0 | *** | -1.6 | 0 | *** | -1.65 | 0 | *** | -1.64 | 0 | *** | -1.64 | 0 | *** |
| Day13 | -0.9 | 0 | *** | -1.72 | 0 | *** | -1.74 | 0 | *** | -1.77 | 0 | *** | -1.77 | 0 | *** | -1.76 | 0 | *** |
| Day14 | -1.1 | 0 | *** | -2.02 | 0 | *** | -2.03 | 0 | *** | -2.08 | 0 | *** | -2.08 | 0 | *** | -2.07 | 0 | *** |
| AM Swallow | 0.2 | 0.023 | * | 0.09 | 0.561 |  | 0.12 | 0.5 |  | 0.1 | 0.557 |  | 0.1 | 0.555 |  | 0.11 | 0.538 |  |
| PM Rest | -0.3 | 0 | *** | -0.4 | 0.07 | . | -0.45 | 0.019 | * | -0.45 | 0.015 | * | -0.45 | 0.012 | * | -0.45 | 0.014 | * |
| PM Swallow | 0.1 | 0.394 |  | 0.07 | 0.712 |  | 0.1 | 0.581 |  | 0.1 | 0.627 |  | 0.1 | 0.608 |  | 0.1 | 0.607 |  |
| Marri Honey | 0.2 | 0.485 |  | 0.47 | 0.075 | . | 0.5 | 0.05 | . | 0.5 | 0.052 | . | 0.5 | 0.047 | * | 0.52 | 0.04 | * |
| Placebo | 0.4 | 0.123 |  | 0.26 | 0.36 |  | 0.35 | 0.189 |  | 0.34 | 0.19 |  | 0.35 | 0.184 |  | 0.36 | 0.173 |  |
| Manuka Honey | 0 | 0.929 |  | 0.06 | 0.815 |  | 0.25 | 0.304 |  | 0.2 | 0.412 |  | 0.21 | 0.403 |  | 0.21 | 0.389 |  |
| Age.f5-6.99 | -0.9 | 0 | *** | -0.66 | 0.001 | ** | -0.68 | 0 | *** | -0.69 | 0 | *** | -0.69 | 0 | *** | -0.69 | 0 | *** |
| Age.f7-8.99 | -1.5 | 0 | *** | -1.16 | 0 | *** | -1.17 | 0 | *** | -1.15 | 0 | *** | -1.15 | 0 | *** | -1.15 | 0 | *** |
| Age.f9 and over | -0.9 | 0.036 | * | -0.86 | 0.013 | * | -0.84 | 0.012 | * | -0.85 | 0.012 | * | -0.84 | 0.012 | * | -0.84 | 0.013 | * |
| weight | -0.1 | 0.03 | * |  |  |  |  |  |  |  |  |  |  |  |  |  |  |  |
| height | 0.1 | 0.001 | ** | 0.02 | 0 | *** | 0.02 | 0 | *** | 0.02 | 0 | *** | 0.02 | 0 | *** | 0.02 | 0 | *** |
| BMI | 0.1 | 0.052 | . |  |  |  |  |  |  |  |  |  |  |  |  |  |  |  |
| ome_weight | 1 | 0.133 |  |  |  |  | 0.55 | 0.139 |  | 0.5 | 0.172 |  | 0.5 | 0.166 |  | 0.51 | 0.159 |  |
| pain_parent | 0.3 | 0.005 | ** | 0.11 | 0.1 |  | 0.1 | 0.136 |  | 0.1 | 0.12 |  | 0.1 | 0.125 |  | 0.1 | 0.125 |  |
| pain_child | -0.1 | 0.14 |  |  |  |  |  |  |  |  |  |  |  |  |  |  |  |  |
| BLpain_pm_swallowBL | 0.2 | 0 | *** | 0.1 | 0 | *** | 0.1 | 0 | *** | 0.09 | 0 | *** | 0.09 | 0 | *** | 0.09 | 0 | *** |
| BLpppmscore_calcBL | -0.1 | 0.012 | * | -0.02 | 0.215 |  | -0.02 | 0.27 |  |  |  |  |  |  |  |  |  |  |
| pppmscore_calc | 0.3 | 0 | *** | 0.18 | 0 | *** | 0.18 | 0 | *** | 0.17 | 0 | *** | 0.17 | 0 | *** | 0.17 | 0 | *** |
| medsgiven_oxy | 0.4 | 0 | *** | 0.53 | 0 | *** | 0.56 | 0 | *** | 0.56 | 0 | *** | 0.55 | 0 | *** | 0.55 | 0 | *** |
| medsgiven_ibu | 0.5 | 0 | *** | 0.39 | 0 | *** | 0.38 | 0 | *** | 0.38 | 0 | *** | 0.38 | 0 | *** | 0.38 | 0 | *** |
| medsgiven_parac | 0.3 | 0 | *** | 0.33 | 0 | *** | 0.31 | 0 | *** | 0.31 | 0 | *** | 0.3 | 0 | *** | 0.3 | 0 | *** |
| Day2:AM Swallow |  |  |  | 0 | 0.995 |  | 0.02 | 0.918 |  | 0.04 | 0.857 |  | 0.04 | 0.872 |  | 0.03 | 0.882 |  |
| Day3:AM Swallow |  |  |  | -0.02 | 0.931 |  | -0.11 | 0.648 |  | -0.08 | 0.731 |  | -0.08 | 0.739 |  | -0.08 | 0.726 |  |
| Day4:AM Swallow |  |  |  | 0.03 | 0.918 |  | 0.05 | 0.826 |  | 0.09 | 0.706 |  | 0.08 | 0.731 |  | 0.07 | 0.76 |  |
| Day5:AM Swallow |  |  |  | -0.07 | 0.739 |  | -0.1 | 0.675 |  | -0.06 | 0.78 |  | -0.07 | 0.764 |  | -0.08 | 0.734 |  |
| Day6:AM Swallow |  |  |  | -0.08 | 0.742 |  | -0.03 | 0.877 |  | -0.03 | 0.895 |  | -0.02 | 0.917 |  | -0.03 | 0.908 |  |
| Day7:AM Swallow |  |  |  | -0.01 | 0.952 |  | -0.05 | 0.828 |  | -0.03 | 0.89 |  | -0.03 | 0.909 |  | -0.03 | 0.893 |  |
| Day8:AM Swallow |  |  |  | 0.1 | 0.718 |  | 0.04 | 0.859 |  | 0.05 | 0.843 |  | 0.06 | 0.802 |  | 0.06 | 0.815 |  |
| Day9:AM Swallow |  |  |  | 0.02 | 0.932 |  | 0.05 | 0.823 |  | 0.06 | 0.787 |  | 0.06 | 0.77 |  | 0.06 | 0.778 |  |
| Day10:AM Swallow |  |  |  | 0.04 | 0.849 |  | 0.02 | 0.936 |  | 0.04 | 0.854 |  | 0.04 | 0.851 |  | 0.03 | 0.879 |  |
| Day11:AM Swallow |  |  |  | -0.1 | 0.66 |  | -0.06 | 0.771 |  | -0.07 | 0.754 |  | -0.06 | 0.767 |  | -0.07 | 0.746 |  |
| Day12:AM Swallow |  |  |  | -0.06 | 0.805 |  | -0.01 | 0.948 |  | 0 | 0.998 |  | 0 | 0.998 |  | 0 | 0.984 |  |
| Day13:AM Swallow |  |  |  | -0.05 | 0.836 |  | -0.02 | 0.907 |  | -0.01 | 0.958 |  | -0.01 | 0.96 |  | -0.02 | 0.934 |  |
| Day14AM Swallow |  |  |  | -0.06 | 0.767 |  | -0.04 | 0.83 |  | -0.03 | 0.891 |  | -0.03 | 0.888 |  | -0.03 | 0.874 |  |
| Day2:PM Rest |  |  |  | -0.28 | 0.252 |  | -0.24 | 0.333 |  | -0.24 | 0.327 |  | -0.25 | 0.307 |  | -0.25 | 0.306 |  |
| Day3:PM Rest |  |  |  | -0.3 | 0.279 |  | -0.25 | 0.329 |  | -0.23 | 0.348 |  | -0.23 | 0.354 |  | -0.22 | 0.368 |  |
| Day4:PM Rest |  |  |  | -0.09 | 0.758 |  | -0.05 | 0.839 |  | -0.07 | 0.769 |  | -0.07 | 0.78 |  | -0.07 | 0.774 |  |
| Day5:PM Rest |  |  |  | -0.05 | 0.892 |  | -0.02 | 0.937 |  | 0 | 0.986 |  | 0 | 0.984 |  | -0.01 | 0.964 |  |
| Day6:PM Rest |  |  |  | -0.2 | 0.387 |  | -0.08 | 0.739 |  | -0.07 | 0.798 |  | -0.06 | 0.826 |  | -0.06 | 0.817 |  |
| Day7:PM Rest |  |  |  | 0.11 | 0.714 |  | 0.22 | 0.424 |  | 0.19 | 0.454 |  | 0.2 | 0.42 |  | 0.2 | 0.422 |  |
| Day8:PM Rest |  |  |  | 0.3 | 0.28 |  | 0.29 | 0.3 |  | 0.32 | 0.175 |  | 0.33 | 0.158 |  | 0.32 | 0.175 |  |
| Day9:PM Rest |  |  |  | 0.31 | 0.192 |  | 0.37 | 0.135 |  | 0.36 | 0.143 |  | 0.37 | 0.126 |  | 0.37 | 0.127 |  |
| Day10:PM Rest |  |  |  | 0.31 | 0.266 |  | 0.33 | 0.183 |  | 0.34 | 0.138 |  | 0.34 | 0.14 |  | 0.34 | 0.142 |  |
| Day11:PM Rest |  |  |  | 0.36 | 0.147 |  | 0.41 | 0.089 | . | 0.4 | 0.087 | . | 0.4 | 0.079 | . | 0.4 | 0.078 | . |
| Day12:PM Rest |  |  |  | 0.32 | 0.221 |  | 0.39 | 0.088 | . | 0.39 | 0.076 | . | 0.4 | 0.072 | . | 0.4 | 0.076 | . |
| Day13:PM Rest |  |  |  | 0.38 | 0.128 |  | 0.45 | 0.045 | * | 0.44 | 0.044 | * | 0.44 | 0.042 | * | 0.44 | 0.044 | * |
| Day14:PM Rest |  |  |  | 0.37 | 0.108 |  | 0.4 | 0.07 | . | 0.41 | 0.057 | . | 0.42 | 0.051 | . | 0.42 | 0.053 | . |
| Day2:PM Swallow |  |  |  | -0.36 | 0.1 |  | -0.36 | 0.143 |  | -0.34 | 0.166 |  | -0.35 | 0.153 |  | -0.34 | 0.163 |  |
| Day3:PM Swallow |  |  |  | -0.33 | 0.141 |  | -0.39 | 0.135 |  | -0.34 | 0.171 |  | -0.35 | 0.166 |  | -0.35 | 0.169 |  |
| Day4:PM Swallow |  |  |  | -0.15 | 0.634 |  | -0.17 | 0.466 |  | -0.13 | 0.604 |  | -0.14 | 0.573 |  | -0.15 | 0.556 |  |
| Day5:PM Swallow |  |  |  | -0.17 | 0.55 |  | -0.22 | 0.396 |  | -0.18 | 0.465 |  | -0.18 | 0.455 |  | -0.19 | 0.428 |  |
| Day6:PM Swallow |  |  |  | -0.34 | 0.101 |  | -0.27 | 0.291 |  | -0.24 | 0.361 |  | -0.24 | 0.363 |  | -0.24 | 0.362 |  |
| Day7:PM Swallow |  |  |  | -0.06 | 0.821 |  | -0.04 | 0.894 |  | -0.03 | 0.919 |  | -0.02 | 0.944 |  | -0.02 | 0.944 |  |
| Day8:PM Swallow |  |  |  | 0.24 | 0.442 |  | 0.13 | 0.619 |  | 0.14 | 0.599 |  | 0.16 | 0.554 |  | 0.15 | 0.572 |  |
| Day9:PM Swallow |  |  |  | 0.12 | 0.647 |  | 0.17 | 0.485 |  | 0.16 | 0.528 |  | 0.17 | 0.502 |  | 0.17 | 0.501 |  |
| Day10:PM Swallow |  |  |  | 0.15 | 0.565 |  | 0.16 | 0.527 |  | 0.17 | 0.472 |  | 0.17 | 0.477 |  | 0.17 | 0.479 |  |
| Day11:PM Swallow |  |  |  | 0 | 0.988 |  | 0.1 | 0.694 |  | 0.08 | 0.741 |  | 0.08 | 0.732 |  | 0.09 | 0.713 |  |
| Day12:PM Swallow |  |  |  | -0.04 | 0.869 |  | 0.04 | 0.866 |  | 0.04 | 0.865 |  | 0.04 | 0.859 |  | 0.04 | 0.851 |  |
| Day13:PM Swallow |  |  |  | -0.06 | 0.792 |  | 0 | 0.995 |  | -0.01 | 0.98 |  | 0 | 0.994 |  | 0 | 0.993 |  |
| Day14:PM Swallow |  |  |  | -0.04 | 0.86 |  | -0.03 | 0.894 |  | 0 | 0.987 |  | 0 | 0.988 |  | 0 | 1 |  |
| Day2: Marri Honey | 0 | 0.792 |  | -0.1 | 0.809 |  | -0.05 | 0.85 |  | -0.05 | 0.865 |  | -0.06 | 0.835 |  | -0.07 | 0.8 |  |
| Day3: Marri Honey | -0.5 | 0.014 | * | -0.39 | 0.26 |  | -0.24 | 0.42 |  | -0.26 | 0.326 |  | -0.25 | 0.347 |  | -0.26 | 0.337 |  |
| Day4: Marri Honey | 0.1 | 0.775 |  | -0.03 | 0.902 |  | 0.02 | 0.93 |  | 0.03 | 0.928 |  | 0.03 | 0.924 |  | 0.02 | 0.93 |  |
| Day5: Marri Honey | -0.2 | 0.311 |  | -0.18 | 0.368 |  | -0.32 | 0.277 |  | -0.27 | 0.334 |  | -0.27 | 0.329 |  | -0.28 | 0.318 |  |
| Day6: Marri Honey | -0.2 | 0.412 |  | -0.08 | 0.813 |  | -0.24 | 0.4 |  | -0.19 | 0.493 |  | -0.19 | 0.51 |  | -0.19 | 0.502 |  |
| Day7: Marri Honey | 0 | 0.942 |  | -0.29 | 0.355 |  | -0.31 | 0.275 |  | -0.27 | 0.314 |  | -0.27 | 0.322 |  | -0.28 | 0.314 |  |
| Day8: Marri Honey | -0.2 | 0.208 |  | -0.28 | 0.386 |  | -0.33 | 0.27 |  | -0.31 | 0.295 |  | -0.31 | 0.285 |  | -0.32 | 0.272 |  |
| Day9: Marri Honey | -0.3 | 0.083 | . | -0.27 | 0.358 |  | -0.3 | 0.284 |  | -0.33 | 0.232 |  | -0.32 | 0.221 |  | -0.33 | 0.217 |  |
| Day10: Marri Honey | -0.2 | 0.326 |  | -0.2 | 0.592 |  | -0.15 | 0.599 |  | -0.13 | 0.645 |  | -0.15 | 0.587 |  | -0.17 | 0.554 |  |
| Day11: Marri Honey | -0.4 | 0.035 | * | -0.3 | 0.239 |  | -0.31 | 0.258 |  | -0.3 | 0.242 |  | -0.29 | 0.252 |  | -0.31 | 0.24 |  |
| Day12: Marri Honey | -0.4 | 0.06 | . | -0.39 | 0.195 |  | -0.41 | 0.106 |  | -0.39 | 0.119 |  | -0.4 | 0.114 |  | -0.41 | 0.101 |  |
| Day13: Marri Honey | -0.5 | 0.015 | * | -0.55 | 0.053 | . | -0.54 | 0.022 | * | -0.53 | 0.024 | * | -0.53 | 0.027 | * | -0.55 | 0.023 | * |
| Day14: Marri Honey | -0.3 | 0.096 | . | -0.49 | 0.046 | * | -0.49 | 0.041 | * | -0.47 | 0.047 | * | -0.47 | 0.045 | * | -0.48 | 0.041 | * |
| Day2: Placebo | 0 | 0.792 |  | 0.19 | 0.68 |  | 0.11 | 0.696 |  | 0.12 | 0.671 |  | 0.11 | 0.707 |  | 0.1 | 0.737 |  |
| Day3: Placebo | 0 | 0.897 |  | 0.12 | 0.771 |  | 0.08 | 0.787 |  | 0.08 | 0.772 |  | 0.08 | 0.769 |  | 0.09 | 0.753 |  |
| Day4: Placebo | 0.2 | 0.27 |  | 0.36 | 0.205 |  | 0.3 | 0.309 |  | 0.27 | 0.365 |  | 0.27 | 0.352 |  | 0.28 | 0.325 |  |
| Day5: Placebo | 0.2 | 0.231 |  | 0.62 | 0.008 | ** | 0.36 | 0.261 |  | 0.39 | 0.188 |  | 0.38 | 0.203 |  | 0.38 | 0.215 |  |
| Day6: Placebo | 0.2 | 0.411 |  | 0.34 | 0.294 |  | 0.13 | 0.651 |  | 0.17 | 0.54 |  | 0.17 | 0.538 |  | 0.17 | 0.559 |  |
| Day7: Placebo | -0.2 | 0.432 |  | 0.18 | 0.566 |  | 0.04 | 0.899 |  | 0.07 | 0.826 |  | 0.07 | 0.818 |  | 0.06 | 0.842 |  |
| Day8: Placebo | -0.3 | 0.115 |  | -0.06 | 0.803 |  | -0.17 | 0.566 |  | -0.17 | 0.56 |  | -0.18 | 0.54 |  | -0.18 | 0.533 |  |
| Day9: Placebo | -0.4 | 0.024 | * | -0.26 | 0.656 |  | -0.27 | 0.335 |  | -0.28 | 0.366 |  | -0.29 | 0.337 |  | -0.29 | 0.319 |  |
| Day10: Placebo | -0.2 | 0.343 |  | -0.01 | 0.981 |  | -0.13 | 0.658 |  | -0.11 | 0.69 |  | -0.13 | 0.64 |  | -0.13 | 0.628 |  |
| Day11: Placebo | -0.3 | 0.157 |  | -0.07 | 0.82 |  | -0.14 | 0.649 |  | -0.17 | 0.52 |  | -0.19 | 0.488 |  | -0.19 | 0.474 |  |
| Day12: Placebo | -0.3 | 0.158 |  | -0.17 | 0.492 |  | -0.23 | 0.369 |  | -0.22 | 0.379 |  | -0.24 | 0.36 |  | -0.24 | 0.34 |  |
| Day13: Placebo | -0.6 | 0.004 | ** | -0.45 | 0.18 |  | -053 | 0.029 | * | -0.54 | 0.032 | * | -0.54 | 0.032 | * | -0.54 | 0.03 | * |
| Day14: Placebo | -0.4 | 0.031 | * | -0.25 | 0.423 |  | -0.3 | 0.224 |  | -0.29 | 0.251 |  | -0.3 | 0.229 |  | -0.3 | 0.217 |  |
| Day2: Manuka Honey | -0.2 | 0.261 |  | 0.03 | 0.902 |  | -0.02 | 0.937 |  | 0.02 | 0.919 |  | 0.02 | 0.947 |  | 0.02 | 0.946 |  |
| Day3: Manuka Honey | 0.2 | 0.329 |  | 0.24 | 0.328 |  | 0.2 | 0.469 |  | 0.22 | 0.436 |  | 0.23 | 0.396 |  | 0.22 | 0.397 |  |
| Day4: Manuka Honey | 0 | 0.867 |  | 0.17 | 0.6 |  | 0.11 | 0.691 |  | 0.11 | 0.681 |  | 0.1 | 0.684 |  | 0.11 | 0.673 |  |
| Day5: Manuka Honey | 0 | 0.91 |  | 0.17 | 0.573 |  | 0.03 | 0.927 |  | 0.06 | 0.799 |  | 0.05 | 0.851 |  | 0.05 | 0.855 |  |
| Day6: Manuka Honey | -0.3 | 0.127 |  | 0.02 | 0.94 |  | -0.26 | 0.354 |  | -0.15 | 0.556 |  | -0.15 | 0.562 |  | -0.16 | 0.541 |  |
| Day7: Manuka Honey | 0.4 | 0.057 | . | 0.24 | 0.452 |  | 0.14 | 0.598 |  | 0.22 | 0.414 |  | 0.22 | 0.401 |  | 0.21 | 0.437 |  |
| Day8: Manuka Honey | -0.3 | 0.171 |  | 0.01 | 0.981 |  | -0.16 | 0.571 |  | -0.12 | 0.681 |  | -0.12 | 0.67 |  | -0.13 | 0.642 |  |
| Day9: Manuka Honey | -0.1 | 0.701 |  | 0.06 | 0.782 |  | -0.02 | 0.937 |  | -0.01 | 0.968 |  | -0.01 | 0.973 |  | -0.01 | 0.961 |  |
| Day10: Manuka Honey | 0 | 0.991 |  | 0.03 | 0.926 |  | -0.03 | 0.892 |  | -0.03 | 0.924 |  | -0.03 | 0.903 |  | -0.04 | 0.868 |  |
| Day11: Manuka Honey | 0.1 | 0.632 |  | 0.05 | 0.876 |  | -0.04 | 0.872 |  | -0.03 | 0.894 |  | -0.04 | 0.871 |  | -0.05 | 0.854 |  |
| Day12: Manuka Honey | 0 | 0.973 |  | -0.13 | 0.644 |  | -0.2 | 0.379 |  | -0.15 | 0.525 |  | -0.16 | 0.494 |  | -0.17 | 0.481 |  |
| Day13: Manuka Honey | -0.1 | 0.747 |  | -0.35 | 0.11 |  | -0.39 | 0.096 | . | -0.36 | 0.111 |  | -0.37 | 0.112 |  | -0.37 | 0.107 |  |
| Day14: Manuka Honey | 0.1 | 0.636 |  | -0.15 | 0.477 |  | -0.22 | 0.342 |  | -0.18 | 0.415 |  | -0.19 | 0.406 |  | -0.2 | 0.387 |  |
| AM Swallow: Marri Honey | -0.3 | 0.011 | * |  |  |  | -0.11 | 0.352 |  | -0.12 | 0.37 |  | -0.12 | 0.373 |  | -0.13 | 0.345 |  |
| PM Rest: Marri Honey | 0.1 | 0.245 |  |  |  |  | 0.14 | 0.25 |  | 0.13 | 0.313 |  | 0.13 | 0.339 |  | 0.13 | 0.341 |  |
| PM Swallow: Marri Honey | -0.2 | 0.1 |  |  |  |  | -0.04 | 0.736 |  | -0.07 | 0.655 |  | -0.08 | 0.604 |  | -0.08 | 0.57 |  |
| AM Swallow: Placebo | 0 | 0.883 |  |  |  |  | 0.02 | 0.87 |  | 0.01 | 0.963 |  | 0.01 | 0.943 |  | 0.01 | 0.969 |  |
| PM Rest: Placebo | -0.1 | 0.624 |  |  |  |  | 0 | 0.977 |  | -0.03 | 0.839 |  | -0.02 | 0.866 |  | -0.02 | 0.898 |  |
| PM Swallow: Placebo | -0.1 | 0.173 |  |  |  |  | -0.05 | 0.737 |  | -0.07 | 0.636 |  | -0.07 | 0.631 |  | -0.08 | 0.602 |  |
| AM Swallow: Manuka Honey | -0.1 | 0.457 |  |  |  |  | -0.11 | 0.417 |  | -0.1 | 0.414 |  | -0.1 | 0.412 |  | -0.1 | 0.417 |  |
| PM Rest:Manuka Honey | 0 | 0.651 |  |  |  |  | -0.13 | 0.32 |  | -0.12 | 0.322 |  | -0.12 | 0.333 |  | -0.12 | 0.356 |  |
| PM Swallow:Manuka Honey | 0 | 0.662 |  |  |  |  | -0.17 | 0.214 |  | -0.17 | 0.22 |  | -0.17 | 0.221 |  | -0.17 | 0.234 |  |

**Table S4: Results of pooled Wald tests for interaction terms in the m=100, 150 and 200 models. Terms were tested sequentially moving down the column (i.e., if a term was not retained, it was removed from the model before testing the next term).**

| **Interaction** | **m=100** | | **m=150** | | **m=200** | |
| --- | --- | --- | --- | --- | --- | --- |
|  | **p-value** | **Retain** | **p-value** | **Retain** | **p-value** | **Retain** |
| AMPMSwallowRest:BlindTreatment | 0.517 | NO | 0.535 | NO | 0.549 | NO |
| Day:AMPMSwallowRest | 0.167 | NO | 0.149 | NO | 0.147 | NO |
| Day:BlindTreatment | 0.745 | NO | 0.718 | NO | 0.717 | NO |

**Table S5: Models with interaction terms omitted based on the results of pooled Wald tests. Model estimates for the complete case analysis (CCA) and for the pooled reduced models for the sets of imputed datasets with m=100,150 and 200. Variables which appear in 50% or more of the models for each given m are retained in the reduced models. Significance based on p-value is marked as . p<0.100, *p<0.050, **p<0.010, ***p<0.001. Reference levels are omitted for brevity.**

| **Variable** | **CCA** | | | **m=100** | | | **m=150** | | | **m=200** | | |
| --- | --- | --- | --- | --- | --- | --- | --- | --- | --- | --- | --- | --- |
|  | **coef** | **p** | **sig** | **coef** | **p** | **sig** | **coef** | **p** | **sig** | **coef** | **p** | **sig** |
| (Intercept) | -5.5 | 0.008 | ** | -0.54 | 0.413 |  | -0.54 | 0.412 |  | -0.53 | 0.42 |  |
| Day2 | 0.2 | 0.003 | ** | 0.14 | 0.155 |  | 0.14 | 0.154 |  | 0.14 | 0.155 |  |
| Day3 | 0.6 | 0 | *** | 0.34 | 0.001 | ** | 0.35 | 0.001 | ** | 0.34 | 0.001 | ** |
| Day4 | 0.5 | 0 | *** | 0.28 | 0.006 | ** | 0.28 | 0.005 | ** | 0.29 | 0.005 | ** |
| Day5 | 0.5 | 0 | *** | 0.28 | 0.009 | ** | 0.28 | 0.007 | ** | 0.27 | 0.009 | ** |
| Day6 | 0.3 | 0 | *** | 0.15 | 0.123 |  | 0.15 | 0.143 |  | 0.15 | 0.161 |  |
| Day7 | 0.1 | 0.099 | . | -0.15 | 0.15 |  | -0.16 | 0.156 |  | -0.16 | 0.149 |  |
| Day8 | -0.1 | 0.133 |  | -0.51 | 0 | *** | -0.51 | 0 | *** | -0.51 | 0 | *** |
| Day9 | -0.4 | 0 | *** | -0.87 | 0 | *** | -0.88 | 0 | *** | -0.88 | 0 | *** |
| Day10 | -0.5 | 0 | *** | -1.14 | 0 | *** | -1.14 | 0 | *** | -1.14 | 0 | *** |
| Day11 | -0.8 | 0 | *** | -1.51 | 0 | *** | -1.51 | 0 | *** | -1.51 | 0 | *** |
| Day12 | -0.9 | 0 | *** | -1.74 | 0 | *** | -1.74 | 0 | *** | -1.74 | 0 | *** |
| Day13 | -1.1 | 0 | *** | -2.03 | 0 | *** | -2.03 | 0 | *** | -2.03 | 0 | *** |
| Day14 | -1.3 | 0 | *** | -2.23 | 0 | *** | -2.23 | 0 | *** | -2.23 | 0 | *** |
| AM Swallow | 0.1 | 0.026 | * | 0.05 | 0.322 |  | 0.05 | 0.31 |  | 0.05 | 0.322 |  |
| PM Rest | -0.3 | 0 | *** | -0.29 | 0 | *** | -0.29 | 0 | *** | -0.29 | 0 | *** |
| PM Swallow | 0 | 0.478 |  | -0.03 | 0.571 |  | -0.03 | 0.599 |  | -0.03 | 0.574 |  |
| Marri Honey | -0.1 | 0.616 |  | 0.23 | 0.157 |  | 0.24 | 0.155 |  | 0.24 | 0.148 |  |
| Placebo | 0.2 | 0.271 |  | 0.27 | 0.105 |  | 0.27 | 0.104 |  | 0.27 | 0.102 |  |
| Manuka Honey | 0 | 0.87 |  | 0.08 | 0.648 |  | 0.08 | 0.642 |  | 0.08 | 0.632 |  |
| Age.f5-6.99 | -1 | 0 | *** | -0.69 | 0 | *** | -0.69 | 0 | *** | -0.69 | 0 | *** |
| Age.f7-8.99 | -1.5 | 0 | *** | -1.15 | 0 | *** | -1.15 | 0 | *** | -1.15 | 0 | *** |
| Age.f9 and over | -0.9 | 0.037 | * | -0.85 | 0.011 | * | -0.84 | 0.012 | * | -0.84 | 0.013 | * |
| weight | -0.1 | 0.029 | * |  |  |  |  |  |  |  |  |  |
| height | 0.1 | 0.001 | ** | 0.02 | 0 | *** | 0.02 | 0 | *** | 0.02 | 0 | *** |
| BMI | 0.1 | 0.051 | . |  |  |  |  |  |  |  |  |  |
| ome_weight | 1 | 0.122 |  | 0.5 | 0.17 |  | 0.5 | 0.165 |  | 0.51 | 0.158 |  |
| pain_parent | 0.3 | 0.005 | ** | 0.1 | 0.119 |  | 0.1 | 0.124 |  | 0.1 | 0.124 |  |
| pain_child | -0.1 | 0.144 |  |  |  |  |  |  |  |  |  |  |
| BLpain_pm_swallowBL | 0.2 | 0 | *** | 0.09 | 0 | *** | 0.09 | 0 | *** | 0.09 | 0 | *** |
| BLpppmscore_calcBL | -0.1 | 0.01 | * |  |  |  |  |  |  |  |  |  |
| pppmscore_calc | 0.3 | 0 | *** | 0.17 | 0 | *** | 0.17 | 0 | *** | 0.17 | 0 | *** |
| medsgiven_oxy | 0.5 | 0 | *** | 0.56 | 0 | *** | 0.55 | 0 | *** | 0.55 | 0 | *** |
| medsgiven_ibu | 0.4 | 0 | *** | 0.37 | 0 | *** | 0.37 | 0 | *** | 0.37 | 0 | *** |
| medsgiven_parac | 0.3 | 0 | *** | 0.31 | 0 | *** | 0.3 | 0 | *** | 0.3 | 0 | *** |

**Table S6: Results of pooled Wald tests for interaction terms in the m=100, 150 and 200 models. Terms were tested sequentially moving down the column (i.e., if a term was not retained, it was removed from the model before testing the next term). Here we consider moving terms which appear in >50% but <90% of models.**

| **Term** | **m=100** | | **m=150** | | **m=200** | |
| --- | --- | --- | --- | --- | --- | --- |
|  | **p-value** | **Retain** | **p-value** | **Retain** | **p-value** | **Retain** |
| AMPMSwallowRest:BlindTreatment | 0.517 | NO | 0.535 | NO | 0.549 | NO |
| ome_weight | 0.171 | NO | 0.165 | NO | 0.158 | NO |
| Day:AMPMSwallowRest | 0.167 | NO | 0.148 | NO | 0.147 | NO |
| pain_parent | 0.140 | NO | 0.146 | NO | 0.146 | NO |

**Table S7: Models with terms which appear in >50% but <90% of models for each m omitted based on the results of pooled Wald tests (for the multiply imputed datasets). For the CCA model, terms were removed based on the results of likelihood ration tests. Model estimates for the complete case analysis (CCA) and for the pooled reduced models for the sets of imputed datasets with m=100,150 and 200 are shown. Significance based on p-value is marked as . p<0.100, *p<0.050, **p<0.010, ***p<0.001. Reference levels are omitted for brevity**.

| **Variable** | **CCA** | | | **m=100** | | | **m=150** | | | **m=200** | | |
| --- | --- | --- | --- | --- | --- | --- | --- | --- | --- | --- | --- | --- |
|  | **coef** | **p** | **sig** | **coef** | **p** | **sig** | **coef** | **p** | **sig** | **coef** | **p** | **sig** |
| (Intercept) | -4.98 | 0.017 | * | -0.39 | 0.542 |  | -0.39 | 0.54 |  | -0.38 | 0.551 |  |
| Day2 | 0.25 | 0.052 | . | 0.11 | 0.513 |  | 0.12 | 0.486 |  | 0.13 | 0.475 |  |
| Day3 | 0.62 | 0 | *** | 0.33 | 0.072 | . | 0.33 | 0.07 | . | 0.33 | 0.074 | . |
| Day4 | 0.4 | 0.002 | ** | 0.18 | 0.344 |  | 0.18 | 0.321 |  | 0.18 | 0.336 |  |
| Day5 | 0.47 | 0 | *** | 0.23 | 0.21 |  | 0.24 | 0.194 |  | 0.24 | 0.2 |  |
| Day6 | 0.36 | 0.007 | ** | 0.2 | 0.292 |  | 0.19 | 0.303 |  | 0.19 | 0.298 |  |
| Day7 | 0.07 | 0.605 |  | -0.15 | 0.393 |  | -0.16 | 0.379 |  | -0.16 | 0.385 |  |
| Day8 | 0.1 | 0.477 |  | -0.36 | 0.077 | . | -0.35 | 0.083 | . | -0.35 | 0.08 | . |
| Day9 | -0.16 | 0.245 |  | -0.72 | 0 | *** | -0.72 | 0 | *** | -0.72 | 0 | *** |
| Day10 | -0.42 | 0.002 | ** | -1.07 | 0 | *** | -1.06 | 0 | *** | -1.05 | 0 | *** |
| Day11 | -0.62 | 0 | *** | -1.37 | 0 | *** | -1.37 | 0 | *** | -1.36 | 0 | *** |
| Day12 | -0.76 | 0 | *** | -1.54 | 0 | *** | -1.53 | 0 | *** | -1.53 | 0 | *** |
| Day13 | -0.86 | 0 | *** | -1.66 | 0 | *** | -1.66 | 0 | *** | -1.66 | 0 | *** |
| Day14 | -1.11 | 0 | *** | -1.98 | 0 | *** | -1.98 | 0 | *** | -1.98 | 0 | *** |
| AM Swallow | 0.16 | 0.023 | * | 0.05 | 0.321 |  | 0.05 | 0.31 |  | 0.05 | 0.322 |  |
| PM Rest | -0.34 | 0 | *** | -0.29 | 0 | *** | -0.29 | 0 | *** | -0.29 | 0 | *** |
| PM Swallow | 0.06 | 0.394 |  | -0.03 | 0.571 |  | -0.03 | 0.599 |  | -0.03 | 0.573 |  |
| Marri Honey | 0.21 | 0.417 |  | 0.5 | 0.038 | * | 0.5 | 0.036 | * | 0.52 | 0.033 | * |
| Placebo | 0.41 | 0.11 |  | 0.33 | 0.182 |  | 0.34 | 0.172 |  | 0.34 | 0.165 |  |
| Manuka Honey | 0.02 | 0.945 |  | 0.1 | 0.686 |  | 0.1 | 0.67 |  | 0.11 | 0.648 |  |
| Age.f5-6.99 | -0.92 | 0 | *** | -0.7 | 0 | *** | -0.7 | 0 | *** | -0.7 | 0 | *** |
| Age.f7-8.99 | -1.44 | 0 | *** | -1.19 | 0 | *** | -1.19 | 0 | *** | -1.19 | 0 | *** |
| Age.f9 and over | -0.9 | 0.036 | * | -0.87 | 0.01 | * | -0.86 | 0.011 | * | -0.86 | 0.011 | * |
| weight | -0.06 | 0.038 | * |  |  |  |  |  |  |  |  |  |
| height | 0.05 | 0.002 | ** | 0.02 | 0 | *** | 0.02 | 0 | *** | 0.02 | 0 | *** |
| BMI | 0.1 | 0.073 | . |  |  |  |  |  |  |  |  |  |
| pain_parent | 0.23 | 0.011 | * |  |  |  |  |  |  |  |  |  |
| BLpain_pm_swallowBL | 0.17 | 0 | *** | 0.09 | 0 | *** | 0.09 | 0 | *** | 0.09 | 0 | *** |
| BLpppmscore_calcBL | -0.06 | 0.013 | * |  |  |  |  |  |  |  |  |  |
| pppmscore_calc | 0.27 | 0 | *** | 0.17 | 0 | *** | 0.17 | 0 | *** | 0.17 | 0 | *** |
| medsgiven_oxy | 0.45 | 0 | *** | 0.56 | 0 | *** | 0.55 | 0 | *** | 0.55 | 0 | *** |
| medsgiven_ibu | 0.46 | 0 | *** | 0.38 | 0 | *** | 0.38 | 0 | *** | 0.38 | 0 | *** |
| medsgiven_parac | 0.33 | 0 | *** | 0.31 | 0 | *** | 0.3 | 0 | *** | 0.3 | 0 | *** |
| Day2:Marri Honey | -0.05 | 0.786 |  | -0.05 | 0.865 |  | -0.06 | 0.835 |  | -0.07 | 0.8 |  |
| Day3:Marri Honey | -0.48 | 0.013 | * | -0.26 | 0.327 |  | -0.25 | 0.347 |  | -0.26 | 0.338 |  |
| Day4:Marri Honey | 0.05 | 0.785 |  | 0.03 | 0.928 |  | 0.03 | 0.924 |  | 0.02 | 0.929 |  |
| Day5:Marri Honey | -0.2 | 0.306 |  | -0.27 | 0.334 |  | -0.27 | 0.329 |  | -0.28 | 0.318 |  |
| Day6:Marri Honey | -0.16 | 0.407 |  | -0.19 | 0.494 |  | -0.19 | 0.51 |  | -0.19 | 0.503 |  |
| Day7:Marri Honey | 0.01 | 0.949 |  | -0.27 | 0.315 |  | -0.27 | 0.322 |  | -0.28 | 0.315 |  |
| Day8:Marri Honey | -0.25 | 0.202 |  | -0.31 | 0.296 |  | -0.31 | 0.285 |  | -0.32 | 0.273 |  |
| Day9:Marri Honey | -0.33 | 0.08 | . | -0.33 | 0.232 |  | -0.32 | 0.221 |  | -0.33 | 0.217 |  |
| Day10:Marri Honey | -0.19 | 0.319 |  | -0.13 | 0.646 |  | -0.15 | 0.587 |  | -0.17 | 0.554 |  |
| Day11:Marri Honey | -0.41 | 0.033 | * | -0.3 | 0.243 |  | -0.29 | 0.253 |  | -0.31 | 0.241 |  |
| Day12:Marri Honey | -0.36 | 0.058 | . | -0.39 | 0.12 |  | -0.4 | 0.115 |  | -0.41 | 0.101 |  |
| Day13:Marri Honey | -0.47 | 0.014 | * | -0.53 | 0.025 | * | -0.53 | 0.027 | * | -0.55 | 0.024 | * |
| Day14:Marri Honey | -0.32 | 0.093 | . | -0.47 | 0.047 | * | -0.47 | 0.046 | * | -0.48 | 0.041 | * |
| Day2:Placebo | 0.05 | 0.798 |  | 0.12 | 0.671 |  | 0.11 | 0.707 |  | 0.1 | 0.737 |  |
| Day3:Placebo | 0.02 | 0.908 |  | 0.08 | 0.772 |  | 0.08 | 0.769 |  | 0.09 | 0.753 |  |
| Day4:Placebo | 0.21 | 0.277 |  | 0.27 | 0.366 |  | 0.27 | 0.352 |  | 0.28 | 0.325 |  |
| Day5:Placebo | 0.23 | 0.235 |  | 0.39 | 0.188 |  | 0.38 | 0.203 |  | 0.38 | 0.215 |  |
| Day6:Placebo | 0.15 | 0.417 |  | 0.17 | 0.54 |  | 0.17 | 0.538 |  | 0.17 | 0.559 |  |
| Day7:Placebo | -0.15 | 0.426 |  | 0.07 | 0.826 |  | 0.07 | 0.818 |  | 0.06 | 0.843 |  |
| Day8:Placebo | -0.31 | 0.112 |  | -0.17 | 0.56 |  | -0.18 | 0.54 |  | -0.18 | 0.533 |  |
| Day9:Placebo | -0.44 | 0.023 | * | -0.28 | 0.367 |  | -0.29 | 0.337 |  | -0.29 | 0.319 |  |
| Day10:Placebo | -0.18 | 0.336 |  | -0.11 | 0.69 |  | -0.13 | 0.64 |  | -0.13 | 0.629 |  |
| Day11:Placebo | -0.28 | 0.153 |  | -0.17 | 0.52 |  | -0.19 | 0.489 |  | -0.19 | 0.474 |  |
| Day12:Placebo | -0.28 | 0.154 |  | -0.22 | 0.379 |  | -0.24 | 0.361 |  | -0.24 | 0.34 |  |
| Day13:Placebo | -0.56 | 0.004 | ** | -0.54 | 0.032 | * | -0.54 | 0.032 | * | -0.54 | 0.03 | * |
| Day14:Placebo | -0.42 | 0.03 | * | -0.29 | 0.251 |  | -0.3 | 0.23 |  | -0.31 | 0.217 |  |
| Day2:Manuka Honey | -0.22 | 0.258 |  | 0.03 | 0.919 |  | 0.02 | 0.947 |  | 0.02 | 0.946 |  |
| Day3:Manuka Honey | 0.19 | 0.336 |  | 0.22 | 0.436 |  | 0.23 | 0.396 |  | 0.22 | 0.397 |  |
| Day4:Manuka Honey | 0.03 | 0.88 |  | 0.11 | 0.681 |  | 0.1 | 0.685 |  | 0.11 | 0.673 |  |
| Day5:Manuka Honey | -0.02 | 0.901 |  | 0.06 | 0.799 |  | 0.05 | 0.851 |  | 0.05 | 0.855 |  |
| Day6:Manuka Honey | -0.3 | 0.123 |  | -0.15 | 0.556 |  | -0.15 | 0.563 |  | -0.16 | 0.542 |  |
| Day7:Manuka Honey | 0.38 | 0.059 | . | 0.22 | 0.414 |  | 0.22 | 0.402 |  | 0.21 | 0.437 |  |
| Day8:Manuka Honey | -0.28 | 0.164 |  | -0.12 | 0.682 |  | -0.12 | 0.67 |  | -0.13 | 0.643 |  |
| Day9:Manuka Honey | -0.08 | 0.686 |  | -0.01 | 0.968 |  | -0.01 | 0.973 |  | -0.01 | 0.961 |  |
| Day10:Manuka Honey | 0 | 0.993 |  | -0.02 | 0.925 |  | -0.03 | 0.903 |  | -0.04 | 0.869 |  |
| Day11:Manuka Honey | 0.09 | 0.646 |  | -0.03 | 0.894 |  | -0.04 | 0.872 |  | -0.05 | 0.855 |  |
| Day12:Manuka Honey | -0.01 | 0.96 |  | -0.15 | 0.525 |  | -0.16 | 0.494 |  | -0.17 | 0.482 |  |
| Day13:Manuka Honey | -0.07 | 0.733 |  | -0.36 | 0.111 |  | -0.37 | 0.112 |  | -0.37 | 0.108 |  |
| Day14:Manuka Honey | 0.09 | 0.65 |  | -0.18 | 0.415 |  | -0.19 | 0.406 |  | -0.2 | 0.388 |  |
| AM Swallow:Marri Honey | -0.26 | 0.011 | * |  |  |  |  |  |  |  |  |  |
| PM Rest:Marri Honey | 0.12 | 0.245 |  |  |  |  |  |  |  |  |  |  |
| PM Swallow:Marri Honey | -0.17 | 0.1 |  |  |  |  |  |  |  |  |  |  |
| AM Swallow:Placebo | 0.02 | 0.883 |  |  |  |  |  |  |  |  |  |  |
| PM Rest:Placebo | -0.05 | 0.624 |  |  |  |  |  |  |  |  |  |  |
| PM Swallow:Placebo | -0.14 | 0.174 |  |  |  |  |  |  |  |  |  |  |
| AM Swallow:Manuka Honey | -0.08 | 0.458 |  |  |  |  |  |  |  |  |  |  |
| PM Rest:Manuka Honey | -0.05 | 0.651 |  |  |  |  |  |  |  |  |  |  |
| PM Swallow:Manuka Honey | -0.05 | 0.662 |  |  |  |  |  |  |  |  |  |  |

## Limitations

For multi-item scores such as the PPPM score, it is generally recommended that individual item scores be imputed rather than the total summed score[6]. However, the PPPM used here has 14-items and was measured repeatedly over 14 days; this resulted in a very large number of variables for imputation. Including the individual items in the imputation prediction model resulted in failure, with more predictors per variable than participants in each treatment group. For this reason, total PPPM scores only were imputed and used in the analysis models.

# Appendix S3: Honey characterisation data

Honeys used: Marri (*Corymbia callophylla*) honey and Western Australian Manuka (*Leptospermum scoparium*) honey. The placebo “honey” was made from Glucose syrup, Rice Malt Syrup, and PharmAust™ Syrup BP and contained no additives, artificial flavours, artificial colours, or preservatives. Honeys were characterised using standardised testing.

Physiochemically, the two honeys and the placebo had similar refractive indices, Brix values and water content below the maximum permissible level of 20% as per the Codex Alimentarius for Honey (Appendix S3, Table S1) [7].

**Appendix S3, Table S1: Physicochemical Parameters**

| **Honey** | **Refractive index (n_D20_)** | **BRIX**  **(°Bx)** | **Moisture Content (in %)** | **Fructose (ng/µL)** | **Glucose (ng/µL)** | **Sucrose**  **(ng/µL)** | **Maltose**  **(ng/µL)** |
| --- | --- | --- | --- | --- | --- | --- | --- |
|  |  |  |  | **Mean**  **± SD** | **Mean**  **± SD** |  |  |
| Marri | 1.4950 | 81.8 | 16.6 | 403.17  ± 7.49 | 279.94  ± 6.34 | 0 | 4.70 |
| Manuka | 1.4949 | 81.6 | 16.6 | 387.36 ± 0.59 | 316.75  ± 2.47 | 0 | 4.00 |
| Placebo Honey | 1.4954 | 81.8 | 16.4 | 0 | 203.65  ± 2.06 | 66.30 | 205.3 |

All three honey samples were extracted with dichloromethane and the resulting non-sugar fractions analysed by High Performance Thin-Layer Chromatography (HPTLC) following established protocols.[8,9] The resulting HPTLC fingerprints (Appendix S3, Figure S1) provide a visual representation of the different chemical compositions of the honeys. As expected, the placebo honey, is mostly void of non-sugar constituents and thus presents a rather ‘pale’ fingerprint, whereas Marri and Manuka honey are rich in non-sugar constituents, signified by bands of varying colours at different positions across the HPTLC plate.


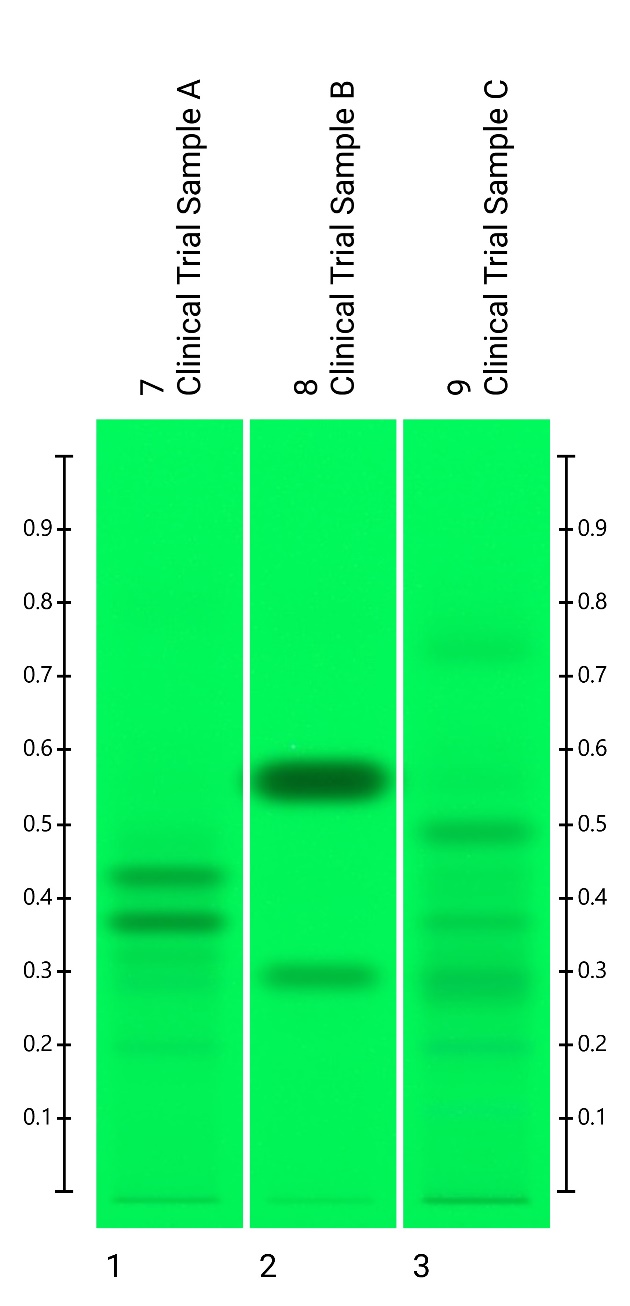

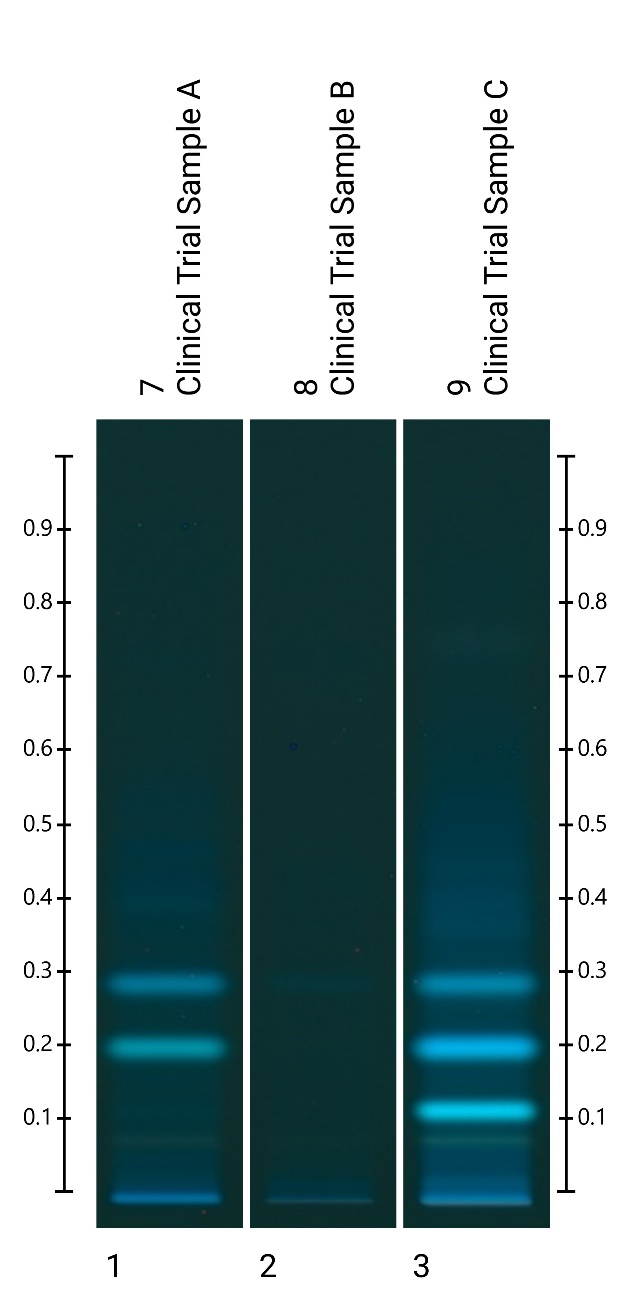


| 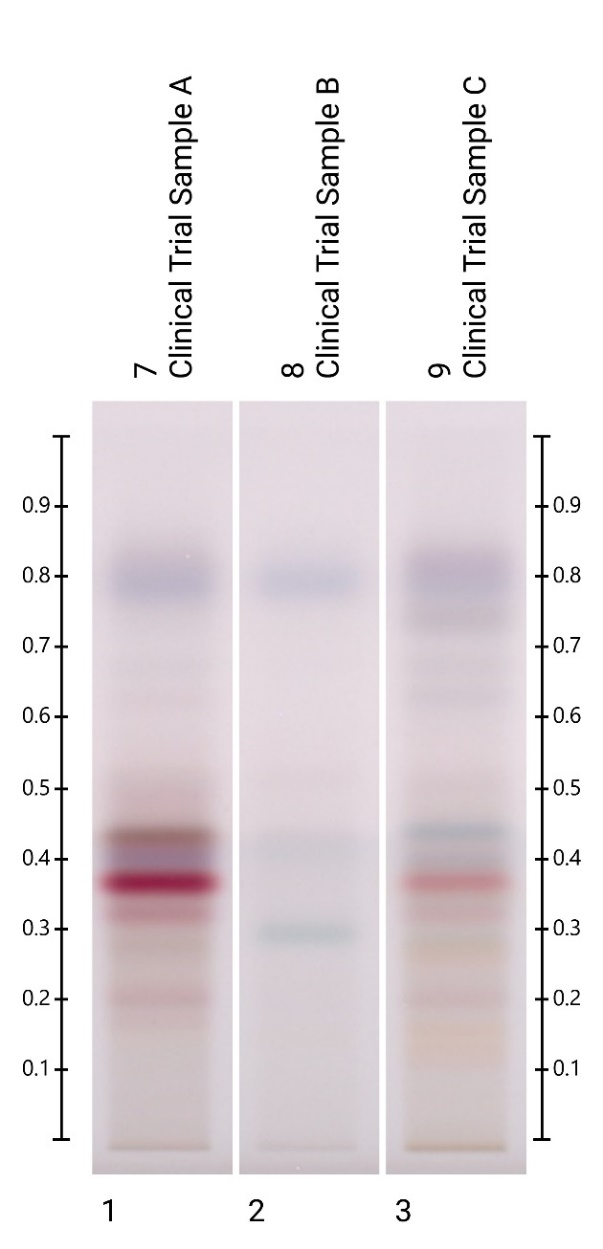 | 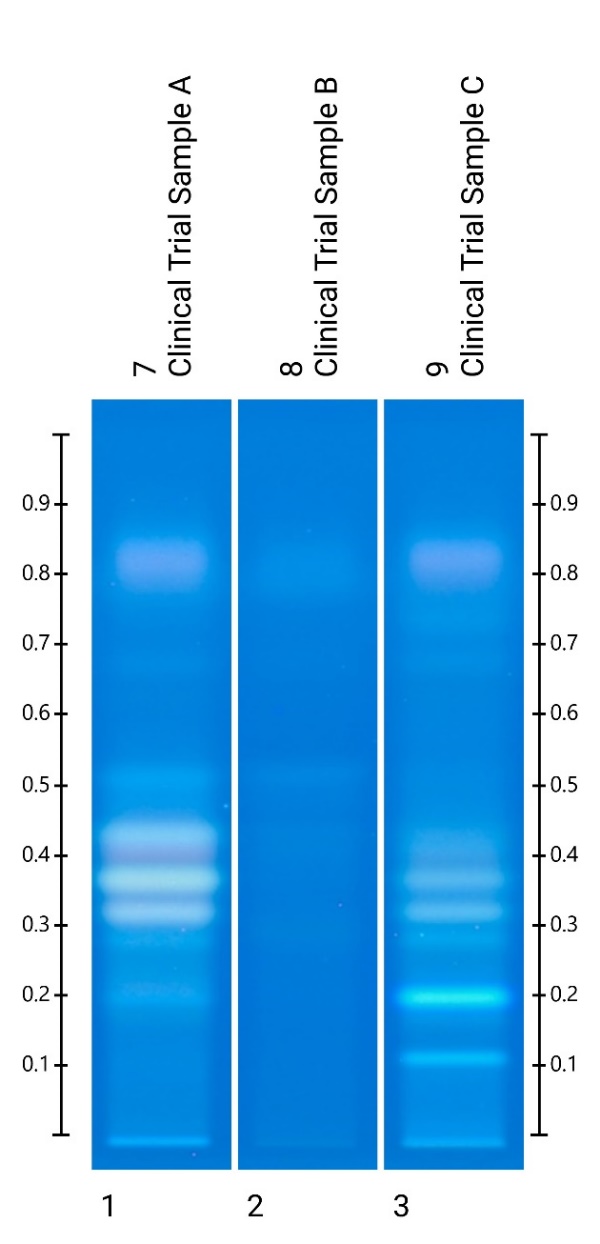 |
| --- | --- |

Appendix S3 Figure S1**:** HPTLC images of honey organic extracts (5 µL). Images taken at (a) 254 nm; (b) 366 nm; (c) White light after derivatisation and (d) 366 nm after derivatisation with vanillin reagent; Track 1— Marri; Track 2— Placebo honey; Track 3—Manuka

Antibacterial activity [10] and methyl glyoxal (MGO levels [11] were determined. Further, the total phenolic content of each honey was measured, and antioxidant activity was determined via the Ferric Reducing Antioxidant Power (FRAP) and 2,2-diphenyl-1-picrylhydrazyl (DPPH) assays.

Antibacterial activity and antioxidant activities (Appendix S3, Tables S2 and S3) were superior in the honeys. Placebo “honey” has some antibacterial activity due to its high osmolarity. As expected, only Manuka honey had substantial MGO levels (Appendix S3, Table S2).

| **Honey** | **Minimum Inhibitory Concentrations (MICs)** | | | | | | **Methyl glyoxal level**  **(ppm)** |
| --- | --- | --- | --- | --- | --- | --- | --- |
|  | ***S. aureus*** | ***E. faecalis*** | ***E. coli*** | ***P. aeruginosa*** | **AAV** | **PE** |  |
| Marri | >30 | >30 | >30 | 24 | 212 | < 5 | 3 |
| Manuka | 16 | 30 | 17 | 24 | 383 | < 5 | 174 |
| Placebo | >30 | >30 | >30 | >30 | 132 | < 5 | 0 |

Appendix S3, Table S2: Antibacterial Activity of the three honey samples

- AAV= Antibacterial Activity Value, Assay of bacterial growth with 4 bacteria in a broth with tested honey, the lowest activity honeys typically have AAVs of around 150, which is antibacterial activity due to the osmotic activity of the sugar content. **Manuka honey showed the highest levels of bacterial inhibition and had substantial MGO levels**.
- PE= Phenol Equivalence Assay: Method used to compare zones of antimicrobial growth inhibition versus phenol standards but underestimates antibacterial activity of some honeys.

Appendix S3, Table S3: Antioxidant activity of the three honey samples

| **Honey** | **Total Phenolic Content** | | | **Ferric Reducing Antioxidant Power Activity (FRAP)** | | | **2,2-Diphenyl-1-picrylhydrazyl (DPPH) Assay** | | |
| --- | --- | --- | --- | --- | --- | --- | --- | --- | --- |
|  | mg GAE* / 100 g | SD | RSD (%) | mmol Fe^2+^ / kg | SD | RSD (%) | µmol TE^#^ / kg | SD | RSD (%) |
| Marri | 29.882 | 0.564 | 1.888 | 4.263 | 0.393 | 9.227 | 2076.05 | 168.64 | 8.12 |
| Manuka | 37.638 | 0.722 | 1.919 | 5.321 | 0.186 | 3.502 | 2033.75 | 74.10 | 3.64 |
| Placebo | 9.356 | 0.262 | 2.796 | 2.070 | 0.032 | 1.548 | Below levels of detection | | |

*GAE = Gallic acid equivalents; ^#^TE = Trolox Equivalents

- The Total Phenolic Content assay captures phenolic constituents present in a sample, including simple phenolic acids and flavonoids
- Phenolic constituents are commonly associated with antioxidant activity, which can be assessed by the FRAP and the DPPH assays. These assays use different chemical mechanisms to capture antioxidant activity
- A positive correlation between total phenolic content and antioxidant activity has been established in the literature. **The two natural honeys have a much higher total phenolic content and thus higher levels of antioxidant activity than placebo**.

# Appendix S4: Additional data graphs and tables

## Honey Compliance


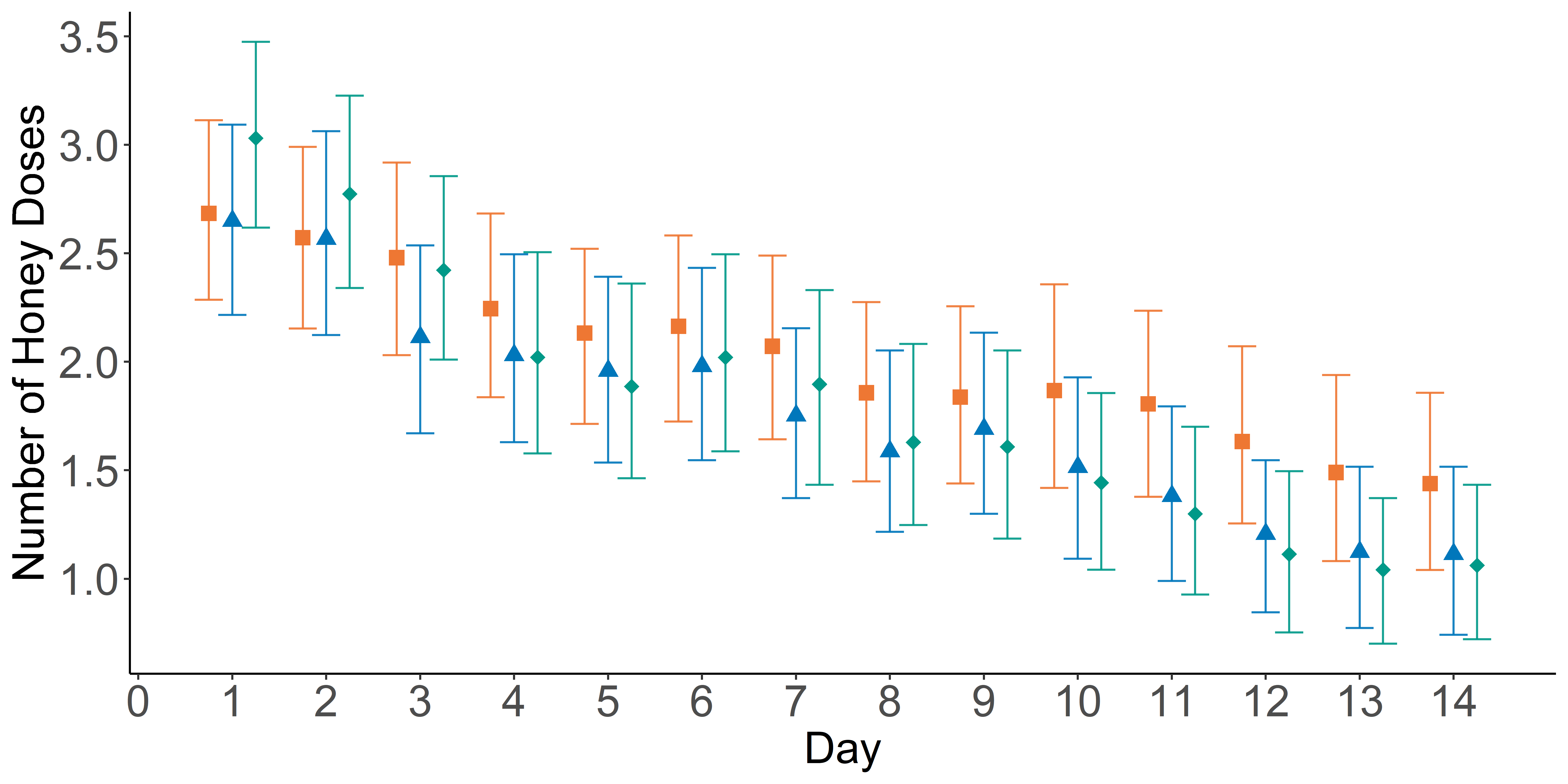


**Figure S1:** Mean number of doses of honey taken in each treatment group (Manuka, orange squares (■); Marri, blue triangles (▲); Placebo, green diamonds (◆)) over the study days, shown with bootstrapped 95% Confidence Intervals of the mean.


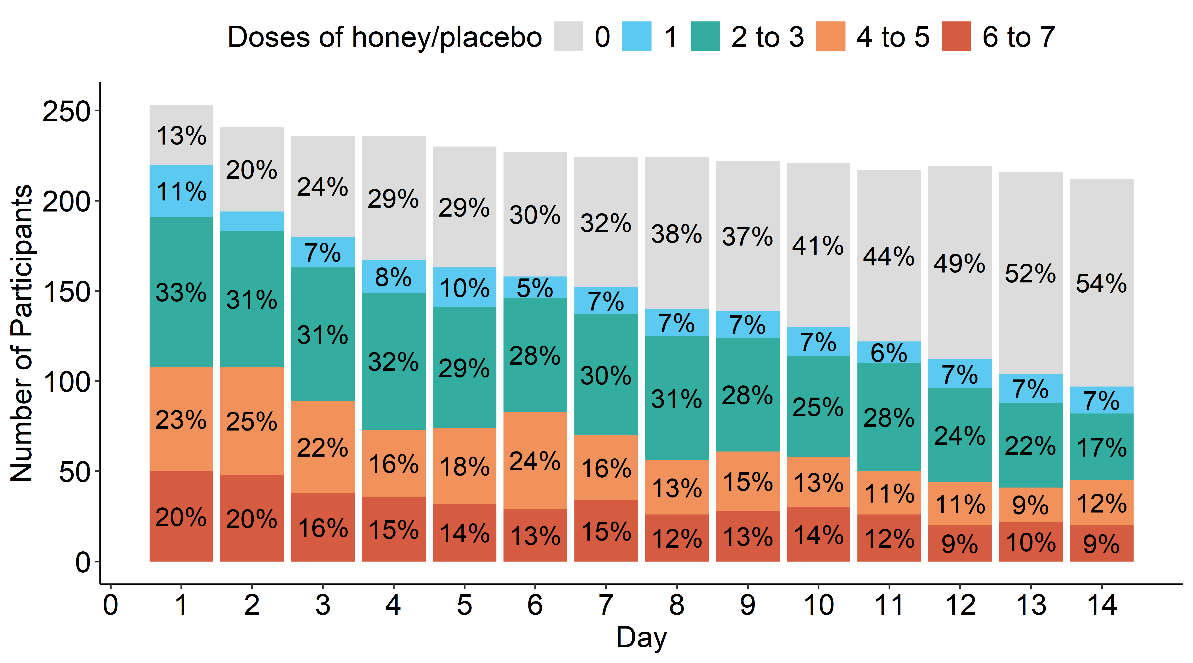


**Figure S2:** Proportion of participants in the honey/placebo groups who reported taking different numbers of honey/placebo doses on each post-operative day, where Day 0 is the day of surgery. Percentages <5% are not annotated in the figure. From top of the bar to bottoms: zero doses=light grey; One dose=light blue; 2-3 doses=green; 4-5 doses=yellow; 6-7 doses=pink. The changing total number of participants reflects the gradually declining number of responses each day.


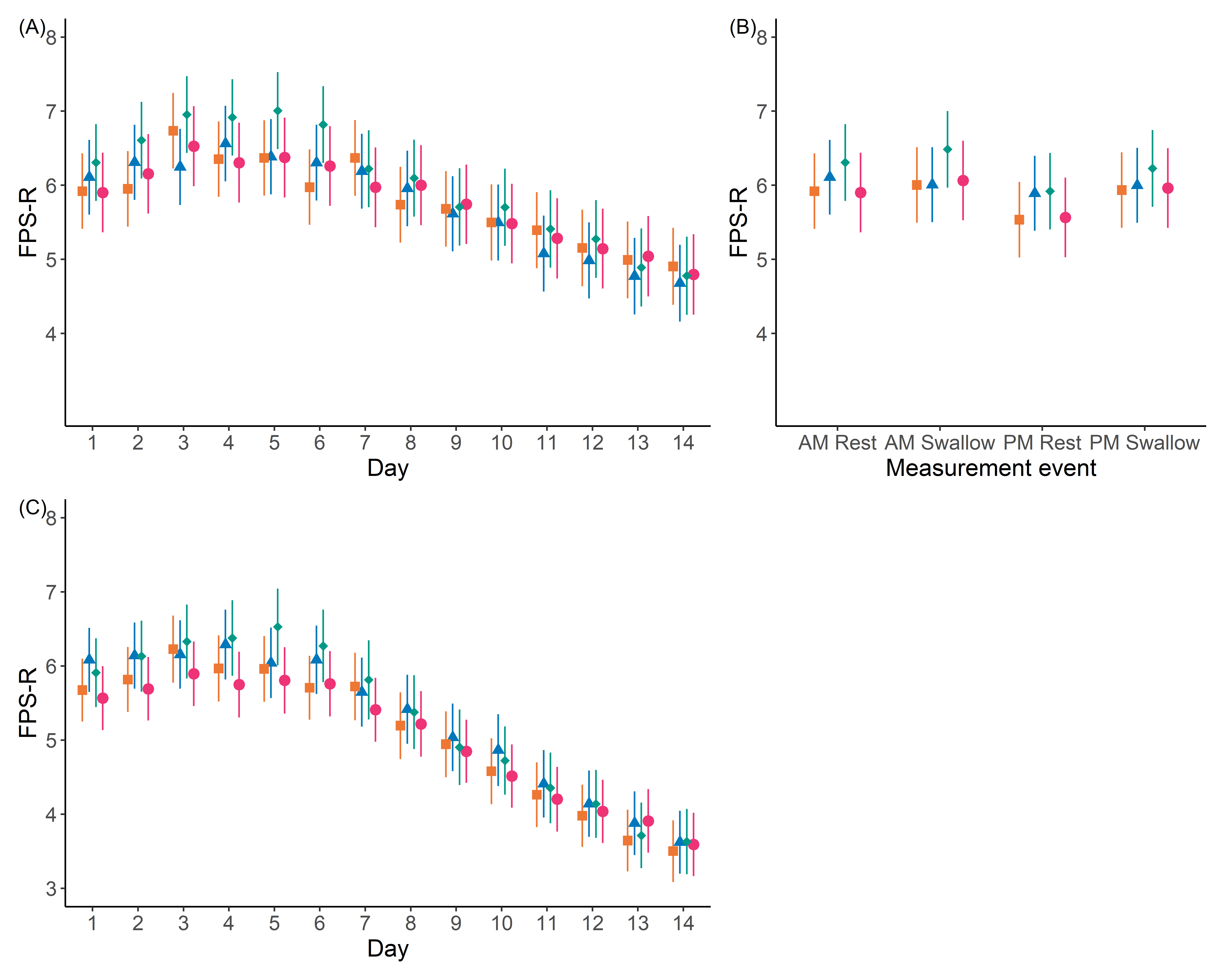


**Figure S3:** The modelling predicted values for FPS-R score (marginal effects) and 95% confidence intervals for the interactions between (A) treatment group with day and (B) treatment group with measurement condition for the final reduced CCA model; (C) treatment group with day for the final reduced imputed data model. (Manuka, orange squares (■); Marri, blue triangles (▲); Placebo, green diamonds (◆); Standard Care, pink filled circles (●)).


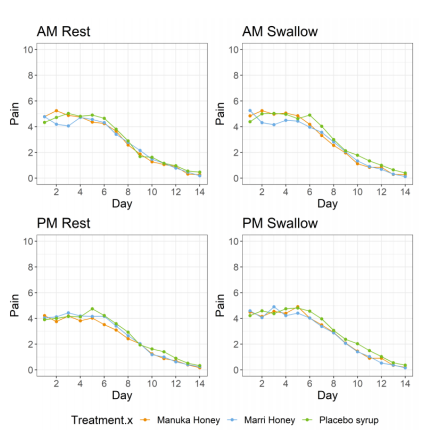


**Figure S4:** As Treated Analysis, mean daily FPS-R scores for compliant participants in the honey (Manuka=orange; Marri=blue) or placebo (green) groups.


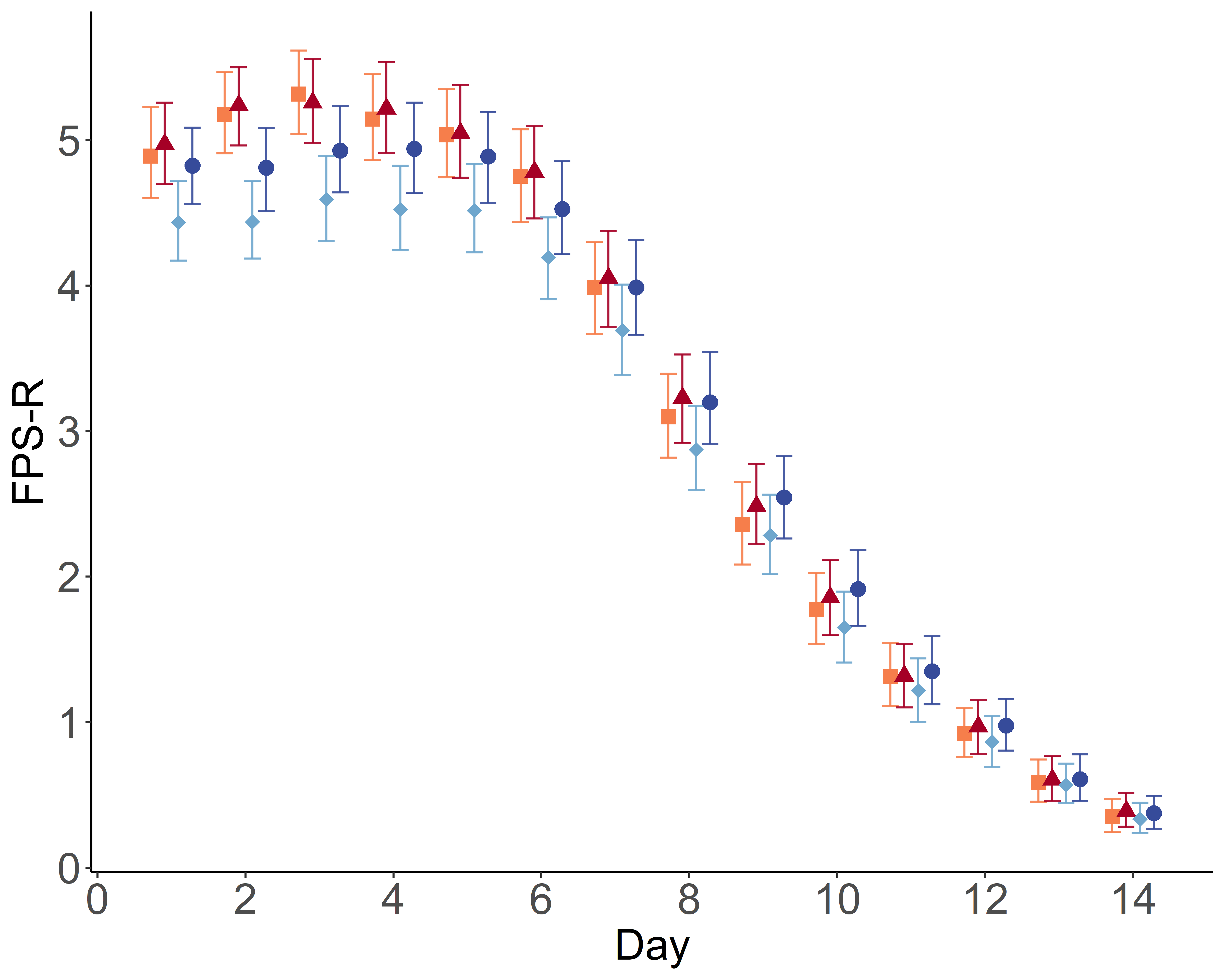


**Figure S5:** Mean pain (from FPS-R) for each event of measurement with bootstrapped 95% Confidence Intervals, for all treatment groups combined at different measurement events throughout each day (AM Rest, orange squares (■); AM Swallow, red triangles (▲); PM Rest, light blue diamonds (◆); PM Swallow, dark blue filled circles (●))


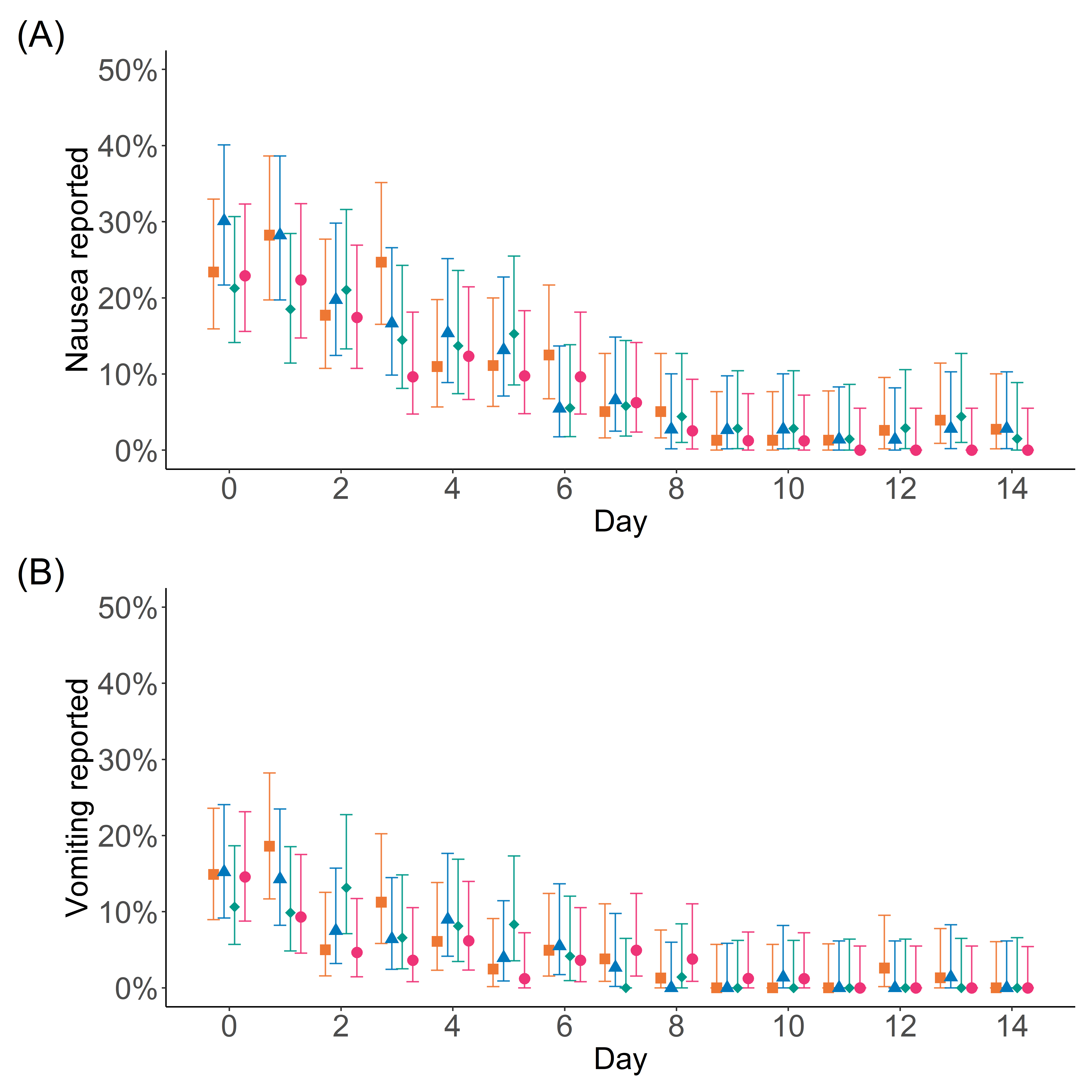


**Figure S6**: Parental reported post-operative (A) nausea & (B) vomiting for each treatment group, shown with Agresti-Coull Confidence Intervals on the mean. (Manuka, orange squares (■); Marri, blue triangles (▲); Placebo, green diamonds (◆); Standard Care, pink filled circles (●)).


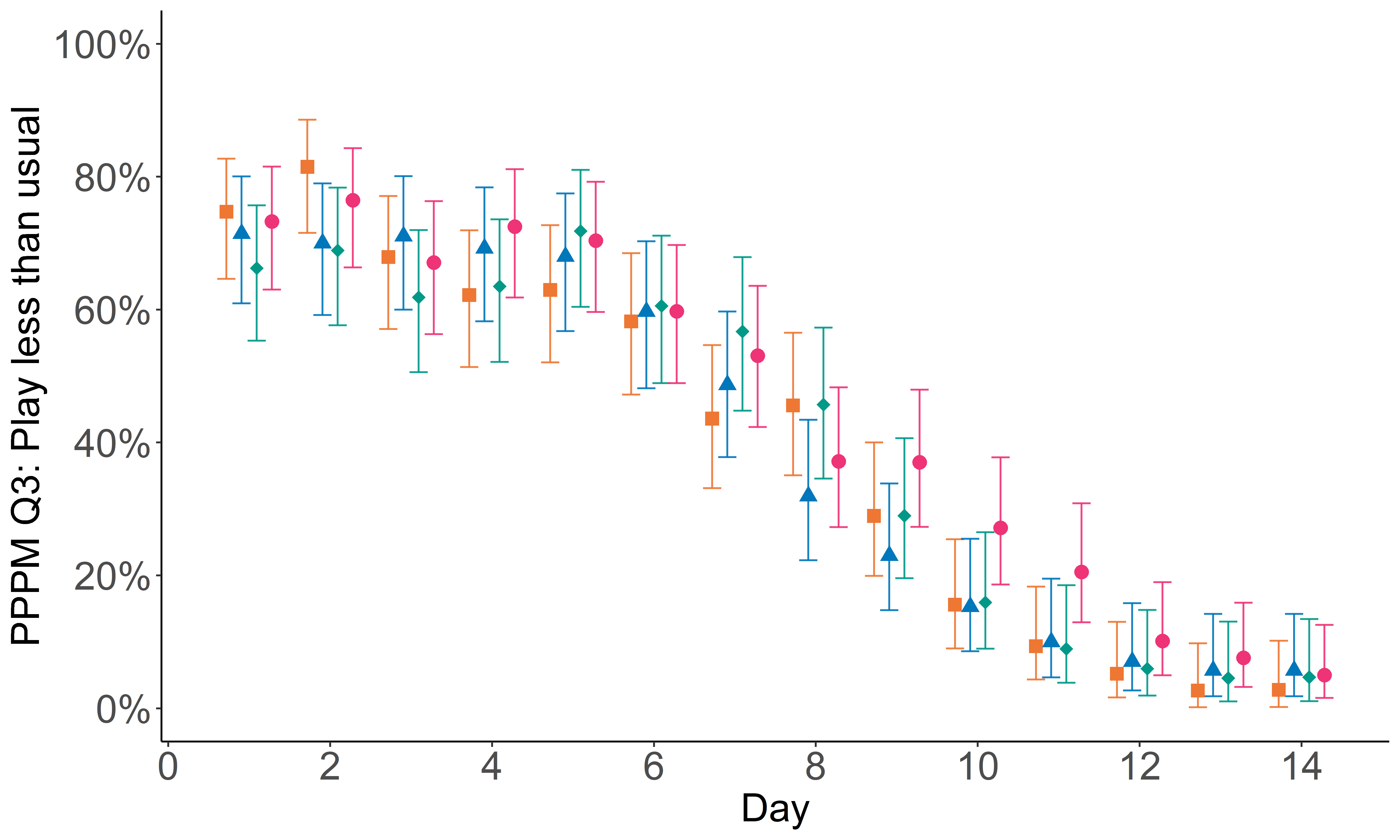


**Figure S7:** Plays less than usual Question from PPPM for each treatment group, shown with bootstrapped 95% Confidence Intervals on the mean. (Manuka, orange squares (■); Marri, blue triangles (▲); Placebo, green diamonds (◆); Standard Care, pink filled circles (●)).

**Table S1** Participant characteristics and intraoperative details and key outcomes over the 14 day follow up period for each of the treatment groups.

|  | Overall (N=391) | Manuka Honey (N=98) | Marri Honey (N=97) | Placebo (N=97) | Standard Care (N=99) |
| --- | --- | --- | --- | --- | --- |
| Participant characteristics |  |  |  |  |  |
| Age, mean ± s.d. | 6.5 ± 3.2 | 6.3 ± 3.5 | 6.6 ± 3.3 | 6.7 ± 3.0 | 6.3 ± 3.5 |
| 4 and under, n (%) | 156 (40%) | 44 (45%) | 36 (37%) | 30 (31%) | 46 (46%) |
| 5-6.99, n (%) | 85 (22%) | 22 (22%) | 23 (24%) | 26 (27%) | 14 (14%) |
| 7-8.99, n (%) | 77 (20%) | 13 (13%) | 20 (21%) | 24 (25%) | 20 (20%) |
| 9 and over, n (%) | 73 (19%) | 19 (19%) | 18 (19%) | 17 (18%) | 19 (19%) |
| Sex (Female), n (%) | 170 (43%) | 42 (43%) | 43 (44%) | 43 (44%) | 42 (42%) |
| BMI z-score, mean ± s.d. | 0.77 ± 2.61 | 0.77 ± 1.37 | 0.88 ± 3.44 | 0.60 ± 1.35 | 0.81 ± 3.38 |
| STBUR score, median (IQR) | 4 (2, 5) | 4 (2, 5) | 4 (2, 5) | 3 (2, 5) | 5 (3, 5) |
| Site |  |  |  |  |  |
| PCH | 280 (72%) | 69 (70%) | 69 (71%) | 71 (73%) | 71 (72%) |
| FSH | 12 (3%) | 4 (4%) | 3 (3%) | 2 (2%) | 3 (3%) |
| SJOG | 99 (25%) | 25 (26%) | 25 (26%) | 24 (25%) | 25 (25%) |
| Anaesthesia details |  |  |  |  |  |
| ASA |  |  |  |  |  |
| I/II/III | 164 (43%) / 211 (56%) / 3(1%) | 46 (51%) / 45 (49%) / 0 | 38 (41%) / 53 (57%) / 2 (2%) | 44 (46%) / 51 (53%) / 1 (1%) | 36 (37%) / 62 (63%) / 0 |
| Missing | 13 | 7 | 4 | 1 | 1 |
| OME per kg intraoperative | 0.41±0.17 | 0.40±0.12 | 0.41±0.14 | 0.42±0.26 | 0.41±0.12 |
|  |  |  |  |  |  |
| **PPPM on Day 7** |  |  |  |  |  |
| Total >=6 | 147 (38%) | 33 (34%) | 32 (33%) | 36 (37%) | 46 (46%) |
| Total >=9 | 97 (25%) | 24 (24%) | 20 (21%) | 26 (27%) | 27 (27%) |
| Whines and complains more than usual | 156 (52%) | 35 (45%) | 36 (47%) | 38 (57%) | 47 (58%) |
| Cries more easily than usual | 130 (43%) | 31 (40%) | 29 (38%) | 32 (48%) | 38 (47%) |
| Plays less than usual | 152 (50%) | 34 (44%) | 37 (49%) | 38 (57%) | 43 (53%) |
| Refuses to eat | 124 (41%) | 28 (36%) | 27 (36%) | 31 (46%) | 38 (48%) |
| Eats less than usual | 206 (68%) | 47 (61%) | 47 (62%) | 46 (68%) | 66 (81%) |
| Missing | 89 | 20 | 21 | 30 | 18 |
| Nausea day of surgery | 92 (24%) | 22 (23%) | 28 (30%) | 20 (21%) | 22 (23%) |
| *Missing | 14 | 4 | 4 | 3 | 3 |
| Vomiting day of surgery | 52 (14%) | 14 (15%) | 14 (15%) | 10 (11%) | 14 (15%) |
| *Missing | 15 | 4 | 5 | 3 | 3 |
| Nausea days 1-3 | 123 (38%) | 34 (43%) | 36 (44%) | 26 (33%) | 27 (33%) |
| *Missing | 67 | 18 | 15 | 18 | 16 |
| Vomiting days 1-3 | 76 (24%) | 21 (26%) | 22 (27%) | 19 (25%) | 14 (17%) |
| *Missing | 71 | 18 | 16 | 20 | 17 |
| Any vomiting days 1-14 | 101 (35%) | 25 (35%) | 30 (39%) | 26 (39%) | 20 (26%) |
| *Missing | 101 | 27 | 21 | 30 | 23 |
| Simple Analgesia dosing compliance+ |  |  |  |  |  |
| Days 1-3 | 188 (48%) | 52 (53%) | 46 (47%) | 43 (44%) | 47 (47%) |
| Days 4-6 | 166 (42%) | 41 (42%) | 42 (43%) | 37 (38%) | 46 (46%) |
| Days 7-9 | 60 (15%) | 14 (14%) | 19 (20%) | 12 (12%) | 15 (15%) |
| Honey dosing compliance^‡^ |  |  |  |  |  |
| Days 1-3 | .. | 47 (48%) | 42 (43%) | 51 (53%) | Not applicable |
| Days 4-6 | .. | 42 (43%) | 42 (43%) | 39 (40%) | Not applicable |
| Days 7-9 | .. | 39 (40%) | 33 (34%) | 35 (36%) | Not applicable |
| Medical re-presentation  days 1-14 | 105 (27%) | 27 (28%) | 27 (28%) | 25 (26%) | 26 (26%) |
| To Primary Care | 46 (12%) | 12 (12%) | 11 (11%) | 10 (10%) | 13 (13%) |
| To hospital | 71 (18%) | 20 (20%) | 18 (19%) | 16 (16%) | 17 (17%) |
| For pain | 43 (11%) | 14 (14%) | 12 (12%) | 7 (7%) | 10 (10%) |
| For bleeding | 40 (10%) | 10 (10%) | 8 (8%) | 9 (9%) | 13 (13%) |
| For vomiting | 13 (3%) | 5 (5%) | 5 (5%) | 3 (3%) | 0 (0%) |
| For infection | 16 (4%) | 5 (5%) | 4 (4%) | 5 (5%) | 2 (2%) |
| For pain ONLY | 29 (7%) | 8 (8%) | 10 (10%) | 4 (4%) | 7 (7%) |
| Other reasons | 45 (12%) | 11 (11%) | 11 (11%) | 9 (9%) | 14 (14%) |
| Presentation to GP >1 day | 7 (2%) | 2 (2%) | 1 (1%) | 1 (1%) | 3 (3%) |
| Presentation to hospital >1 day | 29 (7%) | 10 (10%) | 7 (7%) | 7 (7%) | 5 (5%) |
| Readmission to hospital  days 1-14 | 28 (7%) | 8 (8%) | 6 (6%) | 5 (5%) | 9 (9%) |
| For pain | 2 (0.5%) | 1 (1%) | 1 (1%) | 0 (0%) | 0 (0%) |
| For bleeding | 21 (5%) | 5 (5%) | 4 (4%) | 4 (4%) | 8 (8%) |
| For vomiting | 2 (1%) | 0 (0%) | 2 (2%) | 0 (0%) | 0 (0%) |
| Other reasons | 4 (1%) | 2 (2%) | 0 | 1 (1%) | 1 (1%) |

**Table S2: Number of days where oxycodone was reportedly taken on postoperative days. Records with at least one day where all medication information were completely missing have been removed altogether.**

|  | Marri (N=70) | Manuka (N=70) | Placebo (N=66) | Standard care (N=76) |
| --- | --- | --- | --- | --- |
| Nil Oxycodone taken, n (%) | 4 (6%) | 7 (10%) | 9 (14%) | 6 (8%) |
| Median (LQ, UQ) Number of Oxycodone doses first 7 days | 6 (2, 10) | 5.50 (2, 11) | 7 (2.25, 11) | 7 (3, 11) |
| missing | 1 | 2 | 0 | 1 |
| Median (LQ, UQ) number of doses of oxycodone over 14 days. | 6 (2, 12) | 7 (2, 12) | 8 (2, 11) | 7 (3, 11) |
| missing | 1 | 2 | 1 | 1 |
| Median (LQ, UQ) Number of days where oxycodone was taken | 4 (1, 6) | 4 (2, 7) | 5 (2, 7) | 5 (2.75, 7) |

**Table S3: Mean and bootstrapped 95% CI FPS-R pain for each treatment group at each measurement event over the 14-day follow up period; data correspond to Figure 2 in the main article text.**

|  |  | Pain at AM Rest | | | Pain at AM Swallow | | | Pain at PM Rest | | | Pain at PM Swallow | | |
| --- | --- | --- | --- | --- | --- | --- | --- | --- | --- | --- | --- | --- | --- |
| Day | Treatment | Mean | CI 2.5% | CI 97.5% | Mean | CI 2.5% | CI 97.5% | Mean | CI 2.5% | CI 97.5% | Mean | CI 2.5% | CI 97.5% |
| 1 | Manuka | 4.85 | 4.2 | 5.48 | 4.98 | 4.33 | 5.58 | 4.58 | 4.05 | 5.11 | 4.88 | 4.32 | 5.49 |
| 2 | Manuka | 5.23 | 4.63 | 5.83 | 5.28 | 4.67 | 5.83 | 4.15 | 3.62 | 4.68 | 4.54 | 4 | 5.1 |
| 3 | Manuka | 5.62 | 5.02 | 6.23 | 5.48 | 4.88 | 6.02 | 4.65 | 4.04 | 5.31 | 4.99 | 4.39 | 5.56 |
| 4 | Manuka | 5.15 | 4.54 | 5.71 | 5.33 | 4.77 | 5.9 | 4.37 | 3.79 | 4.98 | 4.9 | 4.37 | 5.51 |
| 5 | Manuka | 4.88 | 4.21 | 5.46 | 5.09 | 4.5 | 5.71 | 4.21 | 3.61 | 4.82 | 4.85 | 4.26 | 5.42 |
| 6 | Manuka | 4.37 | 3.78 | 4.98 | 4.35 | 3.75 | 4.95 | 3.88 | 3.39 | 4.38 | 4.32 | 3.78 | 4.86 |
| 7 | Manuka | 4.06 | 3.35 | 4.75 | 4.06 | 3.42 | 4.81 | 3.65 | 3 | 4.3 | 4.08 | 3.4 | 4.77 |
| 8 | Manuka | 3.15 | 2.52 | 3.84 | 3.22 | 2.6 | 3.85 | 2.73 | 2.15 | 3.32 | 3.16 | 2.61 | 3.73 |
| 9 | Manuka | 2.55 | 1.95 | 3.16 | 2.53 | 1.95 | 3.16 | 2.42 | 1.83 | 3.04 | 2.58 | 2.05 | 3.16 |
| 10 | Manuka | 1.74 | 1.23 | 2.27 | 1.63 | 1.14 | 2.13 | 1.55 | 1.12 | 2.06 | 1.78 | 1.31 | 2.29 |
| 11 | Manuka | 1.39 | 0.97 | 1.83 | 1.33 | 0.93 | 1.78 | 1.21 | 0.8 | 1.65 | 1.31 | 0.89 | 1.77 |
| 12 | Manuka | 0.97 | 0.68 | 1.32 | 0.99 | 0.68 | 1.36 | 0.83 | 0.53 | 1.18 | 1.03 | 0.65 | 1.47 |
| 13 | Manuka | 0.55 | 0.28 | 0.87 | 0.62 | 0.32 | 0.99 | 0.55 | 0.3 | 0.83 | 0.6 | 0.32 | 0.93 |
| 14 | Manuka | 0.34 | 0.16 | 0.55 | 0.4 | 0.18 | 0.63 | 0.3 | 0.14 | 0.52 | 0.36 | 0.15 | 0.59 |
| 1 | Marri | 5.12 | 4.58 | 5.62 | 5.29 | 4.75 | 5.88 | 4.93 | 4.45 | 5.45 | 5.19 | 4.73 | 5.67 |
| 2 | Marri | 5.17 | 4.6 | 5.74 | 5.35 | 4.83 | 5.86 | 5 | 4.52 | 5.52 | 5.24 | 4.69 | 5.86 |
| 3 | Marri | 5.12 | 4.53 | 5.67 | 5 | 4.41 | 5.59 | 4.83 | 4.24 | 5.44 | 5.17 | 4.56 | 5.73 |
| 4 | Marri | 5.37 | 4.77 | 5.94 | 5.22 | 4.66 | 5.75 | 4.96 | 4.35 | 5.58 | 5.15 | 4.51 | 5.77 |
| 5 | Marri | 5.03 | 4.38 | 5.67 | 4.91 | 4.25 | 5.59 | 4.68 | 4 | 5.36 | 4.91 | 4.2 | 5.55 |
| 6 | Marri | 5.01 | 4.36 | 5.64 | 4.71 | 3.99 | 5.35 | 4.48 | 3.85 | 5.12 | 4.51 | 3.88 | 5.14 |
| 7 | Marri | 4.04 | 3.47 | 4.65 | 3.92 | 3.31 | 4.51 | 3.84 | 3.21 | 4.42 | 3.82 | 3.17 | 4.47 |
| 8 | Marri | 3.19 | 2.58 | 3.77 | 3.18 | 2.56 | 3.82 | 3.15 | 2.53 | 3.76 | 3.3 | 2.66 | 3.96 |
| 9 | Marri | 2.41 | 1.88 | 2.95 | 2.47 | 1.91 | 3.05 | 2.43 | 1.85 | 3.04 | 2.62 | 2.04 | 3.18 |
| 10 | Marri | 1.88 | 1.39 | 2.43 | 2 | 1.43 | 2.68 | 1.82 | 1.24 | 2.45 | 2.04 | 1.47 | 2.65 |
| 11 | Marri | 1.34 | 0.89 | 1.83 | 1.25 | 0.76 | 1.72 | 1.21 | 0.79 | 1.7 | 1.3 | 0.87 | 1.77 |
| 12 | Marri | 1.03 | 0.61 | 1.44 | 0.99 | 0.6 | 1.38 | 1 | 0.62 | 1.39 | 0.96 | 0.58 | 1.38 |
| 13 | Marri | 0.72 | 0.37 | 1.11 | 0.65 | 0.31 | 1 | 0.65 | 0.32 | 1 | 0.65 | 0.32 | 1.04 |
| 14 | Marri | 0.44 | 0.2 | 0.76 | 0.46 | 0.2 | 0.8 | 0.44 | 0.2 | 0.75 | 0.44 | 0.18 | 0.77 |
| 1 | Placebo | 4.77 | 4.16 | 5.41 | 4.72 | 4.11 | 5.35 | 4.31 | 3.81 | 4.82 | 4.58 | 4.04 | 5.14 |
| 2 | Placebo | 5.38 | 4.83 | 5.91 | 5.38 | 4.82 | 5.92 | 4.38 | 3.83 | 4.96 | 4.78 | 4.2 | 5.38 |
| 3 | Placebo | 5.49 | 4.92 | 6.08 | 5.49 | 4.91 | 6.05 | 4.57 | 4.01 | 5.2 | 4.85 | 4.2 | 5.49 |
| 4 | Placebo | 5.3 | 4.68 | 5.96 | 5.46 | 4.83 | 6.11 | 4.55 | 3.95 | 5.15 | 5.01 | 4.42 | 5.6 |
| 5 | Placebo | 5.5 | 4.85 | 6.14 | 5.38 | 4.69 | 6 | 4.99 | 4.41 | 5.63 | 5.23 | 4.56 | 5.86 |
| 6 | Placebo | 5.08 | 4.48 | 5.69 | 5.23 | 4.56 | 5.93 | 4.37 | 3.73 | 4.99 | 4.71 | 4.07 | 5.35 |
| 7 | Placebo | 4 | 3.43 | 4.59 | 4.31 | 3.64 | 4.94 | 3.74 | 3.14 | 4.33 | 4.16 | 3.52 | 4.83 |
| 8 | Placebo | 3.09 | 2.55 | 3.68 | 3.3 | 2.75 | 3.83 | 2.93 | 2.36 | 3.49 | 3.22 | 2.6 | 3.79 |
| 9 | Placebo | 2.01 | 1.56 | 2.54 | 2.28 | 1.76 | 2.82 | 2.06 | 1.59 | 2.57 | 2.33 | 1.85 | 2.88 |
| 10 | Placebo | 1.63 | 1.23 | 2.07 | 1.84 | 1.39 | 2.27 | 1.47 | 1.06 | 1.96 | 1.86 | 1.39 | 2.34 |
| 11 | Placebo | 1.21 | 0.84 | 1.6 | 1.32 | 0.96 | 1.72 | 1.26 | 0.87 | 1.74 | 1.34 | 0.91 | 1.82 |
| 12 | Placebo | 0.83 | 0.52 | 1.13 | 0.96 | 0.64 | 1.29 | 0.77 | 0.48 | 1.09 | 0.86 | 0.57 | 1.2 |
| 13 | Placebo | 0.46 | 0.24 | 0.71 | 0.48 | 0.24 | 0.75 | 0.44 | 0.24 | 0.68 | 0.46 | 0.25 | 0.72 |
| 14 | Placebo | 0.33 | 0.14 | 0.58 | 0.32 | 0.14 | 0.53 | 0.26 | 0.11 | 0.42 | 0.32 | 0.14 | 0.55 |
| 1 | Standard Care | 4.81 | 4.22 | 5.38 | 4.88 | 4.27 | 5.49 | 3.92 | 3.39 | 4.47 | 4.65 | 4.09 | 5.22 |
| 2 | Standard Care | 4.94 | 4.37 | 5.54 | 4.96 | 4.46 | 5.49 | 4.23 | 3.7 | 4.73 | 4.68 | 4.2 | 5.18 |
| 3 | Standard Care | 5.05 | 4.46 | 5.64 | 5.07 | 4.48 | 5.66 | 4.33 | 3.82 | 4.86 | 4.71 | 4.19 | 5.28 |
| 4 | Standard Care | 4.78 | 4.12 | 5.42 | 4.88 | 4.28 | 5.54 | 4.22 | 3.65 | 4.78 | 4.7 | 4.06 | 5.25 |
| 5 | Standard Care | 4.79 | 4.18 | 5.39 | 4.84 | 4.26 | 5.46 | 4.24 | 3.66 | 4.8 | 4.61 | 4.02 | 5.2 |
| 6 | Standard Care | 4.6 | 3.96 | 5.19 | 4.88 | 4.19 | 5.55 | 4.1 | 3.5 | 4.71 | 4.57 | 3.93 | 5.18 |
| 7 | Standard Care | 3.85 | 3.26 | 4.41 | 3.94 | 3.26 | 4.65 | 3.54 | 2.99 | 4.14 | 3.91 | 3.33 | 4.53 |
| 8 | Standard Care | 2.96 | 2.42 | 3.53 | 3.22 | 2.66 | 3.76 | 2.71 | 2.19 | 3.25 | 3.11 | 2.54 | 3.73 |
| 9 | Standard Care | 2.41 | 1.91 | 2.93 | 2.62 | 2.1 | 3.1 | 2.21 | 1.73 | 2.72 | 2.61 | 2.1 | 3.18 |
| 10 | Standard Care | 1.84 | 1.4 | 2.34 | 1.95 | 1.48 | 2.41 | 1.76 | 1.32 | 2.24 | 1.98 | 1.5 | 2.51 |
| 11 | Standard Care | 1.3 | 0.92 | 1.69 | 1.36 | 0.96 | 1.8 | 1.19 | 0.82 | 1.61 | 1.45 | 1 | 1.91 |
| 12 | Standard Care | 0.86 | 0.56 | 1.16 | 0.95 | 0.65 | 1.29 | 0.86 | 0.55 | 1.2 | 1.05 | 0.72 | 1.4 |
| 13 | Standard Care | 0.61 | 0.38 | 0.88 | 0.66 | 0.41 | 0.94 | 0.62 | 0.38 | 0.9 | 0.71 | 0.46 | 1 |
| 14 | Standard Care | 0.3 | 0.15 | 0.49 | 0.37 | 0.21 | 0.57 | 0.33 | 0.16 | 0.54 | 0.38 | 0.21 | 0.59 |

**Table S4: Daily PPPM summary data for each treatment group over the 14-day follow-up period; data correspond to Figure 3 in the main article text.**

|  |  | PPPM Total Score | | | PPPM Item 8 Proportion Selected | | | PPPM Item 9 Proportion Selected | | |
| --- | --- | --- | --- | --- | --- | --- | --- | --- | --- | --- |
| Day | Treatment | Mean | CI 2.5% | CI 97.5% | Mean | CI 2.5% | CI 97.5% | Mean | CI 2.5% | CI 97.5% |
| 1 | Manuka | 8.36 | 7.49 | 9.28 | 0.53 | 0.42 | 0.63 | 0.82 | 0.73 | 0.89 |
| 1 | Marri | 8.27 | 7.5 | 9.06 | 0.52 | 0.42 | 0.63 | 0.81 | 0.71 | 0.88 |
| 1 | Placebo | 7 | 6.09 | 7.91 | 0.38 | 0.28 | 0.48 | 0.69 | 0.58 | 0.78 |
| 1 | Standard Care | 8.46 | 7.58 | 9.29 | 0.51 | 0.41 | 0.61 | 0.85 | 0.76 | 0.91 |
| 2 | Manuka | 8.42 | 7.53 | 9.28 | 0.54 | 0.44 | 0.65 | 0.85 | 0.76 | 0.92 |
| 2 | Marri | 8.31 | 7.35 | 9.22 | 0.58 | 0.47 | 0.68 | 0.78 | 0.67 | 0.85 |
| 2 | Placebo | 7.71 | 6.62 | 8.66 | 0.53 | 0.41 | 0.64 | 0.77 | 0.66 | 0.85 |
| 2 | Standard Care | 8.55 | 7.74 | 9.41 | 0.51 | 0.4 | 0.61 | 0.87 | 0.78 | 0.93 |
| 3 | Manuka | 7.91 | 6.79 | 8.86 | 0.59 | 0.48 | 0.69 | 0.8 | 0.7 | 0.88 |
| 3 | Marri | 7.94 | 6.9 | 9 | 0.6 | 0.49 | 0.7 | 0.8 | 0.69 | 0.88 |
| 3 | Placebo | 7.45 | 6.52 | 8.48 | 0.55 | 0.44 | 0.66 | 0.77 | 0.67 | 0.85 |
| 3 | Standard Care | 8.04 | 7.16 | 8.94 | 0.5 | 0.39 | 0.61 | 0.88 | 0.79 | 0.93 |
| 4 | Manuka | 7.73 | 6.71 | 8.73 | 0.57 | 0.47 | 0.67 | 0.74 | 0.64 | 0.83 |
| 4 | Marri | 7.7 | 6.66 | 8.74 | 0.56 | 0.45 | 0.67 | 0.81 | 0.71 | 0.88 |
| 4 | Placebo | 7.29 | 6.21 | 8.33 | 0.54 | 0.43 | 0.65 | 0.73 | 0.62 | 0.82 |
| 4 | Standard Care | 8.23 | 7.28 | 9.19 | 0.54 | 0.43 | 0.64 | 0.89 | 0.8 | 0.94 |
| 5 | Manuka | 7.6 | 6.65 | 8.55 | 0.48 | 0.38 | 0.59 | 0.73 | 0.62 | 0.81 |
| 5 | Marri | 7.49 | 6.42 | 8.52 | 0.51 | 0.4 | 0.62 | 0.78 | 0.68 | 0.86 |
| 5 | Placebo | 8 | 6.88 | 9.07 | 0.49 | 0.38 | 0.61 | 0.8 | 0.69 | 0.88 |
| 5 | Standard Care | 7.91 | 6.92 | 8.9 | 0.58 | 0.47 | 0.68 | 0.85 | 0.76 | 0.91 |
| 6 | Manuka | 6.62 | 5.64 | 7.69 | 0.44 | 0.34 | 0.55 | 0.72 | 0.61 | 0.81 |
| 6 | Marri | 6.88 | 5.81 | 8 | 0.44 | 0.33 | 0.55 | 0.79 | 0.68 | 0.87 |
| 6 | Placebo | 7.54 | 6.47 | 8.71 | 0.54 | 0.42 | 0.65 | 0.73 | 0.61 | 0.82 |
| 6 | Standard Care | 7.75 | 6.89 | 8.76 | 0.61 | 0.5 | 0.71 | 0.89 | 0.8 | 0.94 |
| 7 | Manuka | 5.25 | 4.12 | 6.36 | 0.36 | 0.26 | 0.47 | 0.61 | 0.5 | 0.71 |
| 7 | Marri | 5.45 | 4.32 | 6.53 | 0.36 | 0.26 | 0.47 | 0.62 | 0.51 | 0.72 |
| 7 | Placebo | 6.55 | 5.42 | 7.77 | 0.46 | 0.35 | 0.58 | 0.68 | 0.56 | 0.78 |
| 7 | Standard Care | 6.59 | 5.69 | 7.59 | 0.48 | 0.37 | 0.58 | 0.81 | 0.72 | 0.89 |
| 8 | Manuka | 4.41 | 3.49 | 5.4 | 0.28 | 0.19 | 0.39 | 0.54 | 0.43 | 0.65 |
| 8 | Marri | 4.09 | 2.96 | 5.27 | 0.25 | 0.16 | 0.36 | 0.54 | 0.43 | 0.65 |
| 8 | Placebo | 4.32 | 3.31 | 5.38 | 0.26 | 0.17 | 0.37 | 0.46 | 0.35 | 0.57 |
| 8 | Standard Care | 4.57 | 3.62 | 5.58 | 0.27 | 0.18 | 0.38 | 0.63 | 0.52 | 0.73 |
| 9 | Manuka | 3.04 | 2.11 | 3.99 | 0.15 | 0.08 | 0.25 | 0.39 | 0.28 | 0.5 |
| 9 | Marri | 2.84 | 1.95 | 3.78 | 0.19 | 0.11 | 0.29 | 0.42 | 0.31 | 0.53 |
| 9 | Placebo | 2.98 | 2.14 | 3.88 | 0.16 | 0.09 | 0.27 | 0.42 | 0.31 | 0.54 |
| 9 | Standard Care | 3.79 | 2.95 | 4.69 | 0.22 | 0.14 | 0.32 | 0.57 | 0.46 | 0.67 |
| 10 | Manuka | 2.03 | 1.27 | 2.89 | 0.09 | 0.04 | 0.18 | 0.27 | 0.19 | 0.38 |
| 10 | Marri | 1.91 | 1.14 | 2.86 | 0.1 | 0.05 | 0.19 | 0.25 | 0.16 | 0.36 |
| 10 | Placebo | 1.81 | 1.15 | 2.51 | 0.07 | 0.03 | 0.16 | 0.29 | 0.2 | 0.41 |
| 10 | Standard Care | 3.1 | 2.23 | 4 | 0.15 | 0.09 | 0.24 | 0.44 | 0.34 | 0.55 |
| 11 | Manuka | 1.45 | 0.89 | 2.09 | 0.04 | 0.01 | 0.12 | 0.23 | 0.15 | 0.33 |
| 11 | Marri | 1.43 | 0.81 | 2.24 | 0.07 | 0.03 | 0.16 | 0.23 | 0.14 | 0.34 |
| 11 | Placebo | 1.63 | 1 | 2.32 | 0.06 | 0.02 | 0.15 | 0.21 | 0.13 | 0.33 |
| 11 | Standard Care | 2.12 | 1.39 | 2.96 | 0.1 | 0.05 | 0.19 | 0.35 | 0.25 | 0.46 |
| 12 | Manuka | 1.19 | 0.68 | 1.75 | 0.04 | 0.01 | 0.11 | 0.19 | 0.12 | 0.3 |
| 12 | Marri | 1.26 | 0.62 | 2 | 0.08 | 0.04 | 0.18 | 0.15 | 0.09 | 0.26 |
| 12 | Placebo | 0.95 | 0.55 | 1.47 | 0.01 | 0 | 0.09 | 0.16 | 0.09 | 0.27 |
| 12 | Standard Care | 1.19 | 0.67 | 1.7 | 0.05 | 0.02 | 0.13 | 0.19 | 0.12 | 0.29 |
| 13 | Manuka | 0.81 | 0.33 | 1.43 | 0.04 | 0.01 | 0.12 | 0.12 | 0.06 | 0.21 |
| 13 | Marri | 0.97 | 0.4 | 1.72 | 0.04 | 0.01 | 0.12 | 0.13 | 0.07 | 0.23 |
| 13 | Placebo | 0.95 | 0.53 | 1.45 | 0.03 | 0 | 0.11 | 0.17 | 0.09 | 0.28 |
| 13 | Standard Care | 0.81 | 0.43 | 1.32 | 0.04 | 0.01 | 0.11 | 0.18 | 0.11 | 0.28 |
| 14 | Manuka | 0.39 | 0.1 | 0.77 | 0 | 0 | 0.06 | 0.08 | 0.04 | 0.17 |
| 14 | Marri | 0.81 | 0.31 | 1.47 | 0.03 | 0 | 0.1 | 0.1 | 0.05 | 0.2 |
| 14 | Placebo | 0.61 | 0.29 | 1.02 | 0.03 | 0 | 0.11 | 0.09 | 0.04 | 0.19 |
| 14 | Standard Care | 0.62 | 0.28 | 0.97 | 0.04 | 0.01 | 0.11 | 0.1 | 0.05 | 0.19 |

**Table S5: Mean and bootstrapped 95% CI numbers of analgesia doses per day for each treatment group over the 14-day follow-up period; data correspond to Figure 4 in the main article text.**

|  |  | Ibuprofen + Paracetamol Dose | | | Oxycodone Dose | | |
| --- | --- | --- | --- | --- | --- | --- | --- |
| Day | Treatment | Mean | CI 2.5% | CI 97.5% | Mean | CI 2.5% | CI 97.5% |
| 1 | Manuka | 5.72 | 5.32 | 6.07 | 1.43 | 1.14 | 1.67 |
| 1 | Marri | 5.85 | 5.42 | 6.24 | 1.4 | 1.15 | 1.67 |
| 1 | Placebo | 5.26 | 4.73 | 5.7 | 1.31 | 1.06 | 1.53 |
| 1 | Standard Care | 5.62 | 5.24 | 5.98 | 1.28 | 1.05 | 1.54 |
| 2 | Manuka | 5.68 | 5.27 | 6.11 | 1.25 | 0.95 | 1.51 |
| 2 | Marri | 5.8 | 5.37 | 6.17 | 1.12 | 0.88 | 1.35 |
| 2 | Placebo | 5.49 | 4.99 | 5.91 | 1.28 | 1.05 | 1.53 |
| 2 | Standard Care | 5.86 | 5.48 | 6.19 | 1.33 | 1.08 | 1.6 |
| 3 | Manuka | 5.36 | 4.89 | 5.85 | 1.04 | 0.8 | 1.29 |
| 3 | Marri | 5.59 | 5.13 | 5.99 | 0.99 | 0.73 | 1.24 |
| 3 | Placebo | 5.46 | 4.99 | 5.88 | 1.11 | 0.88 | 1.34 |
| 3 | Standard Care | 5.79 | 5.37 | 6.21 | 1.28 | 1.01 | 1.56 |
| 4 | Manuka | 5.34 | 4.87 | 5.83 | 1.14 | 0.9 | 1.36 |
| 4 | Marri | 5.46 | 4.99 | 5.9 | 0.97 | 0.76 | 1.2 |
| 4 | Placebo | 5.23 | 4.72 | 5.7 | 1.07 | 0.82 | 1.34 |
| 4 | Standard Care | 5.71 | 5.3 | 6.11 | 1.15 | 0.89 | 1.41 |
| 5 | Manuka | 5.27 | 4.78 | 5.72 | 0.97 | 0.73 | 1.22 |
| 5 | Marri | 5.39 | 4.87 | 5.89 | 0.97 | 0.75 | 1.2 |
| 5 | Placebo | 4.89 | 4.28 | 5.49 | 1.03 | 0.76 | 1.31 |
| 5 | Standard Care | 5.48 | 5 | 5.91 | 1.17 | 0.91 | 1.48 |
| 6 | Manuka | 5.09 | 4.55 | 5.6 | 0.61 | 0.41 | 0.81 |
| 6 | Marri | 5.03 | 4.46 | 5.57 | 0.69 | 0.5 | 0.89 |
| 6 | Placebo | 4.81 | 4.21 | 5.35 | 0.92 | 0.67 | 1.19 |
| 6 | Standard Care | 5.17 | 4.63 | 5.66 | 1.04 | 0.8 | 1.28 |
| 7 | Manuka | 4.32 | 3.75 | 4.84 | 0.51 | 0.35 | 0.69 |
| 7 | Marri | 4.61 | 4.01 | 5.16 | 0.5 | 0.32 | 0.7 |
| 7 | Placebo | 4.25 | 3.7 | 4.81 | 0.71 | 0.49 | 0.94 |
| 7 | Standard Care | 4.15 | 3.58 | 4.68 | 0.74 | 0.5 | 1 |
| 8 | Manuka | 3.36 | 2.79 | 3.9 | 0.35 | 0.22 | 0.52 |
| 8 | Marri | 3.52 | 2.96 | 4.14 | 0.33 | 0.16 | 0.51 |
| 8 | Placebo | 3.32 | 2.75 | 3.93 | 0.36 | 0.2 | 0.5 |
| 8 | Standard Care | 3.42 | 2.88 | 3.99 | 0.36 | 0.22 | 0.54 |
| 9 | Manuka | 2.62 | 2.07 | 3.13 | 0.26 | 0.12 | 0.41 |
| 9 | Marri | 2.64 | 2.09 | 3.25 | 0.21 | 0.08 | 0.36 |
| 9 | Placebo | 2.46 | 1.93 | 3.03 | 0.1 | 0.01 | 0.21 |
| 9 | Standard Care | 2.77 | 2.23 | 3.32 | 0.2 | 0.1 | 0.3 |
| 10 | Manuka | 1.81 | 1.33 | 2.27 | 0.12 | 0.03 | 0.23 |
| 10 | Marri | 1.84 | 1.32 | 2.38 | 0.11 | 0.03 | 0.22 |
| 10 | Placebo | 1.74 | 1.29 | 2.27 | 0.09 | 0.01 | 0.19 |
| 10 | Standard Care | 2.21 | 1.73 | 2.73 | 0.15 | 0.05 | 0.26 |
| 11 | Manuka | 1.11 | 0.75 | 1.53 | 0.09 | 0 | 0.2 |
| 11 | Marri | 1.39 | 0.92 | 1.87 | 0.04 | 0 | 0.1 |
| 11 | Placebo | 1.17 | 0.8 | 1.62 | 0.04 | 0 | 0.1 |
| 11 | Standard Care | 1.46 | 1.06 | 1.9 | 0.07 | 0.01 | 0.16 |
| 12 | Manuka | 0.86 | 0.54 | 1.23 | 0.05 | 0 | 0.14 |
| 12 | Marri | 0.92 | 0.56 | 1.35 | 0.03 | 0 | 0.07 |
| 12 | Placebo | 0.64 | 0.35 | 0.96 | 0.01 | 0 | 0.04 |
| 12 | Standard Care | 1.02 | 0.66 | 1.39 | 0.03 | 0 | 0.07 |
| 13 | Manuka | 0.55 | 0.32 | 0.82 | 0.03 | 0 | 0.08 |
| 13 | Marri | 0.59 | 0.28 | 0.93 | 0.01 | 0 | 0.04 |
| 13 | Placebo | 0.37 | 0.13 | 0.66 | 0 | 0 | 0 |
| 13 | Standard Care | 0.51 | 0.26 | 0.79 | 0.04 | 0 | 0.11 |
| 14 | Manuka | 0.34 | 0.16 | 0.53 | 0.01 | 0 | 0.04 |
| 14 | Marri | 0.37 | 0.1 | 0.75 | 0.01 | 0 | 0.04 |
| 14 | Placebo | 0.33 | 0.13 | 0.57 | 0 | 0 | 0 |
| 14 | Standard Care | 0.25 | 0.1 | 0.42 | 0.02 | 0 | 0.07 |

**References**

1. R Core Team A, Team RC. R: A language and environment for statistical computing. R Foundation for Statistical Computing, Vienna, Austria. 2012, 2022.

2. Van Buuren S, Groothius-Oudshoorn K. mice: Multiple Imputation by Chined Equations in R. *Journal of Statistical Software* 2009; **45**.

3. Huque MH, Carlin JB, Simpson JA, Lee KJ. A comparison of multiple imputation methods for missing data in longitudinal studies. *BMC Medical Research Methodology* 2018; **18:** 1-16.

4. Van Buuren S. *Flexible imputation of missing data*: CRC press, 2018.

5. Wood AM, White IR, Royston P. How should variable selection be performed with multiply imputed data? *Statistics in medicine* 2008; **27:** 3227-46.

6. Eekhout I, De Vet HC, Twisk JW, Brand JP, de Boer MR, Heymans MW. Missing data in a multi-item instrument were best handled by multiple imputation at the item score level. *Journal of clinical epidemiology* 2014; **67:** 335-42.

7. Commission CA. Revised codex standard for honey *Stan 12-1981, Rev. 1 (1987), Rev. 2 (2001). Codex Standard. 1981;12:1-7.*, 1987.

8. Locher C, Neumann J, Sostaric T. Authentication of honeys of different floral origins via high-performance thin-layer chromatographic fingerprinting. *JPC-Journal of Planar Chromatography-Modern TLC* 2017; **30:** 57-62.

9. Locher C, Tang E, Neumann J, Sostaric T. High-performance thin-layer chromatography profiling of Jarrah and Manuka honeys. *JPC-Journal of Planar Chromatography-Modern TLC* 2018; **31:** 181-9.

10. Green KJ, Dods K, Hammer KA. Development and validation of a new microplate assay that utilises optical density to quantify the antibacterial activity of honeys including Jarrah, Marri and Manuka. *PLoS One* 2020; **15:** e0243246.

11. Pappalardo M, Pappalardo L, Brooks P. Rapid and reliable HPLC method for the simultaneous determination of dihydroxyacetone, methylglyoxal and 5-hydroxymethylfurfural in leptospermum honeys. *PLoS One* 2016; **11:** e0167006.
